# “Purplish Blue” or “Greenish Grey”? Indigo Qualities and Extraction Yields from Six Species

**DOI:** 10.3390/plants13070918

**Published:** 2024-03-22

**Authors:** Anna Hartl, Andrea Polleichtner, Johannes Novak

**Affiliations:** 1 Working Group Knowledge Systems and Innovation, Institute of Organic Farming, Department of Sustainable Agricultural Systems, University of Natural Resources and Life Sciences, Vienna, Gregor-Mendel-Strasse 33, 1180 Vienna, Austria; 2 Working Group Soil Fertility and Cropping Systems, Institute of Organic Farming, Department of Sustainable Agricultural Systems, University of Natural Resources and Life Sciences, Vienna, Gregor-Mendel-Strasse 33, 1180 Vienna, Austria; 3 Institute of Animal Nutrition and Functional Plant Compounds, Department Farm Animals and Veterinary Public Health, University of Veterinary Medicine, Vienna, Veterinärplatz 1, 1210 Vienna, Austria; johannes.novak@vetmeduni.ac.at

**Keywords:** natural indigo, indigotin, natural dye, vat dye, ethnobotany, extraction yield, spectrophotometry, L*a*b*, colour measurement, indigo quality

## Abstract

Indigo quality is determined by its indigotin content. Another quality indicator is colour. For an evaluation of species, indigo samples from *Indigofera tinctoria*, *Indigofera suffruticosa*, *Indigofera arrecta*, *Persicaria tinctoria*, *Strobilanthes cusia* and *Wrightia laevis* cultivated in Austria and China were visually classified and analysed spectrophotometrically and using a L*a*b* measuring device. In addition to a standardised hot-extraction method without lime, some samples were extracted simulating traditional methods at ambient temperatures using lime. The highest indigotin contents were achieved with *Indigofera arrecta* (55%, Austria) and *Strobilanthes cusia* (56%, China). There were no statistically significant differences between the indigo extraction yields of the species cultivated in Austria, but *Indigofera arrecta* and *Persicaria tinctoria* had statistically significantly higher indigotin extraction yields than *Indigofera tinctoria* and *Indigofera suffruticosa*. From the species extracted in China, *Strobilanthes cusia* showed higher values in all parameters than *Indigofera tinctoria*, *Indigofera suffruticosa* and *Wrightia laevis*. Compared with the standardised method, the method simulating local practice yielded more indigo but had a lower indigotin content; the indigotin extraction yields did not differ greatly. L*a*b* values enabled precise estimations of the indigotin content, making it an interesting option for quality control, as inexpensive, easy-to-handle L*a*b* measuring instruments have become available.

## 1. Introduction

### 1.1. New Interest in an Ancient Dye

All over the world and for at least 6000 years, natural indigo has been used to dye textiles [1,2,3,4,5]. This plant extract, which contains the blue pigment indigotin as the main valuable component for dyeing textiles, became an important globally traded commodity from the 16th century onwards. Natural indigo production reached its peak with colonial mass production on plantations (“indigo *factories*”) between the 17th and 19th centuries, and, like cotton, it was closely linked to the human misery of slavery and other exploitative working conditions. In the late 19th and early 20th centuries, natural indigo was almost completely replaced by synthetic indigo [6,7]. What made synthetic indigo initially preferable over the natural product was its constant, standardised quality (indigotin content). Also, later, with the establishment of synthetic indigo production on an industrial scale, its price became cheaper than that of the natural product [8].

However, natural indigo in the traditional context is still being used today: it has been documented for specific ethnic groups, for example in China [9,10,11,12], India [13,14,15,16,17,18,19] and Indonesia [20,21], but is at risk of disappearing. Nevertheless, there is a renaissance of natural dyes as a sustainable alternative to synthetic dyes and as a means of generating local income in rural areas [22,23,24]. As the only plant-derived dye material to achieve long-lasting blues (and, in combination with other dyes, greens, violets and blacks as well [25]), natural indigo is regaining its significance. Apart from its use as a textile colourant, natural indigo (“*indigo naturalis*” in the Chinese Pharmacopoeia) is still used in traditional Chinese medicine for its anti-tumour, anti-inflammatory, anti-psoriatic, antibacterial and antiviral effects [26,27,28,29].

In addition to the blue pigment indigotin, natural indigo can contain other indigoid components (such as the isomers indirubin, cis-indigotin, isoindigotin and isoindirubin, or the intermediate product isatin), flavonoids, and plant-derived and mineral impurities [25,30,31]. The impurities result from particulate and colloidal materials from the leaf, dust and soil particles on the leaf surface, or from the use of lime during extraction [32,33]. While usually one component (indigotin) is considered the valuable component for dyeing, numerous valuable/bioactive components have been identified for medicinal purposes (indigotin, indirubin, tryptanthrin, isorhamnetin, etc. [26,28,29]).

### 1.2. A Cornucopia of Species and Manufacturing Methods

Thirty-one indigo-yielding species, comprising annual and perennial herbs, shrubs, lianas and trees from eight plant families, have been documented [25], but it is likely that there are many more, as the example of several indigo-yielding *Isatis* species [34,35] shows.

The following species have been very important economically and were introduced to many areas outside their natural distribution [25].

*Indigofera tinctoria* L., *I. suffruticosa* Mill., *I. arrecta* Hochst. ex A.Rich., *I. micheliana* Rose and *I. coerulea* Roxb. (Leguminosae) have been widely cultivated and globally transferred between the warm-temperate, subtropical and tropical regions of Africa, America and Asia [6,7,25]. Today, *Indigofera* species are still used in the context of traditional textiles (e.g., in China [10,12,36], Indonesia [20,21] and India [13,14,15,16,19,37,38]), but are also produced commercially for the domestic and/or global markets in Asia and in Latin America [22,23,24,39]. Woad (*Isatis tinctoria* L.; *Isatis indigotica* is currently considered to be a synonym [40]; Brassicaceae) was of great economic importance in Europe until the large-scale import of natural indigo from overseas [2,25]. *Isatis tinctoria* has recently been the focus of research on suitable indigo sources for European cultivation, together with *Persicaria tinctoria* (Ait.) H. Gross (Polygonaceae) [33,41]. *Persicaria tinctoria*, a major indigo-yielding species of East and Southeast Asia, was introduced to Europe, Russia and Ukraine in the 18th and 19th centuries [25]. It is still cultivated in China [11] and Japan [42,43]. Another indigo source of high significance in East, South and Southeast Asia is *Strobilanthes cusia* Kuntze (Acanthaceae) [9,10,11,12,13,14,17,18,19,36,44,45,46,47,48]. Due to its high yield of indigotin, it has been the focus of research on innovative extraction methods [49,50].

Besides these widespread species, many other indigo sources have been of local significance, such as the tree *Wrightia laevis* Hook.f. (Apocynaceae, traditionally used in Hainan, China [12]) and the liana *Marsdenia tinctoria* R.Br. (Asclepiadeae, distributed in South, Southeast and East Asia [13,25]).

**Figure 1:**
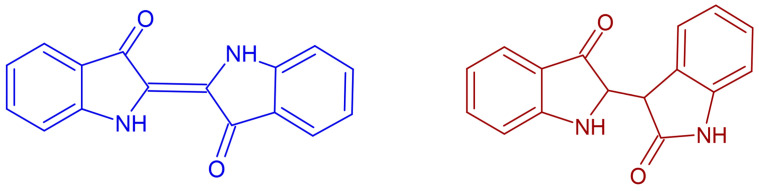
The blue pigment indigotin and the reddish component indirubin.

Plants do not contain indigotin, but instead indole glycosides as precursors (indican, isatan A, isatan B and others; some not yet identified) [25]. During the manufacturing process, the precursors are transformed into indoxyl, leading to the formation of the blue pigment indigotin and, in a side reaction, the reddish component indirubin (Figure 1). Natural indigo is mostly made by soaking the fresh plant material in water, removing the plants after a certain period, and then aerating the soaking liquid. After the precipitation of the suspended particles, the supernatant is siphoned off. The precipitate can be kept in a liquid state (“indigo paste”) or processed further (e.g., boiled, filtered off, pressed) and dried. Dry indigo is either sold solid (“indigo blocks”, “indigo cakes”) or as finely ground powder [25,33].

Indigo manufacturing processes differ mainly with regard to their extraction temperature and duration. While most historically documented methods [7,25,51] and traditional methods still practised in Asia extract at ambient temperatures, there are also methods involving higher temperatures that enable quicker extractions (detailed references in Section 3). Another crucial difference concerns the use of lime: slaked lime is used in large quantities in traditional manufacturing processes, for example in China [10,11,12] and Northeast India [17,18]. Only small quantities (lime water) were used in indigo production in British India at the end of the 19th century, and various other alkali were tested at that time as well [51]. The addition of lime increases the pH, accelerates the conversion of indoxyl to indigotin, and functions as a precipitant for the suspended particles after the aerating step [33,51]. However, lime or other alkaline substances are not necessary to produce indigo (and indeed have not been used at all by many producers). If not removed via acidic post-treatment, lime reduces the indigotin content through dilution [51].

### 1.3. True Blue? (Too) Many Methods for Determining Indigo Quality

The colour of the manufactured product has been an important quality indicator in indigo production and trade, not only in colonial times, as described in 19th- and early 20th-century sources [51,52], but today as well, for example, in local indigo paste markets in China [53]. Despite the cultural and temporal differences, the colour descriptions are very similar: for high qualities, various terms for blues with a reddish tinge are used (“deep violet-blue” [51], “reddish blue”, “purplish blue”, “violet”, “purple”, “violet blue”, “reddish violet” [52], “dark blue, deep purple-red” [53]), whereas low qualities are described as “dull and greyish” [51], “light blue”, “greenish”, “greenish grey”, “greyish blue” [52] and “light blue, bluish grey, turquoise” [53].

With the development of analytical methods, indigo quality (for the use of dyeing) was usually understood as the content of indigotin [51,52]. Analytical methods are manifold and there is still no standard, generally accepted method available (cf. critical review by P. John [33]). Furthermore, data in the literature also relate to different points of reference in indigo processing. Some studies have analysed the *content of indigotin precursors in fresh leaves* (some of these also calculated a probably too optimistic theoretical indigotin yield [54]), and other studies have analysed the *content of indigotin*, but by sampling at different processing stages (indigo suspension after aeration, centrifuged indigo, indigo paste, indigo powder, reduced indigo or indigo-dyed textile material), and referring either to the leaf material extracted or to the indigo extracted (details and references are provided in Section 3). In practice, extraction losses occur due to (a) the retention of indoxyl in the leaf tissue during the soaking of the plant material, (b) un-precipitated indigo that is lost when the supernatant is decanted, and (c) the conversion of indoxyl into components other than indigotin, such as indirubin (Figure 1) [33,51].

Therefore, it is difficult to compare species based on the available data, and only a few studies have directly compared two or more species in experimental research [34,35,55,56,57,58,59].

### 1.4. Aims of the Study

The objective of this study was to ascertain how indigo-yielding species differ in terms of indigo quality (indigotin content and colour) and extraction yields. Four well-known species—*Indigofera tinctoria*, *I. suffruticosa*, *Persicaria tinctoria* and *Strobilanthes cusia*—and two less well-known species—*Wrightia laevis* and *Indigofera arrecta*—were selected for this study. To the authors’ knowledge, there are no experimental data on the indigo yield and quality of *Wrightia laevis*. Ethnobotanic research has documented that traditional dyers consider its indigo to be of inferior quality to that of the other species used (own fieldwork in Hainan/China in 2019 and [12]). In contrast, *Indigofera arrecta* was a very promising indigo source during colonial times. In the early 20th century, when natural indigo producers tried to fight the competition presented by synthetic indigo by enhancing their production, it became a topic of agricultural research in India as a high-yielding species [8]. However, there is a lack of recent scientific data for this species as well.

A standardised mobile mini extraction (MME) method was developed and used for extractions during field research in China and subsequently for extractions from species grown in the research facility in Austria when field research was no longer possible as a result of the COVID-19 pandemic. A total of 69 samples were provided for spectrophotometric analysis. Wherever possible, indigo was analysed from two to three seed provenances per species. To consider extraction losses and achieve results closer to those in practice, the “final product” (indigo powder) was chosen instead of products at intermediate indigo-processing stages. The results are presented individually for each country since environmental differences between Austria and China did not allow a direct comparison of all species.

Additionally, 25 indigo samples from local producers in China, India and Indonesia were analysed to document and quantify the qualities achieved in practice. To demonstrate the effect of different extraction practices, two species (*Persicaria tinctoria* and *Indigofera arrecta*) were used to compare the standardised “hot”-extraction method without lime (MME) and a simulated local practice method at ambient temperatures using lime (sLPE).

Furthermore, the colours of the indigo samples were visually classified, measured (L*a*b* values) and correlated with indigotin contents to establish whether colour could still function as a quality indicator.

### 1.5. Definitions of Terms

The term “indigo” is applied in a confusing way and has different meanings not only in everyday language, but also in the scientific literature as well [60]. “Indigo” denominates the following:

(a) The plants used;
(b) The extract as a traded product;
(c) The valuable component for dyeing contained in the extract;
(d) The blue colour.

In the scientific literature dealing with both the plant extract and valuable component analysed, a variety of terms is used to differentiate the extract from the component, but often, the term “indigo” is used for both, and a precise meaning can be established only by understanding the context. Several publications explicitly mention the term “indigotin” as a synonym for “indigo” (Table A1). To add to the confusion, “indigotin” (also “indigotine”) can have a different meaning in the context of food chemistry, where it is a synonym for the blue food colourant E 132 (indigo carmine, C_16_H_8_N_2_Na_2_O_2_S_2_) [61,62].

In this study, the terminology of natural dye handbooks [25,51,63] is followed, and the term “indigo” is used exclusively for the plant extract and “indigotin” for its valuable component for dyeing. Where it is necessary to differentiate between the plant-derived and synthetic products, “natural indigo” and “synthetic indigo”, respectively, are used.

## 2. Results

### 2.1. Comparison of Species

#### 2.1.1. Species Cultivated in Austria

**Figure 2:**
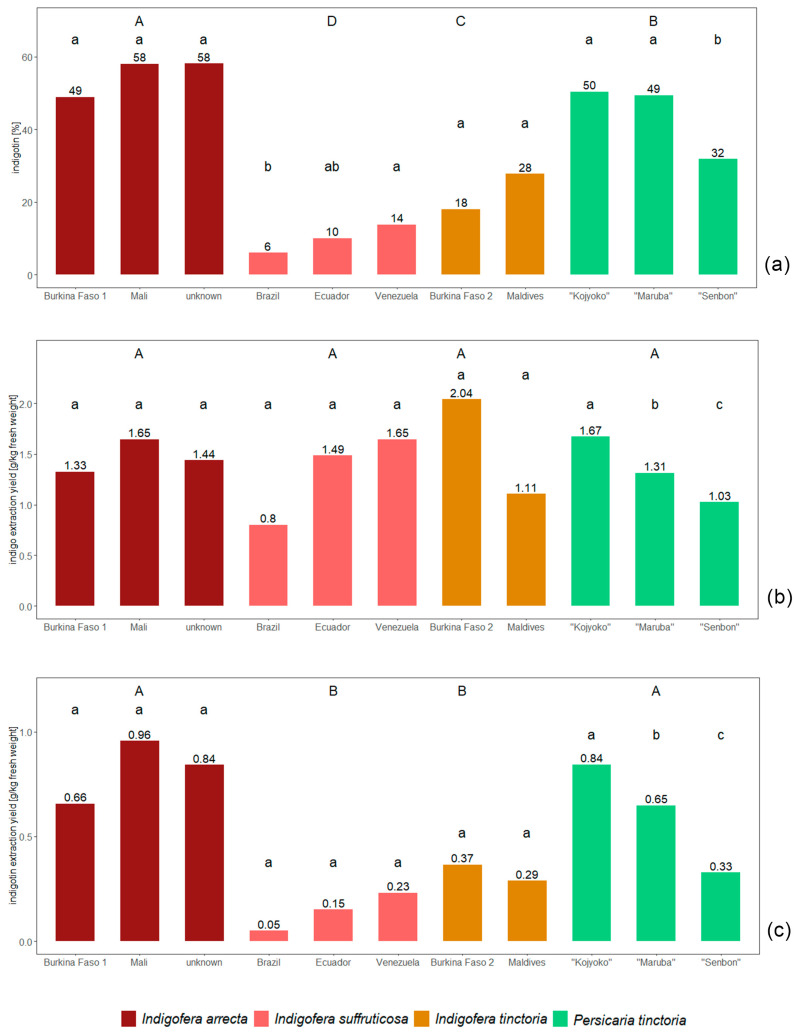
Comparison of species and seed provenances within each species, cultivated and extracted using the MME method in Austria: (**a**) content of indigotin in the indigo, (**b**) indigo extraction yield and (**c**) indigotin extraction yield. Values with the same letter do not differ significantly (ANOVA, *p* > 0.05; upper case letters: comparison of species; lower case letters: comparison of seed provenances within a species).

The four species cultivated in Vienna, Austria differed significantly from each other in their indigotin content. The highest content was achieved with *Indigofera arrecta* indigo (55% indigotin). Its amount was double and five times as much, respectively, as those of the two other *Indigofera* species (*Indigofera tinctoria*: 23%, *Indigofera suffruticosa*: 10% indigotin). The indigotin content of *Persicaria tinctoria* indigo was 44% (Figure 2, upper case letters).

The indigo extraction yields of the species did not differ significantly. The mean values of the provenances per species ranged from 1.3 g/kg (*Indigofera suffruticosa*) to 1.6 g/kg (*Indigofera tinctoria*).

The extraction yields of indigotin, however, differed significantly between two groups of species: *Persicaria tinctoria* and *Indigofera arrecta* (0.6 and 0.8 g/kg) showed much higher indigotin extraction yields than *Indigofera suffruticosa* and *Indigofera tinctoria* (0.1 and 0.3 g/kg). As there was no statistically significant difference in the indigo extraction yields, these two groupings must mainly be caused by the indigotin content.

Comparing different seed provenances within a species (Figure 2, lower case letters), the *Persicaria tinctoria* provenances showed statistically significant differences in almost all the parameters tested. In contrast, the *Indigofera arrecta* provenances did not differ significantly, but the data varied widely. The same was true for the provenances of *Indigofera tinctoria* and—apart from differences in indigotin content—for *Indigofera suffruticosa* as well. The results for *I. tinctoria* and *I. suffruticosa*, however, may be additionally influenced by the slightly different composition of the plant material extracted from one provenance of each species (Section 4).

#### 2.1.2. Species Cultivated in China

**Figure 3:**
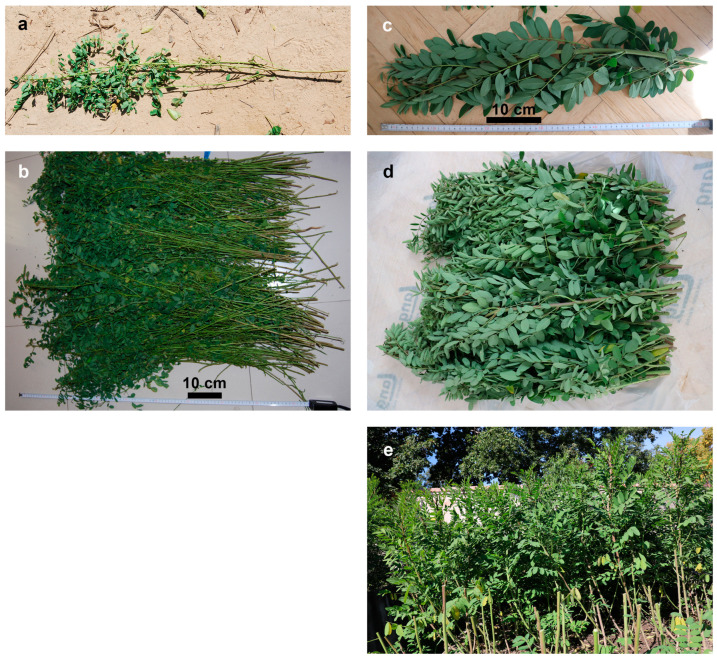
Composition of harvested plant material from *Indigofera* species cultivated in China and Austria. (**a**,**b**): *Indigofera tinctoria* from Yu Long, China. (**c**–**e**): *Indigofera suffruticosa*, provenance from Ecuador, cultivated in Austria. Pictures: A. Hartl.

The results from the extractions during fieldwork in three villages in China were evaluated separately from those cultivated and extracted in Austria owing to differences in species, environmental conditions and cultivation practices (Figure 3). In China, apart from in the case of *Wrightia laevis* (harvested from two trees, both growing in the same village), it was possible to extract only one provenance per species.

**Figure 4:**
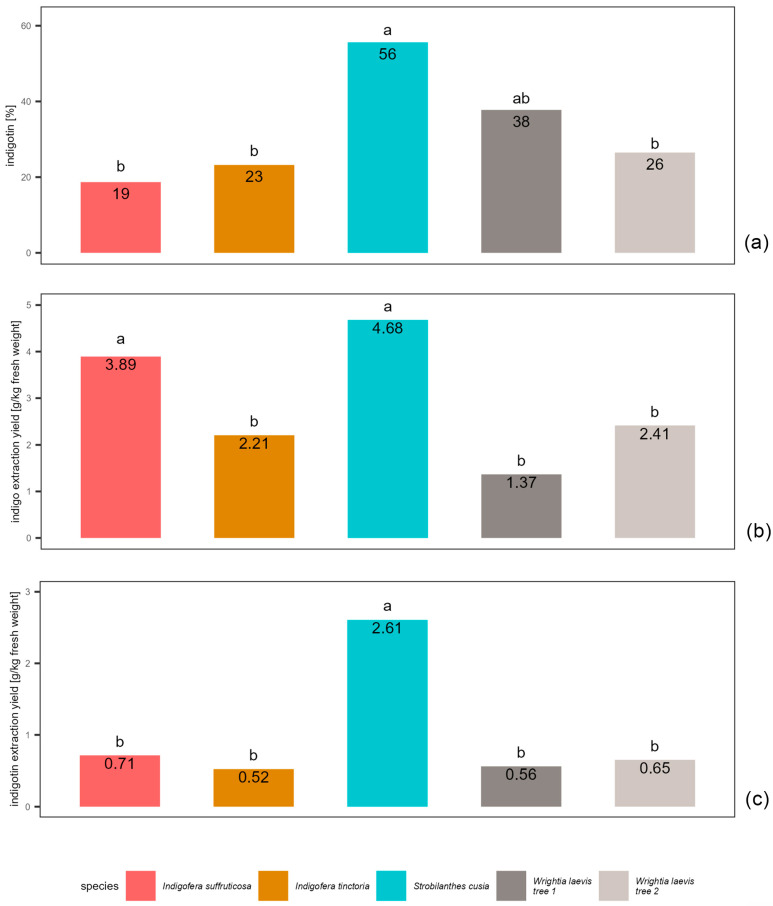
Comparison of locally cultivated species extracted using the MME method in China: (**a**) content of indigotin in the indigo, (**b**) indigo extraction yield and (**c**) indigotin extraction yield. Values with the same letter do not differ significantly (ANOVA; *p* > 0.05).

Compared with the other species extracted in China, *Strobilanthes cusia* showed the highest values for all the parameters tested. The differences between *Strobilanthes cusia* and the other species were also statistically significant in most cases (apart from the indigo extraction yield compared with *Indigofera suffruticosa* and the indigotin content compared with tree 1 of *Wrightia laevis*). Due to the high indigo extraction yield combined with a high indigotin content, the indigotin extraction yield of *Strobilanthes cusia* was up to five times greater than that of the other species (Figure 4).

### 2.2. Influence of Extraction Methods (MME, sLPE and Modifications) on Indigo Quality and Yield

The standardised method of extracting the plant material for three hours at 45 °C without using lime (MME) was compared with another extraction method (sLPE) that simulates local extraction practices at ambient temperatures with the addition of lime before the aeration step. The influence of these two methods was demonstrated using two provenances of *Indigofera arrecta* and three of *Persicaria tinctoria*, all cultivated in Austria. The extraction using lime (sLPE) yielded more indigo, but it had a much lower indigotin content than the indigo from the extraction without lime (MME). This was unsurprising since without any post-treatment the lime added during extraction remains in the extract and therefore dilutes the product. The differences in all the parameters analysed were statistically significant, except for the indigotin extraction yield of *Indigofera arrecta* (Table 1).

Further modifications of the extraction methods (mod.MME, mod.sLPE) were tested with one provenance of *Indigofera arrecta* to see whether there was scope for improvement. The extraction durations of both modified methods were determined using the “state” of the soaking liquid, recognisable by its change in appearance and smell, as was performed during colonial indigo production and is still practised today. Concerning the modified MME method, the soaking duration extended to five hours (rather than three hours), successfully increased both the indigo and indigotin extraction yields compared to the original MME method, even though the content of indigotin in the indigo remained the same (58%). The original sLPE method yielded rather greenish indigo with *Indigofera arrecta*, which was interpreted as low quality. Furthermore, the smell of the liquid at the end of the soaking period was already putrid, indicating “excessive fermentation”. The reduction of the soaking duration from 48 h to 28 h, which was determined by the appearance and smell of the liquid, did yield blue indigo with mod.sLPE, but the indigotin extraction yield remained more or less the same as with the original sLPE method. The increase in indigo yield combined with a decrease in indigotin content of the mod.sLPE method can be explained by the use of more lime (Table 1).

### 2.3. Indigo Quality of Local Practice Samples

In total, 25 indigo samples from local extraction practices were purchased from traditional small-scale producers in China during field research with local partners, and from project partners in India and Indonesia. Due to the different species, sites and extraction methods, the sample set was too heterogeneous and too small to be analysed statistically for differences in indigotin content, but it was very useful for providing comprehensive, quantitative documentation of indigo qualities achieved in practice and for a first test of the L*a*b* method.

**Figure 5:**
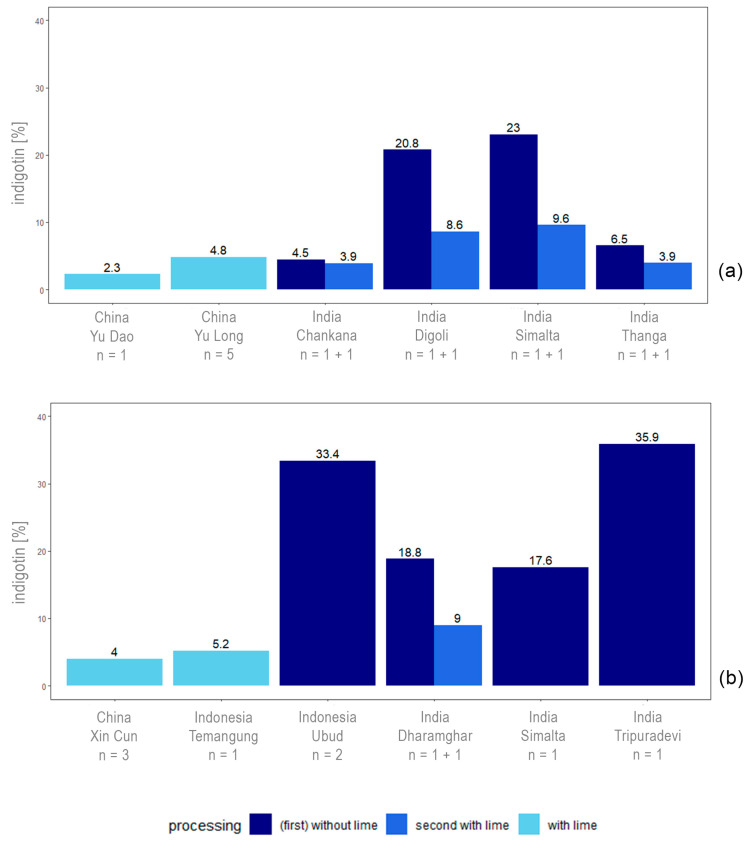
Content of indigotin achieved using local extraction practices. Indigo samples: (**a**) *Indigofera tinctoria* and (**b**) *Strobilanthes cusia.* Location is indicated by country and by village. The extraction methods were roughly classified into methods using lime and methods without lime. For the number of samples per village indicated (*n*), “*n* = 1 + 1” means two samples from the same extraction, one sample from the first processing without lime and one sample from the further processing using lime.

Locally produced indigo from *Indigofera tinctoria* had an indigotin content of 2–23% (Figure 5a). The maximum content was achieved through extractions without using lime in India and matched the indigotin contents achieved with the standardised extractions of this species in Austria (23%; Figure 2) and China (23%; Figure 4).

The indigotin content of locally produced indigo from *Strobilanthes cusia* ranged from 4 to 36% (Figure 5b). Again, the maximum indigotin content was from local extractions without the use of lime in India. However, the maximum remained 20% below that achieved using standardised extraction (56%; Figure 4).

The only sample of *Wrightia laevis* indigo obtained in accordance with local practice had an indigotin content of 2% (extraction with lime, Xin Cun, China).

### 2.4. Is Indigo Colour an Indicator of Indigo Quality?

#### 2.4.1. Visual Colour Classification of the Indigo Samples

The visual classification of the indigo samples showed a colour range from dark blue with a violet tinge to greenish shades when extractions with the sLPE method did not work properly. Light and mid blue shades occurred when lime was used during the extraction. In total, eight colour classes were identified, with most of the samples in the class “dark blue/violet” (Table 2; data of all samples: Tables S1 and S2).

#### 2.4.2. Measured Colour Parameters (L*a*b*)

The species differed significantly with regard to the L*a*b* values of the indigo (MME samples, Table A4). Within the provenances of each species, however, in most cases, there were no statistically significant differences (Table A5).

**Figure 6:**
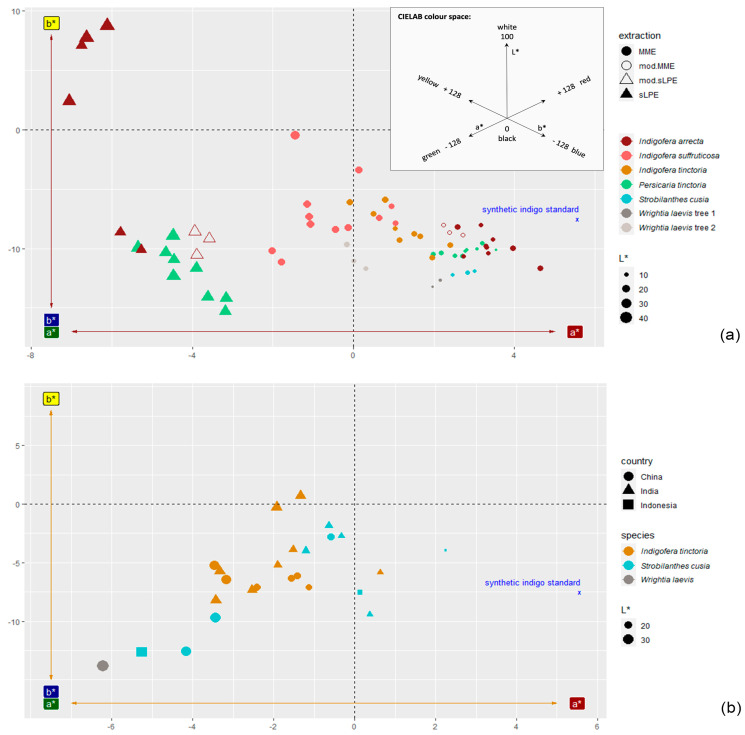
Positions of indigo samples in the colour space according to their L*a*b* coordinates, with synthetic indigo as the reference point. The three-dimensional CIELAB colour space is shown by the two axes of the complementary colours green–red (a* axis) and blue–yellow (b* axis); the third dimension, lightness (L* axis, for black–white), is indicated by the sizes of the data points (continuous scale); bigger sizes indicate higher L* values (i.e., lighter colours), smaller sizes indicate lower L* values (darker colours). (**a**) Samples from standardised extractions (three replications); the small, inserted graph shows the maximum range of the axes according to the *Free Colour Converter* provided by Nix Sensor Ltd., Hamilton, Canada (www.nixsensor.com, accessed 27 June 2023); (**b**) samples from local practice extractions.

The positions of the indigo samples’ colours in the colour space (Figure 6) can easily be understood by imagining their colours described with the visual colour classification. For example, samples visually classified as “dark blue/violet” (such as all the samples extracted using the MME method from *Indigofera arrecta*, *Persicaria tinctoria* and *Strobilanthes cusia*, Table S1) are positioned between blue (negative b*-axis) and red (positive a*-axis), further away from the centre. As they are dark, they have low L* values. Blues without a reddish tinge (“dark blue”, “mid blue”, “light blue”) are positioned further away from red (negative a*): the light and mid blue samples (such as the sLPE samples from *Persicaria tinctoria* or the mod.sLPE samples from *Indigofera arrecta*) are further away from the centre and have higher L* values than the dark blue samples (such as the MME samples of *Indigofera suffruticosa* positioned further left). “Green” samples (from malfunctioning sLPE extractions of *Indigofera arrecta*) are positioned in the area between green and yellow (negative a* and positive b* axes), almost opposite the dark blue/violet colour (Figure 6a).

Considering the results from the indigotin analysis (Figure 2a and Figure 4a), it seems that the species that showed high indigotin contents in their indigo when extracted using the MME method (*Indigofera arrecta*, *Persicaria tinctoria* and *Strobilanthes cusia*) are positioned closer to the synthetic indigo standard, whose colour is also a dark blue with a violet tinge. Samples with a lower indigotin content, especially those from sLPE extractions, are positioned further away from the synthetic indigo standard and are lighter (high L* values) due to the remaining lime in the indigo.

Only a few samples from local practice extractions (Figure 6b) show positive a* and negative b* values (“dark blue/violet”). They are not as close to the synthetic standard as some samples from the MME extractions. Most of the local practice samples are positioned in the area between the negative b* and negative a* axes and show “dark blue/black”, “dark blue” and “mid blue” colours. The sample with by far the highest L* value and that is furthest from the centre is “light blue” (*Wrightia laevis*).

#### 2.4.3. Correlation between Indigo Colour (L*a*b* Values) and Indigotin Content

**Figure 7:**
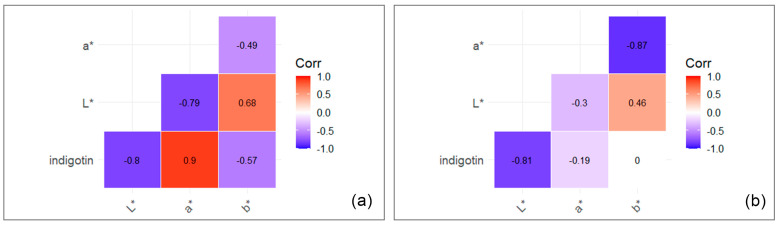
Correlation between the indigotin content and L*a*b* values of indigo samples: (**a**) extraction with the MME method and (**b**) extraction with the sLPE method. Pearson correlation coefficients. The aggregated data set was used.

The correlation between the measured colour coordinates and the indigotin content was analysed using data from the standardised extractions performed in Austria and China (MME and sLPE). For both extraction methods, there was a strong negative correlation between indigotin content and the L* value. Furthermore, the two extraction methods differed greatly in terms of their correlation patterns: while MME samples showed a strong positive correlation between indigotin content and a* values, in sLPE samples, the indigotin content and a* values were not correlated. MME samples showed a strong positive correlation between a* and L* values, whereas sLPE samples showed a strong negative correlation between a* and b* values (Figure 7).

#### 2.4.4. Can Indigotin Content Be Predicted by Measuring Indigo Colour?

Multiple linear regression was used to test if all L*a*b* values and their interactions together could significantly predict the indigotin content. The overall regression was statistically significant (R^2^ = 0.89, F (7, 60) = 66.09, *p* < 0.001, Table 3). L*, a* and the interaction between L* and a* predicted the indigotin content significantly (all *p* < 0.01).

## 3. Discussion

### 3.1. Indigotin Content and Indigo(tin) Extraction Yield

The lack of a standardised method for determining indigotin and the wide variety of reference points used in previous studies (dry indigo, indigo paste, indigo suspension, etc.) allow only a limited comparison of results. Comparable data are often not available, even for a widely used species such as *Indigofera tinctoria.* An analysis of extraction yields with those of previous studies is even more limited because data also refer to different compositions of the plant material extracted (leaves and stems versus leaves only) and sometimes refer to the dry weight of plant material, even though the extraction was performed using fresh plant material (Table 4). The composition of the plant material extracted is of relevance because in most species, indigo precursors are present only in the leaves [25]. However, it has been demonstrated with *Strobilanthes cusia* that even stems contain precursors that can be extracted in a separate process using specific methods [49].

The highest indigotin content maxima in indigo documented in previous studies (including additional species not analysed in the present study; Table 4) are about 40%. The highest indigotin content (57%) achieved was in just one study [50], in which *Strobilanthes cusia* was extracted in an elaborated two-step method using nitrogen gas instead of oxidation by air, and the indigo was subsequently purified by removing indirubin with acetone extraction. In most studies, the indigotin content maxima barely reached 15%. The results of the present study, however, were in most cases far above 15%, and the highest values (58% with two *Indigofera arrecta* provenances and 56% with *Strobilanthes cusia*) matched the maxima of earlier studies, even though the MME method is a much simpler method than that used by Jin et al. [50]. However, there might still be plenty of scope for improvement in view of the indigotin contents documented in 19th-century sources: indigos with less than 20% indigotin were classified as the *lowest* qualities, while the best indigo qualities contained 70–90% indigotin [52]. Unfortunately, these data do not provide information about the analytical methods or indigo-yielding species used.

With regard to the indigo extraction yield, only one study on *Persicaria tinctoria* provides results in a form that is more comparable: the indigo extraction yields achieved by Bechtold et al. ranged from 1.0 to 7.9 g/kg, and the maximum reached by one extraction was 13.0 g/kg [54]. These results are generally much higher than the maximum achieved in the present study’s extractions of *Persicaria tinctoria* using the MME method (1.7 g/kg, provenance “Kojyoko”, Figure 2) but also above the maximum indigo extraction yield of all the species evaluated (4.7 g/kg achieved with *Strobilanthes cusia*, Figure 4).

The indigotin extraction yields were within the range achieved in most previous studies with comparable reference points, apart from two studies that document much higher indigotin extraction yields achieved with *Strobilanthes cusia* (4.7 g/kg [59] and 4.5 g/kg [50]) and *Indigofera tinctoria* (3.3 g/kg [59]).

The seed provenances of the three *Indigofera* species cultivated and extracted in Austria originated from wild collections, apart from one *Indigofera arrecta* provenance, for which its origin is unknown (Table A2). As *Indigofera tinctoria*, *I. suffruticosa* and *I. arrecta* have been intensively cultivated for centuries, it is possible that other seed provenances of plants in cultivation produce higher yields due to breeding and/or adaptation to specific sites. Statistically significant differences between different seed provenances concerning precursor or indigotin content and extraction yields have been documented in previous studies with *Indigofera tinctoria* [58,64,65], *Isatis tinctoria* [66,67] and *Persicaria tinctoria* [68], while another study with *Persicaria tinctoria* could not confirm statistically significant differences [69]. To date, studies evaluating *Strobilanthes cusia* provenances are still lacking (or are not accessible).

In the search for seeds for the cultivation and extraction experiments, several were incorrectly declared to be “*Indigofera tinctoria*”, even in specialised seed trade and botanic gardens. As the genus *Indigofera* is huge (750 species [70]) and precise species identification is challenging, cooperation with experienced botanists is highly recommended.

### 3.2. The Evaluation of Indigo-Yielding Species Requires a Differentiated Approach

Of the species extracted using the MME method in China, *Strobilanthes cusia* showed the best results concerning indigotin content and extraction yields. Of the species cultivated and extracted in Austria, *Indigofera arrecta* had the highest indigotin content and, together with *Persicaria tinctoria*, the highest indigotin extraction yield (regarding indigo extraction yields, there were no statistically significant differences between the species tested in Austria).

The superiority of *Strobilanthes cusia* compared with other indigo-yielding species has been highlighted by many traditional producers/dyers interviewed in ethnobotanical studies in China [10,12] and has also been documented in experiments comparing *Strobilanthes cusia* with *Indigofera tinctoria* [59]. The results of the present study indicate that the superiority of *Strobilanthes cusia* could be due to a high content of indigotin in the indigo, combined with a high indigo extraction yield. A high indigotin content alone, however, can also be achieved with other species, especially if extraction methods are optimised. Nevertheless, the indigo extraction yield of those species is much lower or, as in the case of *Indigofera suffruticosa* extracted in China (MME), is also high but the content of indigotin is low.

Therefore, when comparing indigo-yielding species, it is important to differentiate between the perspective of the indigo *user* (content of indigotin) and the perspective of the indigo *producer* (indigo and indigotin extraction yield per kg of plant material and also per hectare per year). For medicinal purposes, other components found in indigo are also relevant, and for dyers this may also be the case if minor components contribute to specific shades of blue.

### 3.3. Indigofera arrecta—A “Forgotten Species” with a Promising Future?

Indigo from the three *Indigofera arrecta* provenances cultivated in Austria showed the same indigotin content as that from *Strobilanthes cusia* cultivated in Hainan (MME method). The indigotin content and indigotin extraction yield of *I. arrecta* were much higher than those of the two widely used *Indigofera* species, *I. tinctoria* and *I. suffruticosa*, irrespective of whether these were cultivated in Austria or tropical Hainan.

These promising results explain why *Indigofera arrecta*, native to tropical and southern Africa and the Arabian Peninsula [71], was widely transferred to indigo-producing areas in the 19th century: first to Java (named “Natal indigo” according to its origin) and then from there—becoming “Java indigo”—to India [2]. Due to its high yields, it played an important role in the attempts of natural indigo producers in colonial India to fight the competition presented by synthetic indigo in the late 19th/early 20th century [8]. As the present results show, *Indigofera arrecta* could again become an interesting alternative source of indigo in (sub)tropical regions. Its potential use, however, might not be restricted to these areas only.

In previous studies searching for indigo sources suitable for production in temperate regions, *Persicaria tinctoria* showed better results than *Isatis tinctoria* with regard to precursor or indigotin content and extraction yields, both at the laboratory scale [56] and pilot/prototype scale ([55], Table 4). In the present experiments (MME), *Indigofera arrecta* indigo had a 10% higher indigotin content than *Persicaria tinctoria*, and its indigo and indigotin extraction yields did not differ significantly from those of *Persicaria tinctoria*.

Being a plant whose natural habitats are moist valleys and stream banks [72], *Persicaria tinctoria* has been cultivated in humid regions, such as in Tokushima Prefecture, Japan [25,42] (according to the updated Köppen–Geiger climate classification, Tokushima is in a warm temperate climate zone, fully humid (i.e., rain at all seasons) and with hot summers [73]). Otherwise, *Persicaria tinctoria* requires irrigation as it is sensitive to drought stress [56]. With ongoing climate change, the irrigation of field crops will become increasingly challenging. *Indigofera arrecta*—which, as demonstrated here, can be grown as an annual crop in temperate climate conditions—is possibly more tolerant to dry conditions than *Persicaria tinctoria*. However, this still needs to be proven in experimental research, as data concerning its requirements of minimum annual precipitation [74] are rare and contradictory, and there is an absence of data concerning the annual distribution of precipitation. Another potential advantage of *Indigofera arrecta* compared with *Persicaria tinctoria* concerns nutrient supply: *Persicaria tinctoria* requires intense fertilising (cultivation instructions for conventional farming [75] recommend 150–200 kg N/ha), while *Indigofera arrecta* as a leguminous species has the advantage of symbiotic nitrogen-fixing bacteria and positive effects in crop rotation [74].

### 3.4. The Ambivalence of Using Standardised Methods, and Potential Improvements

As a first step, the standardised methods were useful for comparing indigo-yielding species, but they also represent a simplification and compromise in terms of species-specific requirements. A standardised extraction method does not mean an optimal extraction method.

The MME method was developed as a transportable field method in pre-experiments with *Persicaria tinctoria* and adapted after field testing with the same species and *Strobilanthes cusia*. For all species except *Wrightia laevis*, this method worked well. With *Wrightia laevis*, the standardised extraction duration would have yielded no indigo at all, but when the soaking duration was extended, neither the indigotin content nor extraction yield were significantly different from that of *Indigofera tinctoria* or *I. suffruticosa*.

In most species, precursors are present in the leaves. The leaves of indigo-yielding species vary in size and consistency, so it could be that the “extraction behaviour” is species specific. Furthermore, other factors may be also species specific, such as the types of precursors, the microbiome and enzymes of the plants [25], or there are additional substances present in leaves that could have an impact on the process (*Wrightia laevis*, for example, is the only species tested that contains a milky sap, as observed during extractions).

In colonial times, indigo producers noticed that it was necessary to adapt the soaking time, in relation not only to different species, but also to the age and storage duration of the harvested plants before extraction and to the ambient temperature during extraction [52]. Practitioners have always been aware of this and have defined durations of processing steps rather by the observation of visible, olfactory or haptic changes during the process (such as a change in the colour or smell of the extraction liquid, the occurrence of bubbles, etc.) [7,10,12,52,76].

The influence of extraction methods on indigotin content and/or extraction yields has been demonstrated in studies with *Indigofera tinctoria* [77], *Isatis tinctoria* [55,78], *Marsdenia tinctoria* [58], *Persicaria tinctoria* [55] and *Strobilanthes cusia* [49,50,59,79] (Table 4).

In future research, one way of addressing the dilemma so that species are more likely to be compared under their species-specific optimal extraction conditions could still be to use a standardised extraction method, but rather than standardising duration, the threshold values of other process parameters should be standardised instead.

**Table 4:** Range of indigo quality and extraction yields. Minimum and maximum mean values achieved in laboratory experiments; the results from pilot or prototype extractions are indicated separately (*data in italics:* min/max values if extraction experiments were carried out without replications). The original data were transferred into comparable units (not included are data with other reference points, such as extraction yields referring to area (kg/ha) or percentage in weight per volume (*w*/*v*)). Only data from an indigotin analysis of indigo are reviewed; theoretical indigotin calculations from precursor analyses are not included.

**Species**	**Indigotin Content** [% in indigo]	**Indigo Extraction Yield** [g/kg plant material]	**Indigotin Extraction Yield** [g/kg plant material]	**Parameters Tested and Extraction Method Applied**	**Analytical Method** (in brackets: indigo processing state sampled/solvent used/nm measured)	**Source**
*Indigofera* *tinctoria*	n.d.	n.d.	0.58–0.75 (per fw 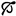 )	Test of agronomic factors (four ecotypes, three levels of deficit irrigation). Extraction method: boiling, cooling, adding Ca(OH)_2_ solution, aerating, settling, post-treatment with HCL, centrifuging suspension (laboratory scale).	Spectrophotometry (indigo suspension, centrifuged/H_2_SO_4_/611 nm)	[64]
	n.d.	n.d.	6.50–6.80 (per fw 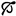 )	Test of agronomic factors (four ecotypes, three planting days). Extraction method: boiling, cooling, adding Ca(OH)_2_ solution, aerating, settling, post-treatment with HCL, centrifuging suspension (laboratory scale).	Spectrophotometry (indigo suspension, centrifuged/H_2_SO_4_/611 nm)	[65]
	n.d.	*268.30*^1^ (paste per fw ⌘)	*3.27* ^1^ (per fw ⌘)	Comparison of *I. tinctoria* and *Strobilanthes cusia.* Extraction method: ambient temperature, adding Ca(OH)_2_ solution, aerating, washing precipitated indigo with Ca(OH)_2_ solution, centrifuging (laboratory scale).	Spectrophotometry (indigo paste, centrifuged/H_2_SO_4_/611 nm)	[59]
	1.69–2.65 (in indigo paste)	11.90–18.60 (indigo dry per fw ⌘)	n.d.	Test of four extraction methods without lime (*conventional* at ambient temperature and boiling the indigo paste, *microbial* with effective microorganisms, *hot water* at 60 °C subsequently mixed with cold water to obtain 38 °C and *chemical* with concentrated ammonia solution and calcium chloride) and six soaking durations (laboratory scale).	Spectrophotometry (indigo paste, centrifuged/H_2_SO_4_/611 nm)	[77]
	n.d.	1.03–1.09 (indigo dry per fw 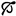 )	n.d.	Test of four different *I. tinctoria* provenances. Comparison of *I. tinctoria* and *Marsdenia tinctoria*. Extraction method (without lime): extracting at ambient temperature, aerating, filtering off with vacuum pump, washing indigo with water, drying (laboratory scale).	No chemical analysis	[58]
*Indigofera* *suffruticosa*	17.31–43.40 (in dry indigo)	20.00–34.00 (indigo dry per dw 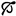 )	? ^2^–11.20 (per dw 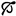 )	Test of agronomic factors (documentation of growing cycle, effects of age at harvest time). Extraction method (without lime): extracting at 25 °C, aerating, cooling and settling, decanting, centrifuging suspension, drying, grinding (laboratory scale).	HPTLC-densitometry (indigo powder/3:2 mixture of toluene and ethyl acetate; N, N-dimethylformamide/610 nm)	[80]
*Isatis tinctoria* (incl. *I. indigotica*)	n.d.	n.d.	0.30–0.80 (per fw 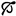 )	Test of agronomic factors (sowing date, plant density, N fertilisation, seedling transplantation, irrigation rate). Extraction method: boiling, cooling, adding Ca(OH)_2_ solution, aerating, settling, post-treatment with HCl (laboratory scale).	Spectrophotometry (indigo suspension/ethyl acetate/600 nm)	[81]
	0.00–0.01	n.d.	n.d.	Test of agronomic factors (N fertilisation, harvest time). Extraction method (without lime): hot extraction, pH control (NaOH), aerating, settling, washing indigo precipitant with water, filtering, drying, grinding (laboratory scale).	Spectrophotometry (indigo powder/glacial acetic acid/664 nm)	[82]
	n.d. (lab) *5.70*–*12.90* (pilot) *4.80*–*10.00* (prototype) (in dry indigo)	n.d. (lab) *0.90*–*3.90* (pilot) *0.60*–*2.60* (prototype) (indigo dry per fw 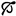 )	*1.30–2.00* (lab) (per fw 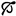 ) *0.11*–*0.22* (pilot) *0.05*–*0.26* (prototype) (per fw 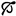 )	Development of extraction protocols (laboratory scale) and upscaling (pilot and prototype). Test of extraction methods: leaf-to-water ratio, extraction temperature (hot and ambient; cooling), extraction duration, pH control (lime), aeration duration, post-treatment of indigo precipitate with acid, etc.	Spectrophotometry (indigo suspension/ethyl acetate/600 nm) and microgravimetric method (indigo powder)	[55]
	n.d. 20–40 (pilot) ^3^	n.d. n.d.	0.02–2.10 (lab) n.d. (per fw 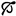 )	Development of extraction methods (laboratory scale) and test at pilot scale: test of different extraction temperatures, pH adjustment (HCl, sulphuric acid; ammonia solution), cooling, aerating (only at pilot scale: settling, filtering, drying).	Spectrophotometry (indigo suspension/ethyl acetate/600 nm)	[78]
	n.d.	n.d.	0.74–6.52 (per fw 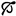 )	Comparison of *I. tinctoria* and 14 other *Isatis* taxa. Extraction method: boiling, cooling, pH-adjustment ^4^, aerating, post-treatment with HCl (laboratory scale).	Spectrophotometry (indigo suspension/ethyl acetate/600 nm)	[35]
	n.d.	n.d.	0.74–4.19 (per fw 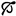 )	Comparison of *I. tinctoria* and three other *Isatis* sp. at different harvest times. Extraction method (without lime): boiling, cooling, pH adjustment (NaOH), aerating, settling, post-treatment with HCl (laboratory scale).	Spectrophotometry (indigo suspension/ethyl acetate/600 nm)	[34]
Several other *Isatis* sp.	n.d.	n.d.	0.01–10.00 (per fw 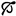 )	Comparison of 14 *Isatis* taxa and *I. tinctoria.* Extraction method: boiling, cooling, pH-adjustment ^4^, aerating, post-treatment with HCl (laboratory scale).	Spectrophotometry (indigo suspension/ethyl acetate/600 nm)	[35]
	n.d.	n.d.	not detected–2.53 (per fw 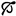 )	Comparison of three *Isatis* sp. And *I. tinctoria* at different harvest times. Extraction method (without lime): boiling, cooling, pH adjustment (NaOH), aerating, settling, post-treatment with HCl (laboratory scale).	Spectrophotometry (indigo suspension/ethyl acetate/600 nm)	[34]
*Marsdenia* *tinctoria*	n.d.	0.12–0.25 (indigo dry per fw 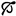 )	n.d.	Comparison of *M. tinctoria* and *Indigofera tinctoria.* Test of extraction methods (without lime): different temperatures (ambient, hot) and steeping durations; aerating, filtering off with vacuum pump, washing indigo with water, drying (laboratory scale).	No chemical analysis	[58]
*Persicaria* *tinctoria*	<*2.00*–*9.20* (in dry indigo)	*1.00–13.00* (indigo dry per fw ⌘)	*?*^5^–*0.40* (per fw ⌘)	Test of different storage conditions of harvested plant material. Extraction method (without lime): plant material was extracted twice (hot), storing extracts for 4–5 days with aerating once a day, adding CaCl_2_·2H_2_O to improve precipitation, filtering sediment, drying, grinding. Two analytical methods applied.	Spectrophotometry (indigo powder, measuring *leuco*-indigotin/reducing agent: aqueous alkaline solution of Fe(II)triethanolamine/410 nm)	[54]
	*0.00–12.30* (in dry indigo)		*<0.01–0.69* (per fw ⌘)		Standard exhaust dyeing experiments and spectrophotometry (indigo-dyed textile)	
	n.d. (lab) *17.40–24.30* (pilot) *5.28–10.44* (prototype) (in dry indigo)	n.d. (lab) *3.20–7.80* (pilot) *1.80–10.00* (prototype) (indigo dry per fw ⌘)	7.00–10.00 (lab) (per fw 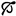 ) *0.70–1.36* (pilot) *0.18–0.89* (prototype) (per fw ⌘)	Development of extraction protocols (laboratory scale) and upscaling (pilot and prototype). Test of extraction methods: leaf-to-water ratio, extraction temperature (hot and ambient; cooling), extraction duration, pH control (lime), aeration duration, post-treatment of indigo precipitate with acid, etc.	Spectrophotometry (indigo suspension/ethyl acetate/600 nm) and microgravimetric method (indigo powder)	[55]
*Strobilanthes* *cusia*	28.70–43.50 (lab) 45.20 (56.70 ^6^) (pilot) (in dry indigo)	n.d. (lab) n.d. (pilot)	4.00–4.50 (lab) n.d. (pilot) (per fw 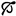 )	Development of extraction method at laboratory and pilot scales. Test of effect of dissolved oxygen concentration on indigotin and indirubin formation; evaluation of indigotin and indirubin production under anaerobic conditions in nitrogen gas environment compared with conditions with and without aeration; ambient temperature, pH adjustment with NaOH.	HPLC equipped with UV detector (indigo powder/dimethyl sulfoxide, A: water-acetic acid, B: acetonitrile/280 nm)	[50]
	n.d.	n.d.	0.85 (1.16 ^7^) (per fw ⌘)	Development of two-step extraction process (laboratory scale): separate extraction of leaves (conventional extraction, pH adjustment) and stems (microwave drying, ethanol extraction, use of exogenous enzymes, pH adjustment).	HPLC equipped with UV detector (centrifuged indigo/A: water–acetic acid, B: acetonitrile/280 nm)	[49]
	n.d.	*98.60–156.90* (paste per fw ⌘)	*0.08–4.72* (per fw ⌘)	Comparison of *Strobilanthes cusia* and *I. tinctoria.* Only with *Strobilanthes cusia:* test of extraction methods: different plant material conditions (fresh, semi-dried, dried), different soaking durations; extraction at ambient temperature, adding Ca(OH)_2_ solution, aerating, washing precipitated indigo with Ca(OH)_2_ solution (lab scale).	Spectrophotometry (indigo paste, centrifuged/H_2_SO_4_/611 nm)	[59]

(^1^) Only one experiment performed. (^2^) “?”: minimum data not provided in the study. (^3^) Pilot scale: estimated based on laboratory-scale results. (^4^) Typing error in the source quoted: “Na(OH)_2_” which should be either NaOH or Ca(OH)_2_. (^5^) “?”: minimum data not calculated in the study when indigotin content <2%. (^6^) After purification (removal of indirubin with acetone). (^7^) Total indigotin yield of separate two-step extraction of leaves and stems. Abbreviations: dw = dry weight, fw = fresh weight, 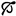 = leaves, ⌘ = leaves and stems, n.d. = not determined, HPLC = high-performance liquid chromatography, HPTLC-densitometry = high-performance thin-layer chromatography–densitometry.

### 3.5. Colour Measurement as a Possible New Method to Determine Indigo Quality

The results of the present study confirm the relationship between indigo colour and indigo quality, as is already known from practice [51,52,53]. The regression analysis of the L*a*b* colour coordinates and the indigotin content of indigo samples determined spectrophotometrically showed that it is possible to predict the content of indigotin, provided that all three colour coordinates, including their possible interactions, are considered in the calculation. Improved standardisation with more exact laboratory methods (e.g., HPLC) could result in a better prediction of indigotin. Since cheap handheld L*a*b* measurement devices are available and their measurement of indigo is fast without tedious sample preparation, it may become an interesting option for routine analyses.

## 4. Materials and Methods

Standardised extractions yielding 69 indigo samples were performed during ethnobotanical field research in China with locally cultivated species and with species cultivated in Austria (*Indigofera tinctoria*, *I. suffruticosa*, *I. arrecta*, *Persicaria tinctoria*, *Strobilanthes cusia* and *Wrightia laevis*). In addition, 25 local indigo samples from indigo producers in China, India and Indonesia were analysed (*Indigofera tinctoria*, *Strobilanthes cusia* and *Wrightia laevis*). In total, 94 indigo samples from six species were analysed (Table 5, Tables S1 and S2). The number of indigo samples per species varied between 7 and 23.

### 4.1. Indigo Samples from Standardised Extraction

#### 4.1.1. Plant Material for Extractions during Field Research in China

In November 2019, field research on traditional indigo use was carried out in southern China. With prior informed consent from the interviewees, plant material from locally cultivated *Indigofera tinctoria*, *Indigofera suffruticosa*, *Strobilanthes cusia* and *Wrightia laevis* was harvested according to local practice (*Indigofera* species: stems with leaves cut a few centimetres above the ground; *Strobilanthes cusia*: stems with leaves, thick old stems removed; *Wrightia laevis*: twigs up to ~40 cm length with leaves). For the extractions, a mixed sample per field or tree was used.

#### 4.1.2. Plant Material of Species Cultivated in Vienna

Indigo-yielding plants were cultivated in 2021 in the garden of Schönbrunn Palace (Vienna, Austria), in close cooperation with the *Horticultural College and Research Center and Austrian Federal Gardens—Botanical Collections*.

To obtain the seeds of indigo-yielding species, 24 botanic gardens (EU, UK, USA, Singapore, China, Japan) and the international membership organisation *Botanic Gardens Conservation International* (BGCI), as well as 11 specialised seed traders (EU, USA) and three indigo producers (UK, El Salvador, France) were contacted. The necessary phytosanitary documents for the import of seeds and cuttings were obtained. None of the species are CITES-listed. The requirements concerning the *Convention on Biological Diversity*/*Nagoya Protocol on Access and Benefit-sharing* were considered: material supply agreements were signed with the *Royal Botanic Gardens, Kew*, UK; the *Botanical Garden of the Paris Lodron University of Salzburg*, Austria; *Bonn University Botanic Gardens*, Germany; and *Singapore Botanic Gardens*, Singapore. Original species were identified by acknowledged experts (*Indigofera* species: Dr Brian Schrire, Honorary Research Associate, *Accelerated Taxonomy Department, Royal Botanic Gardens, Kew*, UK; *Strobilanthes* species: Dr John R. I. Wood, Department of Biology, University of Oxford and H.R.A. Royal Botanic Gardens, Kew, UK). Voucher specimens were scanned (Supplementary Material) and deposited at the herbarium of the *Institute of Botany, University of Natural Resources and Life Sciences, Vienna*, Austria. Of 16 seed provenances, five turned out to be wrongly declared and did not yield any indigo (species identified as *Strobilanthes hamiltoniana* (Steud.) Bosser & Heine, *Indigofera heterantha* Wall. ex Brandis and *Indigofera zollingeriana* Miq.), and one provenance did yield indigo but turned out to be *Indigofera arrecta* instead of *I. tinctoria*. Eventually, 11 provenances of the four species *Indigofera tinctoria*, *I. suffruticosa*, *I. arrecta* and *Persicaria tinctoria* could be used.

The cultivation methods used for *Persicaria tinctoria* were based on technical instructions [75] and previous research in Austria, e.g., [68]. The cultivation of *Indigofera* species was more challenging as only a few seeds per provenance were available, and instructions for cultivation in temperate regions (if available at all) were often contradictory, especially concerning germination (Table A3).

All the species were initially grown in a greenhouse (table heating at ca. 20 °C) and then transplanted to three cold frames without glass covers and filled with a compost–soil mixture (each plot: 1.40 m × 15 m; in total: 63 m^2^). From *Persicaria tinctoria*, stems with leaves were harvested before flowering. From the *Indigofera* species, stems with leaves were harvested during the appearance of inflorescence/flowering/flowering and the first green pod stage. Harvested plants were transported in plastic bags to the laboratory to avoid evaporation and wilting before further processing (Table A3).

#### 4.1.3. Standardised Indigo Extraction Methods

##### **MME (Mobile Mini Extraction)** 

This method is based on previous studies [33,55,68,83,84,85] and was adapted as a transportable, standardised extraction method for field research. To avoid pigment loss during decanting, the whole extraction liquid was filtered until the filtrate was clear and no longer showed any traces of blue pigment.

MME procedure: Extractions were performed in three replications simultaneously with plant material from the same harvest (mixed sample from various individual plants). Immediately after the harvesting, 500 g of fresh plant material was rinsed with water and distributed equally into two mesh bags. The plant material was soaked for 3 h in three litres of water at 45 °C (the plants were kept submersed by two tightly closed glasses filled with water and placed on top of the mesh bags). The mesh bags with the plant material were then taken out and squeezed to avoid loss of the soaking liquid. The liquid was aerated for 30 min with a fish tank pump and then left for 20 h to allow the pigment to settle at the bottom. The whole liquid was then repeatedly filtered off through white cotton fabric until the filtrate was clear.

Modified MME of *Indigofera arrecta*: Thick stems were not removed, and the plant material was soaked until a thick copperish skin became visible on the surface, but the smell was still a “good indigo smell”, as described by P.-P. Darrac [7], before the typical putrid smell of excessive fermentation was noticeable (MME.69–71, unknown provenance: soaking duration, 5 h).

Modified MME of *Wrightia laevis*: As the standardised soaking duration of 3 h would not have yielded any indigo, the soaking duration was extended until a copperish film became visible on the surface of the liquid (MME.13–15, tree 1: soaking duration, 6 h; MME.16–18, tree 2: 9 h).

##### **sLPE (Simulated Local Practice Extraction)**

This extraction method was carried out only with *Persicaria tinctoria* and *Indigofera arrecta*. It was developed to simulate traditional practices of extraction at ambient temperature and the addition of lime before aeration. The method builds on previous experiments of the authors with *Persicaria tinctoria* [84] and *Strobilanthes cusia* [85].

sLPE procedure: Extractions were performed in three replications and carried out simultaneously with plant material from the same harvest as used for the MMEs. Immediately after the harvesting, 500 g of fresh plant material was rinsed with water and distributed equally into two mesh bags. The mesh bags with the plant material were placed around a perforated pipe in the centre of a beaker. Three litres of water at 20 °C were added, and the beaker was kept in an incubator at 30 °C for 48 h (the plants were kept submersed by two tightly closed glasses filled with water and placed on top of the mesh bags). The mesh bags with the plant material were then taken out and squeezed to avoid loss of the soaking liquid. Then, 5 g of hydrated lime (Ca(OH)_2_) mixed in a small amount of water was added to the soaking liquid (a constant amount of lime rather than a standardised pH value was used in order to avoid influencing the indigo yield with the weight of lime added). The liquid was aerated for 30 min with a fish tank pump and afterwards left for 20 h to allow the pigment to settle at the bottom. The whole liquid was then repeatedly filtered off through white cotton fabric until the filtrate was clear.

Modified sLPE of *Indigofera arrecta*: Thick stems were not removed, and the plant material was soaked until a thick copperish skin became visible on the surface and the smell was still a “good indigo smell”, before the typical putrid smell of an excessive fermentation was noticeable (soaking duration: 28 h instead of 48 h). Instead of 5 g of lime, 7 g of lime were used to adjust to a pH of 11 (sLPE.28–30, unknown provenance).

### 4.2. Indigo Samples from Local Practices in China, Indonesia and India

Indigo samples were bought from traditional local indigo producers during field research in China in 2019 and from indigo producers in India and Indonesia in 2021, with their permission to analyse and publish results (prior informed consent).

LPE (local practice extraction) samples were extracted by local people during field research in China at the same time and using the same plant material of *Indigofera tinctoria*, *Strobilanthes cusia* and *Wrightia laevis* from the same harvest as the corresponding MMEs. The traditional extraction method uses lime during the aeration of the soaking liquid. In principle, the method is very similar regardless of the species on which the extraction is being performed; its variations mainly concern the duration of the processing steps (Table 5 and Table S2).

ADD samples (additional samples from local practice) were bought during field research in China and taken from indigo already available in the villages from previous harvests (species according to the interviewees, traditional extraction method). Samples were also obtained from project partners in India (Avani) and Indonesia (Threads of Life) (extraction methods with and without using lime, Table 5 and Table S2).

### 4.3. Indigo Sample Preparation

The cotton filters with the indigo pigment were first air-dried, and then dried in the drying cabinet according to the standardised procedure at 60 °C until weight was consistent. The dry indigo was scraped off the filter and kept protected from light (using brown glass vials in a cardboard box). The samples received from *Avani*, India (traded as powder) and from *Threads of Life*, Indonesia (normally traded as paste, but air-dried and roughly ground for shipment to Vienna) were not dried in a drying cabinet to avoid cross-contamination. All the samples were ground with a *Qiagen Tissue Lyser 2* (Venlo, The Netherlands), with the samples in 2 mL microcentrifuge safe-lock tubes (Eppendorf, Hamburg, Germany) with four zirconium oxide grinding balls (3 mm) per tube (frequency: 30 Hz, grinding duration: 2:30 min).

### 4.4. Determination of Indigotin Content, Indigo Yield and Indigotin Yield

#### 4.4.1. Spectrophotometric Analysis of Indigotin Content

The spectrophotometric method of Bechtold et al. [54,86] measures the content of *leuco*-indigotin in an aqueous alkaline solution with Fe(II)-triethanolamine as the reducing agent. The method was adapted to a 96-well microplate reader format, and measurements were performed in non-treated 96-well microplates (BRANDplates^®^ pureGrade™, Wertheim, Germany) using a plate reader (Infinite M200Pro, Tecan, Männedorf, Switzerland).

In a volumetric flask (100 mL), ground indigo (5 mg) was reduced with 20 mL of solution A (33 g/L NaOH, 333 g/L triethanolamine, 50 g/L FeSO_4_·7H_2_O, 0.8 g/L citric acid) and filled up to 100 mL with solution B (10 g/L NaOH, 50 g/L triethanolamine, 5 g/L FeSO_4_·7H_2_O). The volumetric flask with a stopper was kept in a water bath at 40 °C for 60 min (shaking every 15 min, three times in total). For each indigo sample, three sample preparations in duplicate (150 µL per well) were analysed on different days at 40 °C (microplate with lid, waiting for 2 min after insertion to avoid steaming up), and with absorbance at a wavelength of 410 nm. Per analysis run, six standards (0–50 ppm) and a maximum of ten indigo samples were analysed to ensure the completion of the analyses within the given time frame for sample preparation stability (2 h). Synthetic indigo (Acros Organics, ≥94%) was used as the standard for calibration.

Intra-day and inter-day variations for the MME method were 1.4% and 5.42%, respectively, and for the sLPE method, 5.4% and 16.4%, respectively. The regression coefficient was 0.9945.

#### 4.4.2. Calculations

Content of indigotin: The indigotin content in the indigo pigment (weight percent) was calculated from the single concentration measured and the indigo sample weight.

Indigo extraction yield: As the indigo pigment could not be scraped off the filter without losses (indigo remaining on the filter), the indigo yield in g (dry) per kg fresh plant material was calculated by determining the dry weight of the filter with indigo and subtracting the filter weight (both drying at 60 °C, until weight consistency).

Indigotin extraction yield: The indigotin yield in g (dry) per kg of fresh plant material was calculated based on the indigo extraction yield and the indigotin content of the sample.

### 4.5. Determination of Indigo Sample Colour

#### 4.5.1. Visual Classification of Colours

All standardised ground indigo samples were visually classified in the same light conditions at the same time into groups of similar colours, described as follows: (1) dark blue/violet, (2) dark blue/black, (3) dark blue, (4) mid blue, (5) light blue, (6) grey blue, (7) greenish blue and (8) green.

#### 4.5.2. L*a*b* Colour Measurement

With the L*a* b* colour coordinates, the position of a colour in the three-dimensional *CIELAB Colour Space* can be precisely defined. L* is the lightness axis reaching from black (L* = 0) to white (L* = 100); a* and b* are two complementary colour axes: the a* axis points toward red (positive a*) and green (negative a*), and the b* axis, toward yellow (positive b*) and blue (negative b*) [87].

L*a*b* values were determined with the *Nix Quality Control Color Sensor (Nix QC)* and the *Nix Toolkit App* both from Nix Sensor Ltd. (Hamilton, ON, Canada). To avoid contamination, each sample was measured between two cover glasses of microscope slides.

### 4.6. Statistical Data Analysis

The statistical data analyses were carried out with R [88] under RStudio [89] using the packages corrplot [90], factoextra [91] and FactoMineR [92].

## 5. Conclusions

This study provides an evaluation of six indigo-yielding species (*Indigofera arrecta*, *I. tinctoria*, *I. suffruticosa*, *Persicaria tinctoria*, *Strobilanthes cusia* and *Wrightia laevis*) with regard to indigo quality (content of indigotin) as well as indigo and indigotin extraction yields. A comparison of indigo-yielding species requires a differentiated approach: a high indigotin content in the indigo can be achieved with several species (the maxima were 55% with *Indigofera arrecta*, Austria, and 56% with *Strobilanthes cusia*, China). Even with *Wrightia laevis*, a species known for low quality, a similar indigotin content to that from the *Indigofera* species can be achieved when the extraction methods are optimised. The indigotin yield per kg of plant material, however, was clearly highest with *Strobilanthes cusia* (2.6 g/kg) compared with 0.5–0.7 g/kg extracted in China from the other species (without statistically significant difference among *Indigofera tinctoria*, *I. suffruticosa* and *Wrightia laevis*) and the maxima achieved in Austria (0.6 and 0.8 g/kg achieved with *Persicaria tinctoria* and *Indigofera arrecta*, respectively).

*Indigofera arrecta*, a species once highly valued, could become an interesting alternative species again due to its high indigo quality and indigotin extraction yield.

Indigo colour, once (and, in some traditional contexts, still) used as an indicator of quality, could still serve this purpose in a broader context as well, as demonstrated by the first correlation and regression analysis. As small, inexpensive, and easy-to-handle L*a*b* measurement tools are now available, this method could become a quick quality control tool both in scientific field research and in practice as well, without the need for costly laboratory facilities.

## Figures and Tables

**Table 1:** Comparison of the MME and sLPE methods with both *Persicaria tinctoria* and *Indigofera arrecta* (mean values of three and two provenances, respectively); comparisons of MME and mod.MME and of sLPE and mod.sLPE with one provenance of *Indigofera arrecta* (mean values of extraction replications). *t*-test; for L*a*b*values, the aggregated data set was used.

Species	Parameter		Extraction Methods		Statistical Significance
			MME	sLPE	
*Persicaria tinctoria* (“Senbon”, “Maruba” and “Kojyoko”)	Indigotin in indigo (%)		43.84	4.50	***
	Indigo extraction yield (g/kg fresh weight)		1.34	15.33	***
	Indigotin extraction yield (g/kg fresh weight)		0.61	0.69	*
	CIELAB colour coordinates:	L*	9.76	32.71	***
		a*	2.75	−4.14	***
		b*	−10.25	−11.97	***
*Indigofera arrecta* (Burkina Faso and unknown provenance)	Indigotin in indigo (%)		53.48	6.23	***
	Indigo extraction yield (g/kg fresh weight)		1.38	18.54	***
	Indigotin extraction yield (g/kg fresh weight)		0.75	1.17	n.s.
	CIELAB colour coordinates:	L*	11.69	32.78	***
		a*	3.40	−6.27	***
		b*	−9.81	1.23	*
*Indigofera arrecta* (unknown provenance)			**MME**	**mod.MME**	
	Indigotin in indigo (%)		58.13	58.03	n.s.
	Indigo extraction yield (g/kg fresh weight)		1.44	2.17	***
	Indigotin extraction yield (g/kg fresh weight)		0.84	1.25	***
	CIELAB colour coordinates:	L*	12.12	10.15	***
		a*	3.23	2.44	***
		b*	−8.72	−8.52	n.s.
			**sLPE**	**mod.sLPE**	
	Indigotin in indigo (%)		8.26	6.37	***
	Indigo extraction yield (g/kg fresh weight)		19.28	22.59	***
	Indigotin extraction yield (g/kg fresh weight)		1.59	1.44	n.s.
	CIELAB colour coordinates:	L*	25.13	26.24	**
		a*	−5.94	−3.80	***
		b*	−3.87	−9.42	**

Significance levels: *: *p* < 0.05 > 0.01, **: *p* < 0.01 > 0.001 and ***: *p* < 0.001; n.s.: not significant, *p* > 0.05.

**Table 2:** Examples representing the eight visually determined colour classes (including sample information and L*a*b* values of the samples shown in the picture) and the number of samples per colour class.

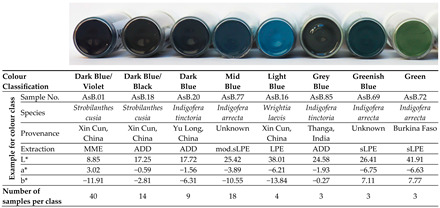

**Table 3:** Multivariate linear regression of indigotin content on L*a*b* values. The aggregated data set was used.

	Estimate	Std. Error	t Value	Pr(>|t|)	
(Intercept)	50.8313	13.0766	3.887	0.00026	***
L*	−2.1510	0.7518	−2.861	0.00580	**
a*	9.7976	2.3863	4.106	0.00012	***
b*	0.4692	1.1977	0.392	0.69661	
L*:a*	−0.4253	0.1164	−3.654	0.00054	***
L*:b*	−0.0398	0.0691	−0.577	0.56576	
a*:b*	−0.2023	0.2308	−0.877	0.38418	
L*:a*:b*	0.0044	0.0114	0.391	0.69728	

** and *** indicate significance at the 95% and 99% levels, respectively.

**Table 5:** Overview of the indigo sample set.

	Species, Provenances (of Seeds ^1^, Harvested Plant Material or Indigo Samples) and Code for Extraction Methods (Number of Samples in Brackets)					
	*Indigofera tinctoria*	*Indigofera suffruticosa*	*Indigofera arrecta*	*Persicaria tinctoria*	*Strobilanthes cusia*	*Wrightia laevis*
**Standardised extraction in Austria** (own cultivation in Vienna)	Burkina Faso: MME (3) ^2^ Maldives: MME (3)	Venezuela: MME (3) ^2^ Ecuador: MME (3) Brazil: MME (3)	Mali: MME (3) Burkina Faso: MME (3) + sLPE (3) unknown provenance: MME (3) + mod.MME (3) ^2^ sLPE (3) + mod.sLPE (3) ^2^	“Senbon”: MME (3) + sLPE (3) “Kojyoko”: MME (3) + sLPE (3) “Maruba”: MME (3) + sLPE (3)	-	-
**Standardised extraction in China** (local practice cultivation in three villages in Hainan)	Yu Long: MME (3)	Yu Dao: MME (3)	-	-	Xin Cun: MME (3)	Xin Cun: Tree 1: MME (3) ^3^ Tree 2: MME (3) ^4^
**Total samples**	9	12	21	18	3	6
**Local practice extraction in China** (three villages in Hainan)	Yu Dao: extraction with lime, LPE (1) Yu Long: extraction with lime, LPE (1) + ADD (4)	-	-	-	Xin Cun: extraction with lime, LPE (1) + ADD (2)	Xin Cun: extraction with lime, LPE (1)
**Local practice extraction in** **India** (six villages in Uttarakhand)	Simalta: 1st extraction without lime + 2nd extraction with lime, ADD (2) Digoli: 1st extraction without lime + 2nd extraction with lime, ADD (2) Thanga: 1st extraction without lime + 2nd extraction with lime, ADD (2) Chankana: 1st extraction without lime + 2nd extraction with lime, ADD (2)	-	-	-	Simalta: extraction without lime, ADD (1) Tripuradevi: extraction without lime, ADD (1) Dharamghar: 1st extraction without lime + 2nd extraction with lime, ADD (2)	-
**Local practice extraction in Indonesia** (two locations in Java and Bali)	-	-	-	-	Temangung/Java: extraction with lime, ADD (1) Ubud/Bali: extraction without lime, ADD (2)	-
**Total samples**	14	0	0	0	10	1
**TOTAL SAMPLES** (standardised + local practice)	23	15	21	18	13	7

Abbreviations: MME = mobile mini extraction, sLPE = simulated local practice extraction, mod. = modified method, LPE = local practice extraction (same plant material as used for MME), ADD = additional samples from local practice extraction. (^1^) Seed sources and accession numbers are provided in Table A2; (^2^) thick stems not removed from the extracted plant material; (^3^) extended extraction duration: 6 h (instead of 3 h), (^4^) extended extraction duration: 9 h (instead of 3 h).

## Data Availability

Data are contained within the article and supplementary files.

## Supplementary Materials

The following supporting information can be downloaded at: https://www.mdpi.com/article/10.3390/plants13070918/s1, Raw data including the results of the indigotin analysis, yield calculations, colour classification and L*a*b* data: Table S1: Standardised extractions (MME, sLPE and modifications); Table S2: Local practice extractions (LPE, ADD); Table S3: Validation. Scans of voucher specimens.

## Appendix A

**Table A1:** Indigo terminology in the literature.

Terms/Context to Describe the Plant Extract	Terms/Context to Describe the Valuable Component for Dyeing
“crude dyestuff” [54] “crude indigo dye” [55] “crude indigo” [49,50,59,79] “indigo product” [32,78] “indigo paste” [77] “indigo powder” [79] “commercial indigo” [50] “indigo dye” [58,59,64] “indigo colour”, “indigo blue” [58] “dye”, “dye recovery” [77] “blue dye powder”, “blue colorant powder” [80] “woad powder” [82] “indigo” [25,30,51,63]	“indigo content” [49,54,55,64,65] “indigo percentage” [65] “indigo purity” [32,50] “pure indigo” [49,55,79] “indigo” [30,32,50,59,64,65,66,77,78,79,80,81] “colorant purity”, “dye purity” [80] “indigo dye concentration” [82] “indigotin” mentioned as a synonym for “indigo” [66,77,79,81] “indigotin” [25,51,63]

**Table A2:** Seed provenances.

Species	Seed Source	Country of Origin, Collection Year	Accession Number/ Name of Provenance
*Indigofera tinctoria*	Millennium Seed Bank, Royal Botanic Gardens, Kew, UK	Burkina Faso, 1998	125549
	Millennium Seed Bank, Royal Botanic Gardens, Kew, UK	Maldives, 1986	68068
*Indigofera suffruticosa*	Millennium Seed Bank, Royal Botanic Gardens, Kew, UK	Brazil, 1983	47896
	Millennium Seed Bank, Royal Botanic Gardens, Kew, UK	Ecuador, 1980	17479
	Millennium Seed Bank, Royal Botanic Gardens, Kew, UK	Venezuela, 1994	103897
*Indigofera* *arrecta*	Bonn University Botanic Gardens, Bonn, Germany	unknown	xx-0-BONN-19246
	Millennium Seed Bank, Royal Botanic Gardens, Kew, UK	Burkina Faso, 2007	444266
	Millennium Seed Bank, Royal Botanic Gardens, Kew, UK	Mali, 2014	820888
*Persicaria tinctoria*	Bailiwick Blue, Guernsey, UK	unknown	“Maruba”
	Bailiwick Blue, Guernsey, UK	unknown	“Senbon”
	Bailiwick Blue, Guernsey, UK	unknown	“Kojyoko”

**Table A3:** Cultivation of indigo-yielding species in Vienna, Austria in 2021.

	*Persicaria tinctoria*	*Indigofera tinctoria*	*Indigofera suffruticosa*	*Indigofera arrecta*
**Cultivation in the greenhouse:**				
Seed treatment	None	All *Indigofera* sp.: scarification to break seed dormancy		
Sowing in seedling trays	30/04	06/05	06/05	06/05
Seeds per pot	3	1	1	1
Potting	21/05 and 31/05	07/06	07/06	07/06
Pruning	None	All *Indigofera* sp.: pruning of main shoot to enhance ramification and increase biomass yield, because only a few seeds were available per provenance		
Watering	All species: as required			
Treatment against *Sciaridae*	All species: yellow sticky traps and beneficial nematodes			
**Cultivation in the field:**				
Transplanting	17/06	28/06	28/06	22 + 28/06
Spacing	30 cm × 28 cm	30 cm × 20 cm	30 cm × 20 cm	30 cm × 20 cm
Application of horn shavings	All species: 17/07, ca. 25 kg/63 m^2^			
Weeding	All species: 22/06, 05/07, 12/07, 20/07, 12/08			
Watering	All species: as required			
Harvested plant material	Stems with leaves *	Stems with leaves * + flowers/green pods	Stems with leaves * + flowers	Stems with leaves * + inflorescences/flowers/green pods

(*) If the leaves on the lower part of the stems had already dropped, only the upper part with leaves still attached was harvested. *Indigofera* species: apart from MME.45–47, MME.48–50, MME.69–71 and sLPE.28–30, very thick main stems were removed and only the thinner side branches were used for extraction.

**Table A4:** Comparison of the L*a*b* values of indigo from different species, per country. All samples extracted using the MME method (Austria: mean values of all provenances per species, China: one provenance per species; therefore, mean values of extraction replications). Values with the same letter do not differ significantly within a country (ANOVA, *p* = 0.05, aggregated data set).

Country	Species	Colour Coordinates					
		L*		a*		b*	
Austria	*Indigofera arrecta*	11.37	bc	3.39	d	−9.76	gh
	*Indigofera suffruticosa*	18.69	a	−0.94	f	−7.49	g
	*Indigofera tinctoria*	13.06	b	1.57	e	−8.91	gh
	*Persicaria tinctoria*	9.76	c	2.75	d	−10.25	h
China	*Indigofera suffruticosa*	14.09	s	0.71	w	5.89	x
	*Indigofera tinctoria*	12.75	st	0.49	w	−7.17	xy
	*Strobilanthes cusia*	9.28	tu	2.77	v	−12.06	z
	*Wrightia laevis* (tree 1) ^1^	8.49	u	2.07	v	−12.96	z
	*Wrightia laevis* (tree 2) ^2^	11.9	stu	0.06	w	−10.83	yz

(^1^) Extraction duration extended to 6 h. (^2^) Extraction duration extended to 9 h. Mean value of two replications (the sample from one replication was too small for L*a*b* measurement).

**Table A5:** Comparison of provenances within a species: L*a*b* values of indigo samples extracted with the MME method, cultivated in Austria (mean values per provenances). Values with the same letter do not differ significantly within a species (ANOVA, *p* = 0.05, aggregated data set).

Species	Provenance	Colour Coordinates					
		L*		a*		b*	
*Indigofera arrecta*	Burkina Faso 1	11.27	a	3.57	b	−10.89	d
	Mali	10.72	a	3.36	b	−9.66	cd
	unknown	12.12	a	3.23	b	−8.72	c
*Indigofera suffruticosa*	Brazil	21.39	e	−1.22	f	−4.89	g
	Ecuador	17.93	e	−0.56	f	−7.99	g
	Venezuela ^1^	16.76	e	−1.06	f	−9.58	g
*Indigofera tinctoria*	Burkina Faso 2 ^1^	13.56	h	1.15	j	−8.00	k
	Maldives	12.56	h	2.00	i	−9.82	k
*Persicaria tinctoria*	“Kojyoko”	9.20	m	2.80	n	−10.26	o
	“Maruba”	9.00	m	3.17	n	−9.97	o
	“Senbon”	11.07	l	2.29	n	−10.52	o

(^1^) Thick stems not removed from harvested plant material.
