# Supplementary material for: “Purplish Blue” or “Greenish Grey”? Indigo Qualities and Extraction Yields from Six Species
Source: Plants (Basel). 2024 Mar 22;13(7):918. doi: 10.3390/plants13070918 (PMC11013892; doi:10.3390/plants13070918)
Supplement: Supplementary file 1 [file plants-13-00918-s001.zip › Table S1.pdf]

Table S1. Raw data of samples from standardised extractions (MME, sLPE and modifications)

AsB-No.	Sample code	Species	Provenance	Cultivation	Type of extraction	Extraction replication	Sample preparation	Duplicates	Raw data (= Absorp-tion)	Single conc. (µg/ml)	Dilution factor	Indigo weight (g)	Indigotin (%)	Indigo extraction yield (g/kg fresh plant material)	Indigotin extraction yield (g/kg fresh plant material)	Colour classification of ground indigo:<br>dark blue / violet<br>dark blue / black<br>dark blue<br>mid blue<br>light blue<br>grey blue<br>greenish blue<br>green	L*	a*	b*
**AsB.33**	**MME.45**	Ind.tinc.	Burkina Faso	Austria	MME	1	1	1	0,593	9,2626	1	0,00501	18,49	2,84	0,53	dark blue / black	14,64	0,79	-5,93
**AsB.33**	**MME.45**	Ind.tinc.	Burkina Faso	Austria	MME	1	1	2	0,558	8,3922	1	0,00501	16,75	2,84	0,48	dark blue / black	14,64	0,79	-5,93
**AsB.33**	**MME.45**	Ind.tinc.	Burkina Faso	Austria	MME	1	2	1	0,6503	9,0094	1	0,00519	17,36	2,84	0,49	dark blue / black	14,64	0,79	-5,93
**AsB.33**	**MME.45**	Ind.tinc.	Burkina Faso	Austria	MME	1	2	2	0,6549	9,1827	1	0,00519	17,69	2,84	0,50	dark blue / black	14,64	0,79	-5,93
**AsB.33**	**MME.45**	Ind.tinc.	Burkina Faso	Austria	MME	1	3	1	0,5901	9,0402	1	0,00515	17,55	2,84	0,50	dark blue / black	14,64	0,79	-5,93
**AsB.33**	**MME.45**	Ind.tinc.	Burkina Faso	Austria	MME	1	3	2	0,5884	8,9961	1	0,00515	17,47	2,84	0,50	dark blue / black	14,64	0,79	-5,93
**AsB.34**	**MME.46**	Ind.tinc.	Burkina Faso	Austria	MME	2	1	1	0,5997	9,4292	1	0,00527	17,89	1,62	0,29	dark blue / violet	13,2	1,5	-8,77
**AsB.34**	**MME.46**	Ind.tinc.	Burkina Faso	Austria	MME	2	1	2	0,6266	10,144	1	0,00527	19,25	1,62	0,31	dark blue / violet	13,2	1,5	-8,77
**AsB.34**	**MME.46**	Ind.tinc.	Burkina Faso	Austria	MME	2	2	1	0,678	10,048	1	0,00524	19,18	1,62	0,31	dark blue / violet	13,2	1,5	-8,77
**AsB.34**	**MME.46**	Ind.tinc.	Burkina Faso	Austria	MME	2	2	2	0,677	10,014	1	0,00524	19,11	1,62	0,31	dark blue / violet	13,2	1,5	-8,77
**AsB.34**	**MME.46**	Ind.tinc.	Burkina Faso	Austria	MME	2	3	1	0,5996	9,2866	1	0,00510	18,21	1,62	0,29	dark blue / violet	13,2	1,5	-8,77
**AsB.34**	**MME.46**	Ind.tinc.	Burkina Faso	Austria	MME	2	3	2	0,6007	9,3152	1	0,00510	18,27	1,62	0,30	dark blue / violet	13,2	1,5	-8,77
**AsB.35**	**MME.47**	Ind.tinc.	Burkina Faso	Austria	MME	3	1	1	0,5642	8,5464	1	0,00503	16,99	1,66	0,28	dark blue / violet	12,85	1,15	-9,29
**AsB.35**	**MME.47**	Ind.tinc.	Burkina Faso	Austria	MME	3	1	2	0,5803	8,9468	1	0,00503	17,79	1,66	0,30	dark blue / violet	12,85	1,15	-9,29
**AsB.35**	**MME.47**	Ind.tinc.	Burkina Faso	Austria	MME	3	2	1	0,6711	9,7928	1	0,00533	18,37	1,66	0,30	dark blue / violet	12,85	1,15	-9,29
**AsB.35**	**MME.47**	Ind.tinc.	Burkina Faso	Austria	MME	3	2	2	0,7048	10,967	1	0,00533	20,58	1,66	0,34	dark blue / violet	12,85	1,15	-9,29
**AsB.35**	**MME.47**	Ind.tinc.	Burkina Faso	Austria	MME	3	3	1	0,5806	8,7938	1	0,00536	16,41	1,66	0,27	dark blue / violet	12,85	1,15	-9,29
**AsB.35**	**MME.47**	Ind.tinc.	Burkina Faso	Austria	MME	3	3	2	0,5909	9,061	1	0,00536	16,90	1,66	0,28	dark blue / violet	12,85	1,15	-9,29
**AsB.39**	**MME.51**	Ind.tinc.	Maldives	Austria	MME	1	1	1	0,6505	10,103	1	0,00506	19,97	1,72	0,34	dark blue / violet	12,73	1,95	-10,78
**AsB.39**	**MME.51**	Ind.tinc.	Maldives	Austria	MME	1	1	2	0,6389	9,8167	1	0,00506	19,40	1,72	0,33	dark blue / violet	12,73	1,95	-10,78
**AsB.39**	**MME.51**	Ind.tinc.	Maldives	Austria	MME	1	2	1	0,7342	11,974	1	0,00528	22,68	1,72	0,39	dark blue / violet	12,73	1,95	-10,78
**AsB.39**	**MME.51**	Ind.tinc.	Maldives	Austria	MME	1	2	2	0,7074	11,056	1	0,00528	20,94	1,72	0,36	dark blue / violet	12,73	1,95	-10,78
**AsB.39**	**MME.51**	Ind.tinc.	Maldives	Austria	MME	1	3	1	0,6455	9,7796	1	0,00509	19,21	1,72	0,33	dark blue / violet	12,73	1,95	-10,78
**AsB.39**	**MME.51**	Ind.tinc.	Maldives	Austria	MME	1	3	2	0,6534	9,9699	1	0,00509	19,59	1,72	0,34	dark blue / violet	12,73	1,95	-10,78
**AsB.40**	**MME.52**	Ind.tinc.	Maldives	Austria	MME	2	1	1	0,7734	13,203	1	0,00530	24,91	0,68	0,17	dark blue / violet	12,85	1,65	-8,96
**AsB.40**	**MME.52**	Ind.tinc.	Maldives	Austria	MME	2	1	2	0,7996	13,864	1	0,00530	26,16	0,68	0,18	dark blue / violet	12,85	1,65	-8,96
**AsB.40**	**MME.52**	Ind.tinc.	Maldives	Austria	MME	2	2	1	0,7744	13,352	1	0,00524	25,48	0,68	0,17	dark blue / violet	12,85	1,65	-8,96
**AsB.40**	**MME.52**	Ind.tinc.	Maldives	Austria	MME	2	2	2	0,8102	14,579	1	0,00524	27,82	0,68	0,19	dark blue / violet	12,85	1,65	-8,96
**AsB.40**	**MME.52**	Ind.tinc.	Maldives	Austria	MME	2	3	1	0,8001	13,551	1	0,00512	26,47	0,68	0,18	dark blue / violet	12,85	1,65	-8,96
**AsB.40**	**MME.52**	Ind.tinc.	Maldives	Austria	MME	2	3	2	0,7881	13,258	1	0,00512	25,89	0,68	0,18	dark blue / violet	12,85	1,65	-8,96
**AsB.41**	**MME.53**	Ind.tinc.	Maldives	Austria	MME	3	1	1	1,0129	19,243	1	0,00523	36,79	0,92	0,34	dark blue / violet	12,1	2,4	-9,71
**AsB.41**	**MME.53**	Ind.tinc.	Maldives	Austria	MME	3	1	2	0,9903	18,673	1	0,00523	35,70	0,92	0,33	dark blue / violet	12,1	2,4	-9,71
**AsB.41**	**MME.53**	Ind.tinc.	Maldives	Austria	MME	3	2	1	0,9537	17,955	1	0,00508	35,34	0,92	0,33	dark blue / violet	12,1	2,4	-9,71
**AsB.41**	**MME.53**	Ind.tinc.	Maldives	Austria	MME	3	2	2	0,9833	18,732	1	0,00508	36,87	0,92	0,34	dark blue / violet	12,1	2,4	-9,71
**AsB.41**	**MME.53**	Ind.tinc.	Maldives	Austria	MME	3	3	1	1,0299	19,162	1	0,00510	37,57	0,92	0,35	dark blue / violet	12,1	2,4	-9,71
**AsB.41**	**MME.53**	Ind.tinc.	Maldives	Austria	MME	3	3	2	1,0348	19,282	1	0,00510	37,81	0,92	0,35	dark blue / violet	12,1	2,4	-9,71
**AsB.12**	**MME.24**	Ind.tinc.	Yu Long	China	MME	1	1	1	0,8136	15,186	1	0,00515	29,49	2,42	0,71	dark blue / black	11,03	1,04	-8,32
**AsB.12**	**MME.24**	Ind.tinc.	Yu Long	China	MME	1	1	2	0,8108	15,118	1	0,00515	29,36	2,42	0,71	dark blue / black	11,03	1,04	-8,32
**AsB.12**	**MME.24**	Ind.tinc.	Yu Long	China	MME	1	2	1	0,8678	16,61	1	0,00528	31,46	2,42	0,76	dark blue / black	11,03	1,04	-8,32
**AsB.12**	**MME.24**	Ind.tinc.	Yu Long	China	MME	1	2	2	0,8412	15,87	1	0,00528	30,06	2,42	0,73	dark blue / black	11,03	1,04	-8,32
**AsB.12**	**MME.24**	Ind.tinc.	Yu Long	China	MME	1	3	1	0,8468	16,605	1	0,00519	31,99	2,42	0,77	dark blue / black	11,03	1,04	-8,32
**AsB.12**	**MME.24**	Ind.tinc.	Yu Long	China	MME	1	3	2	0,8198	15,9	1	0,00519	30,64	2,42	0,74	dark blue / black	11,03	1,04	-8,32
**AsB.13**	**MME.25**	Ind.tinc.	Yu Long	China	MME	2	1	1	0,6691	11,709	1	0,00533	21,97	2,22	0,49	dark blue / black	13,33	0,5	-7,07
**AsB.13**	**MME.25**	Ind.tinc.	Yu Long	China	MME	2	1	2	0,6675	11,671	1	0,00533	21,90	2,22	0,49	dark blue / black	13,33	0,5	-7,07
**AsB.13**	**MME.25**	Ind.tinc.	Yu Long	China	MME	2	2	1	0,6469	10,46	1	0,00514	20,35	2,22	0,45	dark blue / black	13,33	0,5	-7,07
**AsB.13**	**MME.25**	Ind.tinc.	Yu Long	China	MME	2	2	2	0,6561	10,716	1	0,00514	20,85	2,22	0,46	dark blue / black	13,33	0,5	-7,07
**AsB.13**	**MME.25**	Ind.tinc.	Yu Long	China	MME	2	3	1	0,6339	10,42	1	0,00512	20,35	2,22	0,45	dark blue / black	13,33	0,5	-7,07
**AsB.13**	**MME.25**	Ind.tinc.	Yu Long	China	MME	2	3	2	0,6441	10,7	1	0,00512	20,90	2,22	0,46	dark blue / black	13,33	0,5	-7,07
**AsB.14**	**MME.26**	Ind.tinc.	Yu Long	China	MME	3	1	1	0,6047	10,16	1	0,00529	19,21	1,98	0,38	dark blue / black	13,89	-0,08	-6,11
**AsB.14**	**MME.26**	Ind.tinc.	Yu Long	China	MME	3	1	2	0,6019	10,093	1	0,00529	19,08	1,98	0,38	dark blue / black	13,89	-0,08	-6,11

**AsB.14**	**MME.26**	Ind.tinc.	Yu Long	China	MME	3	2	1	0,5545	9,2193	1	0,00519	17,76	1,98	0,35	dark blue / black	13,89	-0,08	-6,11
**AsB.14**	**MME.26**	Ind.tinc.	Yu Long	China	MME	3	2	2	0,5699	9,6756	1	0,00519	18,64	1,98	0,37	dark blue / black	13,89	-0,08	-6,11
**AsB.14**	**MME.26**	Ind.tinc.	Yu Long	China	MME	3	3	1	0,5695	8,6821	1	0,00510	17,02	1,98	0,34	dark blue / black	13,89	-0,08	-6,11
**AsB.14**	**MME.26**	Ind.tinc.	Yu Long	China	MME	3	3	2	0,5674	8,6257	1	0,00510	16,91	1,98	0,33	dark blue / black	13,89	-0,08	-6,11
**AsB.36**	**MME.48**	Ind.suff.	Venezuela	Austria	MME	1	1	1	0,5485	8,1559	1	0,00509	16,02	2,32	0,37	dark blue / violet	14,98	0,64	-7,42
**AsB.36**	**MME.48**	Ind.suff.	Venezuela	Austria	MME	1	1	2	0,5346	7,8102	1	0,00509	15,34	2,32	0,36	dark blue / violet	14,98	0,64	-7,42
**AsB.36**	**MME.48**	Ind.suff.	Venezuela	Austria	MME	1	2	1	0,6124	7,5819	1	0,00518	14,64	2,32	0,34	dark blue / violet	14,98	0,64	-7,42
**AsB.36**	**MME.48**	Ind.suff.	Venezuela	Austria	MME	1	2	2	0,6023	7,2015	1	0,00518	13,90	2,32	0,32	dark blue / violet	14,98	0,64	-7,42
**AsB.36**	**MME.48**	Ind.suff.	Venezuela	Austria	MME	1	3	1	0,5276	7,4189	1	0,00503	14,75	2,32	0,34	dark blue / violet	14,98	0,64	-7,42
**AsB.36**	**MME.48**	Ind.suff.	Venezuela	Austria	MME	1	3	2	0,5321	7,5357	1	0,00503	14,98	2,32	0,35	dark blue / violet	14,98	0,64	-7,42
**AsB.37**	**MME.49**	Ind.suff.	Venezuela	Austria	MME	2	1	1	0,5134	6,749	1	0,00536	12,59	1,40	0,18	dark blue	17,24	-1,79	-11,15
**AsB.37**	**MME.49**	Ind.suff.	Venezuela	Austria	MME	2	1	2	0,5156	6,8027	1	0,00536	12,69	1,40	0,18	dark blue	17,24	-1,79	-11,15
**AsB.37**	**MME.49**	Ind.suff.	Venezuela	Austria	MME	2	2	1	0,583	6,4746	1	0,00500	12,95	1,40	0,18	dark blue	17,24	-1,79	-11,15
**AsB.37**	**MME.49**	Ind.suff.	Venezuela	Austria	MME	2	2	2	0,5976	7,0245	1	0,00500	14,05	1,40	0,20	dark blue	17,24	-1,79	-11,15
**AsB.37**	**MME.49**	Ind.suff.	Venezuela	Austria	MME	2	3	1	0,514	6,6117	1	0,00521	12,69	1,40	0,18	dark blue	17,24	-1,79	-11,15
**AsB.37**	**MME.49**	Ind.suff.	Venezuela	Austria	MME	2	3	2	0,5091	6,4936	1	0,00521	12,46	1,40	0,17	dark blue	17,24	-1,79	-11,15
**AsB.38**	**MME.50**	Ind.suff.	Venezuela	Austria	MME	3	1	1	0,5065	6,5803	1	0,00509	12,93	1,22	0,16	dark blue	18,08	-2,02	-10,18
**AsB.38**	**MME.50**	Ind.suff.	Venezuela	Austria	MME	3	1	2	0,5113	6,6976	1	0,00509	13,16	1,22	0,16	dark blue	18,08	-2,02	-10,18
**AsB.38**	**MME.50**	Ind.suff.	Venezuela	Austria	MME	3	2	1	0,6105	7,5104	1	0,00503	14,93	1,22	0,18	dark blue	18,08	-2,02	-10,18
**AsB.38**	**MME.50**	Ind.suff.	Venezuela	Austria	MME	3	2	2	0,588	6,6629	1	0,00503	13,25	1,22	0,16	dark blue	18,08	-2,02	-10,18
**AsB.38**	**MME.50**	Ind.suff.	Venezuela	Austria	MME	3	3	1	0,5254	6,8863	1	0,00530	12,99	1,22	0,16	dark blue	18,08	-2,02	-10,18
**AsB.38**	**MME.50**	Ind.suff.	Venezuela	Austria	MME	3	3	2	0,5381	7,1922	1	0,00530	13,57	1,22	0,17	dark blue	18,08	-2,02	-10,18
**AsB.45**	**MME.57**	Ind.suff.	Ecuador	Austria	MME	1	1	1	0,4991	7,0331	1	0,00512	13,74	1,52	0,21	dark blue / black	17,11	-0,13	-8,22
**AsB.45**	**MME.57**	Ind.suff.	Ecuador	Austria	MME	1	1	2	0,5052	7,187	1	0,00512	14,04	1,52	0,21	dark blue / black	17,11	-0,13	-8,22
**AsB.45**	**MME.57**	Ind.suff.	Ecuador	Austria	MME	1	2	1	0,5028	6,5071	1	0,00505	12,89	1,52	0,20	dark blue / black	17,11	-0,13	-8,22
**AsB.45**	**MME.57**	Ind.suff.	Ecuador	Austria	MME	1	2	2	0,5048	6,5542	1	0,00505	12,98	1,52	0,20	dark blue / black	17,11	-0,13	-8,22
**AsB.45**	**MME.57**	Ind.suff.	Ecuador	Austria	MME	1	3	1	0,531	7,0212	1	0,00503	13,96	1,52	0,21	dark blue / black	17,11	-0,13	-8,22
**AsB.45**	**MME.57**	Ind.suff.	Ecuador	Austria	MME	1	3	2	0,5288	6,9682	1	0,00503	13,85	1,52	0,21	dark blue / black	17,11	-0,13	-8,22
**AsB.46**	**MME.58**	Ind.suff.	Ecuador	Austria	MME	2	1	1	0,4209	4,4054	1	0,00508	8,67	1,50	0,13	dark blue / black	18,56	-1,1	-7,34
**AsB.46**	**MME.58**	Ind.suff.	Ecuador	Austria	MME	2	1	2	0,4193	4,364	1	0,00508	8,59	1,50	0,13	dark blue / black	18,56	-1,1	-7,34
**AsB.46**	**MME.58**	Ind.suff.	Ecuador	Austria	MME	2	2	1	0,42	4,5542	1	0,00514	8,86	1,50	0,13	dark blue / black	18,56	-1,1	-7,34
**AsB.46**	**MME.58**	Ind.suff.	Ecuador	Austria	MME	2	2	2	0,41	4,3184	1	0,00514	8,40	1,50	0,13	dark blue / black	18,56	-1,1	-7,34
**AsB.46**	**MME.58**	Ind.suff.	Ecuador	Austria	MME	2	3	1	0,4281	4,5423	1	0,00521	8,72	1,50	0,13	dark blue / black	18,56	-1,1	-7,34
**AsB.46**	**MME.58**	Ind.suff.	Ecuador	Austria	MME	2	3	2	0,4317	4,629	1	0,00521	8,88	1,50	0,13	dark blue / black	18,56	-1,1	-7,34
**AsB.47**	**MME.59**	Ind.suff.	Ecuador	Austria	MME	3	1	1	0,4179	4,3277	1	0,00536	8,07	1,44	0,12	dark blue / black	18,11	-0,45	-8,4
**AsB.47**	**MME.59**	Ind.suff.	Ecuador	Austria	MME	3	1	2	0,4148	4,2474	1	0,00536	7,92	1,44	0,11	dark blue / black	18,11	-0,45	-8,4
**AsB.47**	**MME.59**	Ind.suff.	Ecuador	Austria	MME	3	2	1	0,4033	4,1604	1	0,00514	8,09	1,44	0,12	dark blue / black	18,11	-0,45	-8,4
**AsB.47**	**MME.59**	Ind.suff.	Ecuador	Austria	MME	3	2	2	0,4045	4,1887	1	0,00514	8,15	1,44	0,12	dark blue / black	18,11	-0,45	-8,4
**AsB.47**	**MME.59**	Ind.suff.	Ecuador	Austria	MME	3	3	1	0,4046	4,0702	1	0,00529	7,69	1,44	0,11	dark blue / black	18,11	-0,45	-8,4
**AsB.47**	**MME.59**	Ind.suff.	Ecuador	Austria	MME	3	3	2	0,4074	4,1381	1	0,00529	7,82	1,44	0,11	dark blue / black	18,11	-0,45	-8,4
**AsB.48**	**MME.60**	Ind.suff.	Brazil	Austria	MME	1	1	1	0,4099	4,1205	1	0,00516	7,99	1,00	0,08	dark blue	19,37	-1,14	-6,25
**AsB.48**	**MME.60**	Ind.suff.	Brazil	Austria	MME	1	1	2	0,4253	4,5194	1	0,00516	8,76	1,00	0,09	dark blue	19,37	-1,14	-6,25
**AsB.48**	**MME.60**	Ind.suff.	Brazil	Austria	MME	1	2	1	0,4137	4,4057	1	0,00506	8,71	1,00	0,09	dark blue	19,37	-1,14	-6,25
**AsB.48**	**MME.60**	Ind.suff.	Brazil	Austria	MME	1	2	2	0,4189	4,5283	1	0,00506	8,95	1,00	0,09	dark blue	19,37	-1,14	-6,25
**AsB.48**	**MME.60**	Ind.suff.	Brazil	Austria	MME	1	3	1	0,4153	4,3299	1	0,00518	8,36	1,00	0,08	dark blue	19,37	-1,14	-6,25
**AsB.48**	**MME.60**	Ind.suff.	Brazil	Austria	MME	1	3	2	0,4135	4,2862	1	0,00518	8,27	1,00	0,08	dark blue	19,37	-1,14	-6,25
**AsB.49**	**MME.61**	Ind.suff.	Brazil	Austria	MME	2	1	1	0,3833	3,4313	1	0,00523	6,56	0,66	0,04	dark blue	20,63	-1,06	-7,97
**AsB.49**	**MME.61**	Ind.suff.	Brazil	Austria	MME	2	1	2	0,3863	3,5091	1	0,00523	6,71	0,66	0,04	dark blue	20,63	-1,06	-7,97
**AsB.49**	**MME.61**	Ind.suff.	Brazil	Austria	MME	2	2	1	0,3746	3,4835	1	0,00518	6,72	0,66	0,04	dark blue	20,63	-1,06	-7,97
**AsB.49**	**MME.61**	Ind.suff.	Brazil	Austria	MME	2	2	2	0,3748	3,4882	1	0,00518	6,73	0,66	0,04	dark blue	20,63	-1,06	-7,97
**AsB.49**	**MME.61**	Ind.suff.	Brazil	Austria	MME	2	3	1	0,3812	3,5021	1	0,00520	6,73	0,66	0,04	dark blue	20,63	-1,06	-7,97
**AsB.49**	**MME.61**	Ind.suff.	Brazil	Austria	MME	2	3	2	0,381	3,4972	1	0,00520	6,73	0,66	0,04	dark blue	20,63	-1,06	-7,97
**AsB.50**	**MME.62**	Ind.suff.	Brazil	Austria	MME	3	1	1	0,2979	1,2189	1	0,00526	2,32	0,74	0,02	grey blue	24,16	-1,45	-0,45
**AsB.50**	**MME.62**	Ind.suff.	Brazil	Austria	MME	3	1	2	0,3089	1,5039	1	0,00526	2,86	0,74	0,02	grey blue	24,16	-1,45	-0,45
**AsB.50**	**MME.62**	Ind.suff.	Brazil	Austria	MME	3	2	1	0,2997	1,717	1	0,00523	3,28	0,74	0,02	grey blue	24,16	-1,45	-0,45
**AsB.50**	**MME.62**	Ind.suff.	Brazil	Austria	MME	3	2	2	0,3035	1,8066	1	0,00523	3,45	0,74	0,03	grey blue	24,16	-1,45	-0,45
**AsB.50**	**MME.62**	Ind.suff.	Brazil	Austria	MME	3	3	1	0,2976	1,4724	1	0,00516	2,85	0,74	0,02	grey blue	24,16	-1,45	-0,45
**AsB.50**	**MME.62**	Ind.suff.	Brazil	Austria	MME	3	3	2	0,3012	1,5598	1	0,00516	3,02	0,74	0,02	grey blue	24,16	-1,45	-0,45
**AsB.09**	**MME.21**	Ind.suff.	Yu Dao	China	MME	1	1	1	0,6245	10,636	1	0,00536	19,84	3,36	0,67	dark blue / black	12,62	1,05	-7,84
**AsB.09**	**MME.21**	Ind.suff.	Yu Dao	China	MME	1	1	2	0,6351	10,891	1	0,00536	20,32	3,36	0,68	dark blue / black	12,62	1,05	-7,84
**AsB.09**	**MME.21**	Ind.suff.	Yu Dao	China	MME	1	2	1	0,6206	9,7279	1	0,00506	19,23	3,36	0,65	dark blue / black	12,62	1,05	-7,84

**AsB.09**	**MME.21**	Ind.suff.	Yu Dao	China	MME	1	2	2	0,6224	9,778	1	0,00506	19,32	3,36	0,65	dark blue / black	12,62	1,05	-7,84
**AsB.09**	**MME.21**	Ind.suff.	Yu Dao	China	MME	1	3	1	0,6353	11,081	1	0,00517	21,43	3,36	0,72	dark blue / black	12,62	1,05	-7,84
**AsB.09**	**MME.21**	Ind.suff.	Yu Dao	China	MME	1	3	2	0,6129	10,496	1	0,00517	20,30	3,36	0,68	dark blue / black	12,62	1,05	-7,84
**AsB.10**	**MME.22**	Ind.suff.	Yu Dao	China	MME	2	1	1	0,5173	7,6935	1	0,00506	15,20	4,70	0,71	dark blue / black	16,27	0,13	-3,38
**AsB.10**	**MME.22**	Ind.suff.	Yu Dao	China	MME	2	1	2	0,5042	7,3193	1	0,00506	14,47	4,70	0,68	dark blue / black	16,27	0,13	-3,38
**AsB.10**	**MME.22**	Ind.suff.	Yu Dao	China	MME	2	2	1	0,5738	8,4249	1	0,00517	16,30	4,70	0,77	dark blue / black	16,27	0,13	-3,38
**AsB.10**	**MME.22**	Ind.suff.	Yu Dao	China	MME	2	2	2	0,5538	7,8681	1	0,00517	15,22	4,70	0,72	dark blue / black	16,27	0,13	-3,38
**AsB.10**	**MME.22**	Ind.suff.	Yu Dao	China	MME	2	3	1	0,5143	7,9208	1	0,00514	15,41	4,70	0,72	dark blue / black	16,27	0,13	-3,38
**AsB.10**	**MME.22**	Ind.suff.	Yu Dao	China	MME	2	3	2	0,507	7,7302	1	0,00514	15,04	4,70	0,71	dark blue / black	16,27	0,13	-3,38
**AsB.11**	**MME.23**	Ind.suff.	Yu Dao	China	MME	3	1	1	0,6459	11,151	1	0,00523	21,32	3,62	0,77	dark blue / black	13,39	0,94	-6,44
**AsB.11**	**MME.23**	Ind.suff.	Yu Dao	China	MME	3	1	2	0,6291	10,747	1	0,00523	20,55	3,62	0,74	dark blue / black	13,39	0,94	-6,44
**AsB.11**	**MME.23**	Ind.suff.	Yu Dao	China	MME	3	2	1	0,6221	9,7696	1	0,00501	19,50	3,62	0,71	dark blue / black	13,39	0,94	-6,44
**AsB.11**	**MME.23**	Ind.suff.	Yu Dao	China	MME	3	2	2	0,6278	9,9283	1	0,00501	19,82	3,62	0,72	dark blue / black	13,39	0,94	-6,44
**AsB.11**	**MME.23**	Ind.suff.	Yu Dao	China	MME	3	3	1	0,6397	11,196	1	0,00515	21,74	3,62	0,79	dark blue / black	13,39	0,94	-6,44
**AsB.11**	**MME.23**	Ind.suff.	Yu Dao	China	MME	3	3	2	0,6318	10,99	1	0,00515	21,34	3,62	0,77	dark blue / black	13,39	0,94	-6,44
**AsB.51**	**MME.63**	Ind.arr.	Mali	Austria	MME	1	1	1	1,3282	28,627	1	0,00503	56,91	1,52	0,87	dark blue / violet	11	3,32	-9,92
**AsB.51**	**MME.63**	Ind.arr.	Mali	Austria	MME	1	1	2	1,334	28,761	1	0,00503	57,18	1,52	0,87	dark blue / violet	11	3,32	-9,92
**AsB.51**	**MME.63**	Ind.arr.	Mali	Austria	MME	1	2	1	1,288	28,909	1	0,00506	57,13	1,52	0,87	dark blue / violet	11	3,32	-9,92
**AsB.51**	**MME.63**	Ind.arr.	Mali	Austria	MME	1	2	2	1,2878	28,904	1	0,00506	57,12	1,52	0,87	dark blue / violet	11	3,32	-9,92
**AsB.51**	**MME.63**	Ind.arr.	Mali	Austria	MME	1	3	1	1,3776	29,288	1	0,00534	54,85	1,52	0,83	dark blue / violet	11	3,32	-9,92
**AsB.51**	**MME.63**	Ind.arr.	Mali	Austria	MME	1	3	2	1,3994	29,866	1	0,00534	55,93	1,52	0,85	dark blue / violet	11	3,32	-9,92
**AsB.52**	**MME.64**	Ind.arr.	Mali	Austria	MME	2	1	1	1,3765	29,741	1	0,00505	58,89	1,54	0,91	dark blue / violet	9,53	3,46	-9,25
**AsB.52**	**MME.64**	Ind.arr.	Mali	Austria	MME	2	1	2	1,3907	30,062	1	0,00505	59,53	1,54	0,92	dark blue / violet	9,53	3,46	-9,25
**AsB.52**	**MME.64**	Ind.arr.	Mali	Austria	MME	2	2	1	1,3308	29,927	1	0,00502	59,62	1,54	0,92	dark blue / violet	9,53	3,46	-9,25
**AsB.52**	**MME.64**	Ind.arr.	Mali	Austria	MME	2	2	2	1,2696	28,471	1	0,00502	56,72	1,54	0,87	dark blue / violet	9,53	3,46	-9,25
**AsB.52**	**MME.64**	Ind.arr.	Mali	Austria	MME	2	3	1	1,3402	28,296	1	0,00500	56,59	1,54	0,87	dark blue / violet	9,53	3,46	-9,25
**AsB.52**	**MME.64**	Ind.arr.	Mali	Austria	MME	2	3	2	1,3578	28,763	1	0,00500	57,53	1,54	0,89	dark blue / violet	9,53	3,46	-9,25
**AsB.53**	**MME.65**	Ind.arr.	Mali	Austria	MME	3	1	1	1,3918	28,721	1	0,00509	56,43	1,88	1,06	dark blue / violet	11,63	3,3	-9,8
**AsB.53**	**MME.65**	Ind.arr.	Mali	Austria	MME	3	1	2	1,4088	29,115	1	0,00509	57,20	1,88	1,08	dark blue / violet	11,63	3,3	-9,8
**AsB.53**	**MME.65**	Ind.arr.	Mali	Austria	MME	3	2	1	1,3667	30,726	1	0,00506	60,72	1,88	1,14	dark blue / violet	11,63	3,3	-9,8
**AsB.53**	**MME.65**	Ind.arr.	Mali	Austria	MME	3	2	2	1,3425	30,191	1	0,00506	59,67	1,88	1,12	dark blue / violet	11,63	3,3	-9,8
**AsB.53**	**MME.65**	Ind.arr.	Mali	Austria	MME	3	3	1	1,4707	31,751	1	0,00528	60,13	1,88	1,13	dark blue / violet	11,63	3,3	-9,8
**AsB.53**	**MME.65**	Ind.arr.	Mali	Austria	MME	3	3	2	1,4958	32,414	1	0,00528	61,39	1,88	1,15	dark blue / violet	11,63	3,3	-9,8
**AsB.54**	**MME.66**	Ind.arr.	Burkina Faso	Austria	MME	1	1	1	1,1898	24,047	1	0,00504	47,71	1,34	0,64	dark blue / violet	10,34	3,34	-10,4
**AsB.54**	**MME.66**	Ind.arr.	Burkina Faso	Austria	MME	1	1	2	1,1586	23,324	1	0,00504	46,28	1,34	0,62	dark blue / violet	10,34	3,34	-10,4
**AsB.54**	**MME.66**	Ind.arr.	Burkina Faso	Austria	MME	1	2	1	1,1859	26,48	1	0,00508	52,13	1,34	0,70	dark blue / violet	10,34	3,34	-10,4
**AsB.54**	**MME.66**	Ind.arr.	Burkina Faso	Austria	MME	1	2	2	1,2035	26,899	1	0,00508	52,95	1,34	0,71	dark blue / violet	10,34	3,34	-10,4
**AsB.54**	**MME.66**	Ind.arr.	Burkina Faso	Austria	MME	1	3	1	1,1905	24,325	1	0,00506	48,07	1,34	0,64	dark blue / violet	10,34	3,34	-10,4
**AsB.54**	**MME.66**	Ind.arr.	Burkina Faso	Austria	MME	1	3	2	1,2133	24,93	1	0,00506	49,27	1,34	0,66	dark blue / violet	10,34	3,34	-10,4
**AsB.55**	**MME.67**	Ind.arr.	Burkina Faso	Austria	MME	2	1	1	1,0899	21,735	1	0,00526	41,32	1,14	0,47	dark blue / violet	10,33	2,74	-10,65
**AsB.55**	**MME.67**	Ind.arr.	Burkina Faso	Austria	MME	2	1	2	1,1127	22,262	1	0,00526	42,32	1,14	0,48	dark blue / violet	10,33	2,74	-10,65
**AsB.55**	**MME.67**	Ind.arr.	Burkina Faso	Austria	MME	2	2	1	1,039	22,985	1	0,00519	44,29	1,14	0,50	dark blue / violet	10,33	2,74	-10,65
**AsB.55**	**MME.67**	Ind.arr.	Burkina Faso	Austria	MME	2	2	2	1,0505	23,258	1	0,00519	44,81	1,14	0,51	dark blue / violet	10,33	2,74	-10,65
**AsB.55**	**MME.67**	Ind.arr.	Burkina Faso	Austria	MME	2	3	1	1,0994	21,908	1	0,00537	40,80	1,14	0,47	dark blue / violet	10,33	2,74	-10,65
**AsB.55**	**MME.67**	Ind.arr.	Burkina Faso	Austria	MME	2	3	2	1,0967	21,837	1	0,00537	40,66	1,14	0,46	dark blue / violet	10,33	2,74	-10,65
**AsB.56**	**MME.68**	Ind.arr.	Burkina Faso	Austria	MME	3	1	1	1,2526	25,5	1	0,00511	49,90	1,50	0,75	dark blue / violet	13,13	4,64	-11,63
**AsB.56**	**MME.68**	Ind.arr.	Burkina Faso	Austria	MME	3	1	2	1,3136	26,912	1	0,00511	52,67	1,50	0,79	dark blue / violet	13,13	4,64	-11,63
**AsB.56**	**MME.68**	Ind.arr.	Burkina Faso	Austria	MME	3	2	1	1,3459	30,266	1	0,00529	57,21	1,50	0,86	dark blue / violet	13,13	4,64	-11,63
**AsB.56**	**MME.68**	Ind.arr.	Burkina Faso	Austria	MME	3	2	2	1,3364	30,056	1	0,00529	56,82	1,50	0,85	dark blue / violet	13,13	4,64	-11,63
**AsB.56**	**MME.68**	Ind.arr.	Burkina Faso	Austria	MME	3	3	1	1,3228	27,834	1	0,00502	55,45	1,50	0,83	dark blue / violet	13,13	4,64	-11,63
**AsB.56**	**MME.68**	Ind.arr.	Burkina Faso	Austria	MME	3	3	2	1,3429	28,367	1	0,00502	56,51	1,50	0,85	dark blue / violet	13,13	4,64	-11,63
**AsB.42**	**MME.54**	Ind.arr.	unknown	Austria	MME	1	1	1	1,4619	30,639	1	0,00530	57,81	1,18	0,68	dark blue / violet	12,97	2,58	-8,16
**AsB.42**	**MME.54**	Ind.arr.	unknown	Austria	MME	1	1	2	1,4635	30,676	1	0,00530	57,88	1,18	0,68	dark blue / violet	12,97	2,58	-8,16
**AsB.42**	**MME.54**	Ind.arr.	unknown	Austria	MME	1	2	1	1,3136	27,427	1	0,00508	53,99	1,18	0,64	dark blue / violet	12,97	2,58	-8,16
**AsB.42**	**MME.54**	Ind.arr.	unknown	Austria	MME	1	2	2	1,3283	27,814	1	0,00508	54,75	1,18	0,65	dark blue / violet	12,97	2,58	-8,16
**AsB.42**	**MME.54**	Ind.arr.	unknown	Austria	MME	1	3	1	1,4612	29,552	1	0,00532	55,55	1,18	0,66	dark blue / violet	12,97	2,58	-8,16
**AsB.42**	**MME.54**	Ind.arr.	unknown	Austria	MME	1	3	2	1,4398	29,038	1	0,00532	54,58	1,18	0,64	dark blue / violet	12,97	2,58	-8,16
**AsB.43**	**MME.55**	Ind.arr.	unknown	Austria	MME	2	1	1	1,5236	32,073	1	0,00537	59,73	1,42	0,85	dark blue / violet	13,21	3,96	-9,96
**AsB.43**	**MME.55**	Ind.arr.	unknown	Austria	MME	2	1	2	1,4228	29,704	1	0,00537	55,31	1,42	0,79	dark blue / violet	13,21	3,96	-9,96
**AsB.43**	**MME.55**	Ind.arr.	unknown	Austria	MME	2	2	1	1,4262	30,428	1	0,00519	58,63	1,42	0,83	dark blue / violet	13,21	3,96	-9,96
**AsB.43**	**MME.55**	Ind.arr.	unknown	Austria	MME	2	2	2	1,4318	30,589	1	0,00519	58,94	1,42	0,84	dark blue / violet	13,21	3,96	-9,96

**AsB.43**	**MME.55**	Ind.arr.	unknown	Austria	MME	2	3	1	1,4875	30,197	1	0,00521	57,96	1,42	0,82	dark blue / violet	13,21	3,96	-9,96
**AsB.43**	**MME.55**	Ind.arr.	unknown	Austria	MME	2	3	2	1,4885	30,223	1	0,00521	58,01	1,42	0,82	dark blue / violet	13,21	3,96	-9,96
**AsB.44**	**MME.56**	Ind.arr.	unknown	Austria	MME	3	1	1	1,477	31,885	1	0,00531	60,05	1,72	1,03	dark blue / violet	10,17	3,16	-8,03
**AsB.44**	**MME.56**	Ind.arr.	unknown	Austria	MME	3	1	2	1,4506	31,328	1	0,00531	59,00	1,72	1,01	dark blue / violet	10,17	3,16	-8,03
**AsB.44**	**MME.56**	Ind.arr.	unknown	Austria	MME	3	2	1	1,484	32,089	1	0,00520	61,71	1,72	1,06	dark blue / violet	10,17	3,16	-8,03
**AsB.44**	**MME.56**	Ind.arr.	unknown	Austria	MME	3	2	2	1,5324	33,48	1	0,00520	64,38	1,72	1,11	dark blue / violet	10,17	3,16	-8,03
**AsB.44**	**MME.56**	Ind.arr.	unknown	Austria	MME	3	3	1	1,489	30,236	1	0,00526	57,48	1,72	0,99	dark blue / violet	10,17	3,16	-8,03
**AsB.44**	**MME.56**	Ind.arr.	unknown	Austria	MME	3	3	2	1,5515	31,836	1	0,00526	60,52	1,72	1,04	dark blue / violet	10,17	3,16	-8,03
**AsB.57**	**MME.69**	Ind.arr.	unknown	Austria	mod.MME	1	1	1	1,4635	30,394	1	0,00500	60,79	1,90	1,15	dark blue / violet	9,89	2,24	-8,02
**AsB.57**	**MME.69**	Ind.arr.	unknown	Austria	mod.MME	1	1	2	1,4311	29,631	1	0,00500	59,26	1,90	1,13	dark blue / violet	9,89	2,24	-8,02
**AsB.57**	**MME.69**	Ind.arr.	unknown	Austria	mod.MME	1	2	1	1,4685	32,975	1	0,00527	62,57	1,90	1,19	dark blue / violet	9,89	2,24	-8,02
**AsB.57**	**MME.69**	Ind.arr.	unknown	Austria	mod.MME	1	2	2	1,434	32,213	1	0,00527	61,13	1,90	1,16	dark blue / violet	9,89	2,24	-8,02
**AsB.57**	**MME.69**	Ind.arr.	unknown	Austria	mod.MME	1	3	1	1,4789	31,328	1	0,00529	59,22	1,90	1,13	dark blue / violet	9,89	2,24	-8,02
**AsB.57**	**MME.69**	Ind.arr.	unknown	Austria	mod.MME	1	3	2	1,4743	31,224	1	0,00529	59,02	1,90	1,12	dark blue / violet	9,89	2,24	-8,02
**AsB.58**	**MME.70**	Ind.arr.	unknown	Austria	mod.MME	2	1	1	1,3304	27,3	1	0,00515	53,01	2,26	1,20	dark blue / violet	10,18	2,71	-8,88
**AsB.58**	**MME.70**	Ind.arr.	unknown	Austria	mod.MME	2	1	2	1,3488	27,726	1	0,00515	53,84	2,26	1,22	dark blue / violet	10,18	2,71	-8,88
**AsB.58**	**MME.70**	Ind.arr.	unknown	Austria	mod.MME	2	2	1	1,2933	29,035	1	0,00502	57,84	2,26	1,31	dark blue / violet	10,18	2,71	-8,88
**AsB.58**	**MME.70**	Ind.arr.	unknown	Austria	mod.MME	2	2	2	1,2887	28,926	1	0,00502	57,62	2,26	1,30	dark blue / violet	10,18	2,71	-8,88
**AsB.58**	**MME.70**	Ind.arr.	unknown	Austria	mod.MME	2	3	1	1,3418	27,989	1	0,00523	53,52	2,26	1,21	dark blue / violet	10,18	2,71	-8,88
**AsB.58**	**MME.70**	Ind.arr.	unknown	Austria	mod.MME	2	3	2	1,3452	28,076	1	0,00523	53,68	2,26	1,21	dark blue / violet	10,18	2,71	-8,88
**AsB.59**	**MME.71**	Ind.arr.	unknown	Austria	mod.MME	3	1	1	1,4372	29,772	1	0,00538	55,34	2,34	1,29	dark blue / violet	10,38	2,38	-8,67
**AsB.59**	**MME.71**	Ind.arr.	unknown	Austria	mod.MME	3	1	2	1,4303	29,612	1	0,00538	55,04	2,34	1,29	dark blue / violet	10,38	2,38	-8,67
**AsB.59**	**MME.71**	Ind.arr.	unknown	Austria	mod.MME	3	2	1	1,444	32,433	1	0,00520	62,37	2,34	1,46	dark blue / violet	10,38	2,38	-8,67
**AsB.59**	**MME.71**	Ind.arr.	unknown	Austria	mod.MME	3	2	2	1,4229	31,967	1	0,00520	61,48	2,34	1,44	dark blue / violet	10,38	2,38	-8,67
**AsB.59**	**MME.71**	Ind.arr.	unknown	Austria	mod.MME	3	3	1	1,444	30,538	1	0,00516	59,18	2,34	1,38	dark blue / violet	10,38	2,38	-8,67
**AsB.59**	**MME.71**	Ind.arr.	unknown	Austria	mod.MME	3	3	2	1,4538	30,76	1	0,00516	59,61	2,34	1,39	dark blue / violet	10,38	2,38	-8,67
**AsB.24**	**MME.30**	Pers.tinc.	"Senbon"	Austria	MME	1	1	1	0,8695	17,211	1	0,00527	32,66	1,06	0,35	dark blue / violet	11,66	2,18	-10,4
**AsB.24**	**MME.30**	Pers.tinc.	"Senbon"	Austria	MME	1	1	2	0,8775	17,434	1	0,00527	33,08	1,06	0,35	dark blue / violet	11,66	2,18	-10,4
**AsB.24**	**MME.30**	Pers.tinc.	"Senbon"	Austria	MME	1	2	1	0,8704	16,179	1	0,00502	32,23	1,06	0,34	dark blue / violet	11,66	2,18	-10,4
**AsB.24**	**MME.30**	Pers.tinc.	"Senbon"	Austria	MME	1	2	2	0,8898	16,615	1	0,00502	33,10	1,06	0,35	dark blue / violet	11,66	2,18	-10,4
**AsB.24**	**MME.30**	Pers.tinc.	"Senbon"	Austria	MME	1	3	1	0,948	16,648	1	0,00525	31,71	1,06	0,34	dark blue / violet	11,66	2,18	-10,4
**AsB.24**	**MME.30**	Pers.tinc.	"Senbon"	Austria	MME	1	3	2	0,9416	16,492	1	0,00525	31,41	1,06	0,33	dark blue / violet	11,66	2,18	-10,4
**AsB.25**	**MME.31**	Pers.tinc.	"Senbon"	Austria	MME	2	1	1	0,8385	16,346	1	0,00509	32,11	1,04	0,33	dark blue / violet	10,85	1,98	-10,51
**AsB.25**	**MME.31**	Pers.tinc.	"Senbon"	Austria	MME	2	1	2	0,8293	16,089	1	0,00509	31,61	1,04	0,33	dark blue / violet	10,85	1,98	-10,51
**AsB.25**	**MME.31**	Pers.tinc.	"Senbon"	Austria	MME	2	2	1	0,8425	15,552	1	0,00510	30,49	1,04	0,32	dark blue / violet	10,85	1,98	-10,51
**AsB.25**	**MME.31**	Pers.tinc.	"Senbon"	Austria	MME	2	2	2	0,8584	15,909	1	0,00510	31,19	1,04	0,32	dark blue / violet	10,85	1,98	-10,51
**AsB.25**	**MME.31**	Pers.tinc.	"Senbon"	Austria	MME	2	3	1	0,8704	14,756	1	0,00510	28,93	1,04	0,30	dark blue / violet	10,85	1,98	-10,51
**AsB.25**	**MME.31**	Pers.tinc.	"Senbon"	Austria	MME	2	3	2	0,874	14,843	1	0,00510	29,10	1,04	0,30	dark blue / violet	10,85	1,98	-10,51
**AsB.26**	**MME.32**	Pers.tinc.	"Senbon"	Austria	MME	3	1	1	0,8698	17,219	1	0,00505	34,10	0,98	0,33	dark blue / violet	10,71	2,71	-10,66
**AsB.26**	**MME.32**	Pers.tinc.	"Senbon"	Austria	MME	3	1	2	0,8784	17,459	1	0,00505	34,57	0,98	0,34	dark blue / violet	10,71	2,71	-10,66
**AsB.26**	**MME.32**	Pers.tinc.	"Senbon"	Austria	MME	3	2	1	0,8795	16,383	1	0,00517	31,69	0,98	0,31	dark blue / violet	10,71	2,71	-10,66
**AsB.26**	**MME.32**	Pers.tinc.	"Senbon"	Austria	MME	3	2	2	0,8786	16,363	1	0,00517	31,65	0,98	0,31	dark blue / violet	10,71	2,71	-10,66
**AsB.26**	**MME.32**	Pers.tinc.	"Senbon"	Austria	MME	3	3	1	0,9289	16,182	1	0,00515	31,42	0,98	0,31	dark blue / violet	10,71	2,71	-10,66
**AsB.26**	**MME.32**	Pers.tinc.	"Senbon"	Austria	MME	3	3	2	0,9455	16,587	1	0,00515	32,21	0,98	0,32	dark blue / violet	10,71	2,71	-10,66
**AsB.27**	**MME.33**	Pers.tinc.	"Kojyoko"	Austria	MME	1	1	1	1,2226	26,357	1	0,00513	51,38	1,54	0,79	dark blue / violet	9,89	2,52	-10,62
**AsB.27**	**MME.33**	Pers.tinc.	"Kojyoko"	Austria	MME	1	1	2	1,2467	26,962	1	0,00513	52,56	1,54	0,81	dark blue / violet	9,89	2,52	-10,62
**AsB.27**	**MME.33**	Pers.tinc.	"Kojyoko"	Austria	MME	1	2	1	1,1771	24,499	1	0,00506	48,42	1,54	0,75	dark blue / violet	9,89	2,52	-10,62
**AsB.27**	**MME.33**	Pers.tinc.	"Kojyoko"	Austria	MME	1	2	2	1,1456	23,462	1	0,00506	46,37	1,54	0,71	dark blue / violet	9,89	2,52	-10,62
**AsB.27**	**MME.33**	Pers.tinc.	"Kojyoko"	Austria	MME	1	3	1	1,2465	24,299	1	0,00503	48,31	1,54	0,74	dark blue / violet	9,89	2,52	-10,62
**AsB.27**	**MME.33**	Pers.tinc.	"Kojyoko"	Austria	MME	1	3	2	1,2326	23,928	1	0,00503	47,57	1,54	0,73	dark blue / violet	9,89	2,52	-10,62
**AsB.28**	**MME.34**	Pers.tinc.	"Kojyoko"	Austria	MME	2	1	1	1,2693	27,529	1	0,00514	53,56	1,80	0,96	dark blue / violet	8,47	3,07	-10,02
**AsB.28**	**MME.34**	Pers.tinc.	"Kojyoko"	Austria	MME	2	1	2	1,3134	28,637	1	0,00514	55,71	1,80	1,00	dark blue / violet	8,47	3,07	-10,02
**AsB.28**	**MME.34**	Pers.tinc.	"Kojyoko"	Austria	MME	2	2	1	1,2592	27,204	1	0,00518	52,52	1,80	0,95	dark blue / violet	8,47	3,07	-10,02
**AsB.28**	**MME.34**	Pers.tinc.	"Kojyoko"	Austria	MME	2	2	2	1,2542	27,039	1	0,00518	52,20	1,80	0,94	dark blue / violet	8,47	3,07	-10,02
**AsB.28**	**MME.34**	Pers.tinc.	"Kojyoko"	Austria	MME	2	3	1	1,2309	24,905	1	0,00516	48,27	1,80	0,87	dark blue / violet	8,47	3,07	-10,02
**AsB.28**	**MME.34**	Pers.tinc.	"Kojyoko"	Austria	MME	2	3	2	1,2291	24,857	1	0,00516	48,17	1,80	0,87	dark blue / violet	8,47	3,07	-10,02
**AsB.29**	**MME.35**	Pers.tinc.	"Kojyoko"	Austria	MME	3	1	1	1,2428	26,864	1	0,00512	52,47	1,68	0,88	dark blue / violet	9,23	2,81	-10,13
**AsB.29**	**MME.35**	Pers.tinc.	"Kojyoko"	Austria	MME	3	1	2	1,255	27,17	1	0,00512	53,07	1,68	0,89	dark blue / violet	9,23	2,81	-10,13
**AsB.29**	**MME.35**	Pers.tinc.	"Kojyoko"	Austria	MME	3	2	1	1,2326	26,327	1	0,00517	50,92	1,68	0,86	dark blue / violet	9,23	2,81	-10,13
**AsB.29**	**MME.35**	Pers.tinc.	"Kojyoko"	Austria	MME	3	2	2	1,2188	25,873	1	0,00517	50,04	1,68	0,84	dark blue / violet	9,23	2,81	-10,13
**AsB.29**	**MME.35**	Pers.tinc.	"Kojyoko"	Austria	MME	3	3	1	1,1625	23,073	1	0,00518	44,54	1,68	0,75	dark blue / violet	9,23	2,81	-10,13

**AsB.29**	**MME.35**	Pers.tinc.	"Kojyoko"	Austria	MME	3	3	2	1,2614	25,722	1	0,00518	49,66	1,68	0,83	dark blue / violet	9,23	2,81	-10,13
**AsB.30**	**MME.36**	Pers.tinc.	"Maruba"	Austria	MME	1	1	1	1,1182	24,386	1	0,00506	48,19	1,40	0,67	dark blue / violet	9,1	2,78	-10,25
**AsB.30**	**MME.36**	Pers.tinc.	"Maruba"	Austria	MME	1	1	2	1,0838	23,702	1	0,00506	46,84	1,40	0,66	dark blue / violet	9,1	2,78	-10,25
**AsB.30**	**MME.36**	Pers.tinc.	"Maruba"	Austria	MME	1	2	1	1,2508	26,927	1	0,00512	52,59	1,40	0,74	dark blue / violet	9,1	2,78	-10,25
**AsB.30**	**MME.36**	Pers.tinc.	"Maruba"	Austria	MME	1	2	2	1,2088	25,543	1	0,00512	49,89	1,40	0,70	dark blue / violet	9,1	2,78	-10,25
**AsB.30**	**MME.36**	Pers.tinc.	"Maruba"	Austria	MME	1	3	1	1,2013	24,112	1	0,00535	45,07	1,40	0,63	dark blue / violet	9,1	2,78	-10,25
**AsB.30**	**MME.36**	Pers.tinc.	"Maruba"	Austria	MME	1	3	2	1,2181	24,562	1	0,00535	45,91	1,40	0,64	dark blue / violet	9,1	2,78	-10,25
**AsB.31**	**MME.37**	Pers.tinc.	"Maruba"	Austria	MME	2	1	1	1,2433	26,874	1	0,00529	50,80	1,22	0,62	dark blue / violet	9,6	3,2	-9,53
**AsB.31**	**MME.37**	Pers.tinc.	"Maruba"	Austria	MME	2	1	2	1,2734	27,472	1	0,00529	51,93	1,22	0,63	dark blue / violet	9,6	3,2	-9,53
**AsB.31**	**MME.37**	Pers.tinc.	"Maruba"	Austria	MME	2	2	1	1,0332	21,677	1	0,00517	41,93	1,22	0,51	dark blue / violet	9,6	3,2	-9,53
**AsB.31**	**MME.37**	Pers.tinc.	"Maruba"	Austria	MME	2	2	2	1,1129	23,737	1	0,00517	45,91	1,22	0,56	dark blue / violet	9,6	3,2	-9,53
**AsB.31**	**MME.37**	Pers.tinc.	"Maruba"	Austria	MME	2	3	1	1,2258	24,768	1	0,00507	48,85	1,22	0,60	dark blue / violet	9,6	3,2	-9,53
**AsB.31**	**MME.37**	Pers.tinc.	"Maruba"	Austria	MME	2	3	2	1,2426	25,218	1	0,00507	49,74	1,22	0,61	dark blue / violet	9,6	3,2	-9,53
**AsB.32**	**MME.38**	Pers.tinc.	"Maruba"	Austria	MME	3	1	1	1,3382	28,761	1	0,00527	54,57	1,32	0,72	dark blue / violet	8,31	3,54	-10,12
**AsB.32**	**MME.38**	Pers.tinc.	"Maruba"	Austria	MME	3	1	2	1,2833	27,669	1	0,00527	52,50	1,32	0,69	dark blue / violet	8,31	3,54	-10,12
**AsB.32**	**MME.38**	Pers.tinc.	"Maruba"	Austria	MME	3	2	1	1,2528	27,354	1	0,00525	52,10	1,32	0,69	dark blue / violet	8,31	3,54	-10,12
**AsB.32**	**MME.38**	Pers.tinc.	"Maruba"	Austria	MME	3	2	2	1,1632	25,037	1	0,00525	47,69	1,32	0,63	dark blue / violet	8,31	3,54	-10,12
**AsB.32**	**MME.38**	Pers.tinc.	"Maruba"	Austria	MME	3	3	1	1,3062	26,922	1	0,00523	51,48	1,32	0,68	dark blue / violet	8,31	3,54	-10,12
**AsB.32**	**MME.38**	Pers.tinc.	"Maruba"	Austria	MME	3	3	2	1,3203	27,299	1	0,00523	52,20	1,32	0,69	dark blue / violet	8,31	3,54	-10,12
**AsB.01**	**MME.10**	Stro.cus.	Xin Cun	China	MME	1	1	1	1,1959	24,363	1	0,00504	48,34	4,48	2,17	dark blue / violet	8,85	3,02	-11,91
**AsB.01**	**MME.10**	Stro.cus.	Xin Cun	China	MME	1	1	2	1,2207	24,98	1	0,00504	49,56	4,48	2,22	dark blue / violet	8,85	3,02	-11,91
**AsB.01**	**MME.10**	Stro.cus.	Xin Cun	China	MME	1	2	1	1,2747	28,453	1	0,00516	55,14	4,48	2,47	dark blue / violet	8,85	3,02	-11,91
**AsB.01**	**MME.10**	Stro.cus.	Xin Cun	China	MME	1	2	2	1,2646	28,189	1	0,00516	54,63	4,48	2,45	dark blue / violet	8,85	3,02	-11,91
**AsB.01**	**MME.10**	Stro.cus.	Xin Cun	China	MME	1	3	1	1,2648	26,978	1	0,00506	53,32	4,48	2,39	dark blue / violet	8,85	3,02	-11,91
**AsB.01**	**MME.10**	Stro.cus.	Xin Cun	China	MME	1	3	2	1,3026	27,953	1	0,00506	55,24	4,48	2,47	dark blue / violet	8,85	3,02	-11,91
**AsB.02**	**MME.11**	Stro.cus.	Xin Cun	China	MME	2	1	1	1,3496	28,189	1	0,00501	56,27	4,80	2,70	dark blue / violet	8,87	2,46	-12,21
**AsB.02**	**MME.11**	Stro.cus.	Xin Cun	China	MME	2	1	2	1,2909	26,728	1	0,00501	53,35	4,80	2,56	dark blue / violet	8,87	2,46	-12,21
**AsB.02**	**MME.11**	Stro.cus.	Xin Cun	China	MME	2	2	1	1,3089	29,344	1	0,00507	57,88	4,80	2,78	dark blue / violet	8,87	2,46	-12,21
**AsB.02**	**MME.11**	Stro.cus.	Xin Cun	China	MME	2	2	2	1,3159	29,527	1	0,00507	58,24	4,80	2,80	dark blue / violet	8,87	2,46	-12,21
**AsB.02**	**MME.11**	Stro.cus.	Xin Cun	China	MME	2	3	1	1,3732	29,772	1	0,00506	58,84	4,80	2,82	dark blue / violet	8,87	2,46	-12,21
**AsB.02**	**MME.11**	Stro.cus.	Xin Cun	China	MME	2	3	2	1,3473	29,104	1	0,00506	57,52	4,80	2,76	dark blue / violet	8,87	2,46	-12,21
**AsB.03**	**MME.12**	Stro.cus.	Xin Cun	China	MME	3	1	1	1,3572	28,378	1	0,00501	56,64	4,76	2,70	dark blue / violet	10,12	2,84	-12,05
**AsB.03**	**MME.12**	Stro.cus.	Xin Cun	China	MME	3	1	2	1,3572	28,378	1	0,00501	56,64	4,76	2,70	dark blue / violet	10,12	2,84	-12,05
**AsB.03**	**MME.12**	Stro.cus.	Xin Cun	China	MME	3	2	1	1,3331	29,975	1	0,00511	58,66	4,76	2,79	dark blue / violet	10,12	2,84	-12,05
**AsB.03**	**MME.12**	Stro.cus.	Xin Cun	China	MME	3	2	2	1,3221	29,688	1	0,00511	58,10	4,76	2,77	dark blue / violet	10,12	2,84	-12,05
**AsB.03**	**MME.12**	Stro.cus.	Xin Cun	China	MME	3	3	1	1,3012	27,917	1	0,00502	55,61	4,76	2,65	dark blue / violet	10,12	2,84	-12,05
**AsB.03**	**MME.12**	Stro.cus.	Xin Cun	China	MME	3	3	2	1,3272	28,587	1	0,00502	56,95	4,76	2,71	dark blue / violet	10,12	2,84	-12,05
**AsB.04**	**MME.13**	Wri.laev.	Xin Cun	China	mod.MME	1	1	1	1,1784	23,927	1	0,00520	46,01	1,36	0,63	dark blue / violet	8,63	2,16	-12,68
**AsB.04**	**MME.13**	Wri.laev.	Xin Cun	China	mod.MME	1	1	2	1,2117	24,756	1	0,00520	47,61	1,36	0,65	dark blue / violet	8,63	2,16	-12,68
**AsB.04**	**MME.13**	Wri.laev.	Xin Cun	China	mod.MME	1	2	1	1,1527	25,272	1	0,00506	49,94	1,36	0,68	dark blue / violet	8,63	2,16	-12,68
**AsB.04**	**MME.13**	Wri.laev.	Xin Cun	China	mod.MME	1	2	2	1,1245	24,537	1	0,00506	48,49	1,36	0,66	dark blue / violet	8,63	2,16	-12,68
**AsB.04**	**MME.13**	Wri.laev.	Xin Cun	China	mod.MME	1	3	1	1,1609	24,301	1	0,00525	46,29	1,36	0,63	dark blue / violet	8,63	2,16	-12,68
**AsB.04**	**MME.13**	Wri.laev.	Xin Cun	China	mod.MME	1	3	2	1,146	23,917	1	0,00525	45,56	1,36	0,62	dark blue / violet	8,63	2,16	-12,68
**AsB.97**	**MME.14**	Wri.laev.	Xin Cun	China	mod.MME	2	1	1	0,5939	9,5466	1	0,00508	18,79	0,90	0,17	dark blue / violet	measuring not possible (too little sample material)		
**AsB.97**	**MME.14**	Wri.laev.	Xin Cun	China	mod.MME	2	1	2	0,5956	9,5889	1	0,00508	18,88	0,90	0,17	dark blue / violet			
**AsB.97**	**MME.14**	Wri.laev.	Xin Cun	China	mod.MME	2	2	1	0,6433	9,2764	1	0,00508	18,26	0,90	0,16	dark blue / violet			
**AsB.97**	**MME.14**	Wri.laev.	Xin Cun	China	mod.MME	2	2	2	0,6176	8,6961	1	0,00508	17,12	0,90	0,15	dark blue / violet			
**AsB.97**	**MME.14**	Wri.laev.	Xin Cun	China	mod.MME	2	3	1	0,6245	9,8198	1	0,00511	19,22	0,90	0,17	dark blue / violet			
**AsB.97**	**MME.14**	Wri.laev.	Xin Cun	China	mod.MME	2	3	2	0,6549	10,567	1	0,00511	20,68	0,90	0,19	dark blue / violet			
**AsB.05**	**MME.15**	Wri.laev.	Xin Cun	China	mod.MME	3	1	1	1,1323	22,779	1	0,00510	44,66	1,84	0,82	dark blue / violet	8,34	1,97	-13,24
**AsB.05**	**MME.15**	Wri.laev.	Xin Cun	China	mod.MME	3	1	2	1,1544	23,33	1	0,00510	45,75	1,84	0,84	dark blue / violet	8,34	1,97	-13,24
**AsB.05**	**MME.15**	Wri.laev.	Xin Cun	China	mod.MME	3	2	1	1,1035	23,989	1	0,00503	47,69	1,84	0,88	dark blue / violet	8,34	1,97	-13,24
**AsB.05**	**MME.15**	Wri.laev.	Xin Cun	China	mod.MME	3	2	2	1,1549	25,329	1	0,00503	50,36	1,84	0,93	dark blue / violet	8,34	1,97	-13,24
**AsB.05**	**MME.15**	Wri.laev.	Xin Cun	China	mod.MME	3	3	1	1,1324	23,567	1	0,00503	46,85	1,84	0,86	dark blue / violet	8,34	1,97	-13,24
**AsB.05**	**MME.15**	Wri.laev.	Xin Cun	China	mod.MME	3	3	2	1,146	23,917	1	0,00503	47,55	1,84	0,87	dark blue / violet	8,34	1,97	-13,24
**AsB.06**	**MME.16**	Wri.laev.	Xin Cun	China	mod.MME	1	1	1	0,7247	12,459	1	0,00504	24,72	1,78	0,44	dark blue / violet	12,41	-0,17	-9,67
**AsB.06**	**MME.16**	Wri.laev.	Xin Cun	China	mod.MME	1	1	2	0,7209	12,362	1	0,00504	24,53	1,78	0,44	dark blue / violet	12,41	-0,17	-9,67
**AsB.06**	**MME.16**	Wri.laev.	Xin Cun	China	mod.MME	1	2	1	0,6771	12,707	1	0,00522	24,34	1,78	0,43	dark blue / violet	12,41	-0,17	-9,67
**AsB.06**	**MME.16**	Wri.laev.	Xin Cun	China	mod.MME	1	2	2	0,6751	12,654	1	0,00522	24,24	1,78	0,43	dark blue / violet	12,41	-0,17	-9,67
**AsB.06**	**MME.16**	Wri.laev.	Xin Cun	China	mod.MME	1	3	1	0,6743	12,1	1	0,00514	23,54	1,78	0,42	dark blue / violet	12,41	-0,17	-9,67
**AsB.06**	**MME.16**	Wri.laev.	Xin Cun	China	mod.MME	1	3	2	0,6527	11,536	1	0,00514	22,44	1,78	0,40	dark blue / violet	12,41	-0,17	-9,67

**AsB.07**	**MME.17**	Wri.laev.	Xin Cun	China	mod.MME	2	1	1	0,7816	13,909	1	0,00517	26,90	2,36	0,63	dark blue / violet	11,76	0,02	-11,09
**AsB.07**	**MME.17**	Wri.laev.	Xin Cun	China	mod.MME	2	1	2	0,7221	12,393	1	0,00517	23,97	2,36	0,57	dark blue / violet	11,76	0,02	-11,09
**AsB.07**	**MME.17**	Wri.laev.	Xin Cun	China	mod.MME	2	2	1	0,7527	13,406	1	0,00507	26,44	2,36	0,62	dark blue / violet	11,76	0,02	-11,09
**AsB.07**	**MME.17**	Wri.laev.	Xin Cun	China	mod.MME	2	2	2	0,7492	13,308	1	0,00507	26,25	2,36	0,62	dark blue / violet	11,76	0,02	-11,09
**AsB.07**	**MME.17**	Wri.laev.	Xin Cun	China	mod.MME	2	3	1	0,7285	13,515	1	0,00510	26,50	2,36	0,63	dark blue / violet	11,76	0,02	-11,09
**AsB.07**	**MME.17**	Wri.laev.	Xin Cun	China	mod.MME	2	3	2	0,7333	13,641	1	0,00510	26,75	2,36	0,63	dark blue / violet	11,76	0,02	-11,09
**AsB.08**	**MME.18**	Wri.laev.	Xin Cun	China	mod.MME	3	1	1	0,7867	14,539	1	0,00515	28,23	3,10	0,88	dark blue / violet	11,53	0,32	-11,72
**AsB.08**	**MME.18**	Wri.laev.	Xin Cun	China	mod.MME	3	1	2	0,7756	14,272	1	0,00515	27,71	3,10	0,86	dark blue / violet	11,53	0,32	-11,72
**AsB.08**	**MME.18**	Wri.laev.	Xin Cun	China	mod.MME	3	2	1	0,8306	15,575	1	0,00505	30,84	3,10	0,96	dark blue / violet	11,53	0,32	-11,72
**AsB.08**	**MME.18**	Wri.laev.	Xin Cun	China	mod.MME	3	2	2	0,7953	14,592	1	0,00505	28,90	3,10	0,90	dark blue / violet	11,53	0,32	-11,72
**AsB.08**	**MME.18**	Wri.laev.	Xin Cun	China	mod.MME	3	3	1	0,8205	15,918	1	0,00519	30,67	3,10	0,95	dark blue / violet	11,53	0,32	-11,72
**AsB.08**	**MME.18**	Wri.laev.	Xin Cun	China	mod.MME	3	3	2	0,8058	15,534	1	0,00519	29,93	3,10	0,93	dark blue / violet	11,53	0,32	-11,72
**AsB.60**	**sLPE.04**	Pers.tinc.	"Senbon"	Austria	sLPE	1	1	1	0,2919	1,8034	1	0,00508	3,55	14,88	0,53	light blue	36,3	-4,47	-12,32
**AsB.60**	**sLPE.04**	Pers.tinc.	"Senbon"	Austria	sLPE	1	1	2	0,2835	1,5914	1	0,00508	3,13	14,88	0,47	light blue	36,3	-4,47	-12,32
**AsB.60**	**sLPE.04**	Pers.tinc.	"Senbon"	Austria	sLPE	1	2	1	0,2908	1,4248	1	0,00538	2,65	14,88	0,39	light blue	36,3	-4,47	-12,32
**AsB.60**	**sLPE.04**	Pers.tinc.	"Senbon"	Austria	sLPE	1	2	2	0,2922	1,46	1	0,00538	2,71	14,88	0,40	light blue	36,3	-4,47	-12,32
**AsB.60**	**sLPE.04**	Pers.tinc.	"Senbon"	Austria	sLPE	1	3	1	0,2954	1,3495	1	0,00526	2,57	14,88	0,38	light blue	36,3	-4,47	-12,32
**AsB.60**	**sLPE.04**	Pers.tinc.	"Senbon"	Austria	sLPE	1	3	2	0,2936	1,3026	1	0,00526	2,48	14,88	0,37	light blue	36,3	-4,47	-12,32
**AsB.61**	**sLPE. 05**	Pers.tinc.	"Senbon"	Austria	sLPE	2	1	1	0,2944	1,8665	1	0,00530	3,52	15,24	0,54	light blue	37,63	-5,35	-9,95
**AsB.61**	**sLPE. 05**	Pers.tinc.	"Senbon"	Austria	sLPE	2	1	2	0,2952	1,8867	1	0,00530	3,56	15,24	0,54	light blue	37,63	-5,35	-9,95
**AsB.61**	**sLPE. 05**	Pers.tinc.	"Senbon"	Austria	sLPE	2	2	1	0,3019	1,7066	1	0,00516	3,31	15,24	0,50	light blue	37,63	-5,35	-9,95
**AsB.61**	**sLPE. 05**	Pers.tinc.	"Senbon"	Austria	sLPE	2	2	2	0,2984	1,6091	1	0,00516	3,12	15,24	0,48	light blue	37,63	-5,35	-9,95
**AsB.61**	**sLPE. 05**	Pers.tinc.	"Senbon"	Austria	sLPE	2	3	1	0,3066	1,6413	1	0,00515	3,19	15,24	0,49	light blue	37,63	-5,35	-9,95
**AsB.61**	**sLPE. 05**	Pers.tinc.	"Senbon"	Austria	sLPE	2	3	2	0,3075	1,6647	1	0,00515	3,23	15,24	0,49	light blue	37,63	-5,35	-9,95
**AsB.62**	**sLPE. 06**	Pers.tinc.	"Senbon"	Austria	sLPE	3	1	1	0,288	1,9476	1	0,00520	3,75	15,22	0,57	light blue	38,57	-4,48	-8,95
**AsB.62**	**sLPE. 06**	Pers.tinc.	"Senbon"	Austria	sLPE	3	1	2	0,2861	1,9004	1	0,00520	3,65	15,22	0,56	light blue	38,57	-4,48	-8,95
**AsB.62**	**sLPE. 06**	Pers.tinc.	"Senbon"	Austria	sLPE	3	2	1	0,3034	1,7483	1	0,00514	3,40	15,22	0,52	light blue	38,57	-4,48	-8,95
**AsB.62**	**sLPE. 06**	Pers.tinc.	"Senbon"	Austria	sLPE	3	2	2	0,3055	1,8068	1	0,00514	3,52	15,22	0,54	light blue	38,57	-4,48	-8,95
**AsB.62**	**sLPE. 06**	Pers.tinc.	"Senbon"	Austria	sLPE	3	3	1	0,3101	1,7324	1	0,00511	3,39	15,22	0,52	light blue	38,57	-4,48	-8,95
**AsB.62**	**sLPE. 06**	Pers.tinc.	"Senbon"	Austria	sLPE	3	3	2	0,3097	1,722	1	0,00511	3,37	15,22	0,51	light blue	38,57	-4,48	-8,95
**AsB.63**	**sLPE.07**	Pers.tinc.	"Kojyoko"	Austria	sLPE	1	1	1	0,3205	2,7549	1	0,00512	5,38	15,28	0,82	mid blue	32,23	-4,66	-10,33
**AsB.63**	**sLPE.07**	Pers.tinc.	"Kojyoko"	Austria	sLPE	1	1	2	0,3197	2,7351	1	0,00512	5,34	15,28	0,82	mid blue	32,23	-4,66	-10,33
**AsB.63**	**sLPE.07**	Pers.tinc.	"Kojyoko"	Austria	sLPE	1	2	1	0,3324	2,5557	1	0,00519	4,92	15,28	0,75	mid blue	32,23	-4,66	-10,33
**AsB.63**	**sLPE.07**	Pers.tinc.	"Kojyoko"	Austria	sLPE	1	2	2	0,3344	2,6114	1	0,00519	5,03	15,28	0,77	mid blue	32,23	-4,66	-10,33
**AsB.63**	**sLPE.07**	Pers.tinc.	"Kojyoko"	Austria	sLPE	1	3	1	0,3475	2,7068	1	0,00533	5,08	15,28	0,78	mid blue	32,23	-4,66	-10,33
**AsB.63**	**sLPE.07**	Pers.tinc.	"Kojyoko"	Austria	sLPE	1	3	2	0,3456	2,6573	1	0,00533	4,99	15,28	0,76	mid blue	32,23	-4,66	-10,33
**AsB.64**	**sLPE.08**	Pers.tinc.	"Kojyoko"	Austria	sLPE	2	1	1	0,3317	3,0332	1	0,00532	5,70	16,02	0,91	mid blue	29,93	-3,9	-11,66
**AsB.64**	**sLPE.08**	Pers.tinc.	"Kojyoko"	Austria	sLPE	2	1	2	0,3331	3,0679	1	0,00532	5,77	16,02	0,92	mid blue	29,93	-3,9	-11,66
**AsB.64**	**sLPE.08**	Pers.tinc.	"Kojyoko"	Austria	sLPE	2	2	1	0,3418	2,8174	1	0,00501	5,62	16,02	0,90	mid blue	29,93	-3,9	-11,66
**AsB.64**	**sLPE.08**	Pers.tinc.	"Kojyoko"	Austria	sLPE	2	2	2	0,36	3,3241	1	0,00501	6,63	16,02	1,06	mid blue	29,93	-3,9	-11,66
**AsB.64**	**sLPE.08**	Pers.tinc.	"Kojyoko"	Austria	sLPE	2	3	1	0,3602	3,0376	1	0,00516	5,89	16,02	0,94	mid blue	29,93	-3,9	-11,66
**AsB.64**	**sLPE.08**	Pers.tinc.	"Kojyoko"	Austria	sLPE	2	3	2	0,3637	3,1288	1	0,00516	6,06	16,02	0,97	mid blue	29,93	-3,9	-11,66
**AsB.65**	**sLPE.09**	Pers.tinc.	"Kojyoko"	Austria	sLPE	3	1	1	0,3588	2,9708	1	0,00500	5,94	15,56	0,92	mid blue	30,03	-4,46	-10,94
**AsB.65**	**sLPE.09**	Pers.tinc.	"Kojyoko"	Austria	sLPE	3	1	2	0,3542	2,8508	1	0,00500	5,70	15,56	0,89	mid blue	30,03	-4,46	-10,94
**AsB.65**	**sLPE.09**	Pers.tinc.	"Kojyoko"	Austria	sLPE	3	2	1	0,3702	3,3359	1	0,00517	6,45	15,56	1,00	mid blue	30,03	-4,46	-10,94
**AsB.65**	**sLPE.09**	Pers.tinc.	"Kojyoko"	Austria	sLPE	3	2	2	0,3672	3,2581	1	0,00517	6,30	15,56	0,98	mid blue	30,03	-4,46	-10,94
**AsB.65**	**sLPE.09**	Pers.tinc.	"Kojyoko"	Austria	sLPE	3	3	1	0,3744	3,4076	1	0,00516	6,60	15,56	1,03	mid blue	30,03	-4,46	-10,94
**AsB.65**	**sLPE.09**	Pers.tinc.	"Kojyoko"	Austria	sLPE	3	3	2	0,374	3,3972	1	0,00516	6,58	15,56	1,02	mid blue	30,03	-4,46	-10,94
**AsB.66**	**sLPE.10**	Pers.tinc.	"Maruba"	Austria	sLPE	1	1	1	0,3173	1,8884	1	0,00528	3,58	14,86	0,53	mid blue	30,73	-3,62	-14,09
**AsB.66**	**sLPE.10**	Pers.tinc.	"Maruba"	Austria	sLPE	1	1	2	0,3154	1,8388	1	0,00528	3,48	14,86	0,52	mid blue	30,73	-3,62	-14,09
**AsB.66**	**sLPE.10**	Pers.tinc.	"Maruba"	Austria	sLPE	1	2	1	0,3156	2,088	1	0,00505	4,13	14,86	0,61	mid blue	30,73	-3,62	-14,09
**AsB.66**	**sLPE.10**	Pers.tinc.	"Maruba"	Austria	sLPE	1	2	2	0,3195	2,1965	1	0,00505	4,35	14,86	0,65	mid blue	30,73	-3,62	-14,09
**AsB.66**	**sLPE.10**	Pers.tinc.	"Maruba"	Austria	sLPE	1	3	1	0,3466	2,6833	1	0,00524	5,12	14,86	0,76	mid blue	30,73	-3,62	-14,09
**AsB.66**	**sLPE.10**	Pers.tinc.	"Maruba"	Austria	sLPE	1	3	2	0,3455	2,6547	1	0,00524	5,07	14,86	0,75	mid blue	30,73	-3,62	-14,09
**AsB.67**	**sLPE.11**	Pers.tinc.	"Maruba"	Austria	sLPE	2	1	1	0,33	2,2196	1	0,00503	4,41	15,56	0,69	mid blue	29,18	-3,18	-15,29
**AsB.67**	**sLPE.11**	Pers.tinc.	"Maruba"	Austria	sLPE	2	1	2	0,3397	2,4726	1	0,00503	4,92	15,56	0,76	mid blue	29,18	-3,18	-15,29
**AsB.67**	**sLPE.11**	Pers.tinc.	"Maruba"	Austria	sLPE	2	2	1	0,3397	2,7589	1	0,00506	5,45	15,56	0,85	mid blue	29,18	-3,18	-15,29
**AsB.67**	**sLPE.11**	Pers.tinc.	"Maruba"	Austria	sLPE	2	2	2	0,3412	2,8007	1	0,00506	5,53	15,56	0,86	mid blue	29,18	-3,18	-15,29
**AsB.67**	**sLPE.11**	Pers.tinc.	"Maruba"	Austria	sLPE	2	3	1	0,3383	1,6256	1	0,00501	3,24	15,56	0,50	mid blue	29,18	-3,18	-15,29
**AsB.67**	**sLPE.11**	Pers.tinc.	"Maruba"	Austria	sLPE	2	3	2	0,3373	1,6076	1	0,00501	3,21	15,56	0,50	mid blue	29,18	-3,18	-15,29
**AsB.68**	**sLPE.12**	Pers.tinc.	"Maruba"	Austria	sLPE	3	1	1	0,3346	2,3396	1	0,00532	4,40	15,34	0,67	mid blue	29,76	-3,16	-14,19

**AsB.68**	**sLPE.12**	Pers.tinc.	"Maruba"	Austria	sLPE	3	1	2	0,4093	4,2879	1	0,00532	8,06	15,34	1,24	mid blue	29,76	-3,16	-14,19
**AsB.68**	**sLPE.12**	Pers.tinc.	"Maruba"	Austria	sLPE	3	2	1	0,3351	2,6308	1	0,00526	5,00	15,34	0,77	mid blue	29,76	-3,16	-14,19
**AsB.68**	**sLPE.12**	Pers.tinc.	"Maruba"	Austria	sLPE	3	2	2	0,3323	2,5529	1	0,00526	4,85	15,34	0,74	mid blue	29,76	-3,16	-14,19
**AsB.68**	**sLPE.12**	Pers.tinc.	"Maruba"	Austria	sLPE	3	3	1	0,3382	1,6238	1	0,00521	3,12	15,34	0,48	mid blue	29,76	-3,16	-14,19
**AsB.68**	**sLPE.12**	Pers.tinc.	"Maruba"	Austria	sLPE	3	3	2	0,337	1,6022	1	0,00521	3,08	15,34	0,47	mid blue	29,76	-3,16	-14,19
**AsB.72**	**sLPE.25**	Ind.arr.	Burkina Faso	Austria	sLPE	1	1	1	0,3082	1,651	1	0,00500	3,30	17,66	0,58	green	41,91	-6,63	7,77
**AsB.72**	**sLPE.25**	Ind.arr.	Burkina Faso	Austria	sLPE	1	1	2	0,3122	1,7553	1	0,00500	3,51	17,66	0,62	green	41,91	-6,63	7,77
**AsB.72**	**sLPE.25**	Ind.arr.	Burkina Faso	Austria	sLPE	1	2	1	0,3099	1,7746	1	0,00506	3,51	17,66	0,62	green	41,91	-6,63	7,77
**AsB.72**	**sLPE.25**	Ind.arr.	Burkina Faso	Austria	sLPE	1	2	2	0,308	1,7258	1	0,00506	3,41	17,66	0,60	green	41,91	-6,63	7,77
**AsB.72**	**sLPE.25**	Ind.arr.	Burkina Faso	Austria	sLPE	1	3	1	0,3107	1,1279	1	0,00507	2,22	17,66	0,39	green	41,91	-6,63	7,77
**AsB.72**	**sLPE.25**	Ind.arr.	Burkina Faso	Austria	sLPE	1	3	2	0,3127	1,164	1	0,00507	2,30	17,66	0,41	green	41,91	-6,63	7,77
**AsB.73**	**sLPE.26**	Ind.arr.	Burkina Faso	Austria	sLPE	2	1	1	0,3759	3,4168	1	0,00533	6,41	18,46	1,18	green	41,92	-6,11	8,78
**AsB.73**	**sLPE.26**	Ind.arr.	Burkina Faso	Austria	sLPE	2	1	2	0,3803	3,5316	1	0,00533	6,63	18,46	1,22	green	41,92	-6,11	8,78
**AsB.73**	**sLPE.26**	Ind.arr.	Burkina Faso	Austria	sLPE	2	2	1	0,3217	2,0779	1	0,00516	4,03	18,46	0,74	green	41,92	-6,11	8,78
**AsB.73**	**sLPE.26**	Ind.arr.	Burkina Faso	Austria	sLPE	2	2	2	0,3198	2,029	1	0,00516	3,93	18,46	0,73	green	41,92	-6,11	8,78
**AsB.73**	**sLPE.26**	Ind.arr.	Burkina Faso	Austria	sLPE	2	3	1	0,3223	1,3371	1	0,00515	2,60	18,46	0,48	green	41,92	-6,11	8,78
**AsB.73**	**sLPE.26**	Ind.arr.	Burkina Faso	Austria	sLPE	2	3	2	0,328	1,4399	1	0,00515	2,80	18,46	0,52	green	41,92	-6,11	8,78
**AsB.74**	**sLPE.27**	Ind.arr.	Burkina Faso	Austria	sLPE	3	1	1	0,3281	2,1701	1	0,00529	4,10	17,30	0,71	green	37,43	-7,06	2,43
**AsB.74**	**sLPE.27**	Ind.arr.	Burkina Faso	Austria	sLPE	3	1	2	0,3251	2,0918	1	0,00529	3,95	17,30	0,68	green	37,43	-7,06	2,43
**AsB.74**	**sLPE.27**	Ind.arr.	Burkina Faso	Austria	sLPE	3	2	1	0,3723	3,3783	1	0,00508	6,65	17,30	1,15	green	37,43	-7,06	2,43
**AsB.74**	**sLPE.27**	Ind.arr.	Burkina Faso	Austria	sLPE	3	2	2	0,3755	3,4605	1	0,00508	6,81	17,30	1,18	green	37,43	-7,06	2,43
**AsB.74**	**sLPE.27**	Ind.arr.	Burkina Faso	Austria	sLPE	3	3	1	0,377	2,3235	1	0,00504	4,61	17,30	0,80	green	37,43	-7,06	2,43
**AsB.74**	**sLPE.27**	Ind.arr.	Burkina Faso	Austria	sLPE	3	3	2	0,3773	2,3289	1	0,00504	4,62	17,30	0,80	green	37,43	-7,06	2,43
**AsB.69**	**sLPE.19**	Ind.arr.	unknown	Austria	sLPE	1	1	1	0,4218	4,614	1	0,00503	9,17	18,88	1,73	greenish blue	26,41	-6,75	7,11
**AsB.69**	**sLPE.19**	Ind.arr.	unknown	Austria	sLPE	1	1	2	0,3939	3,8863	1	0,00503	7,73	18,88	1,46	greenish blue	26,41	-6,75	7,11
**AsB.69**	**sLPE.19**	Ind.arr.	unknown	Austria	sLPE	1	2	1	0,3999	4,4349	1	0,00524	8,46	18,88	1,60	greenish blue	26,41	-6,75	7,11
**AsB.69**	**sLPE.19**	Ind.arr.	unknown	Austria	sLPE	1	2	2	0,4016	4,4822	1	0,00524	8,55	18,88	1,61	greenish blue	26,41	-6,75	7,11
**AsB.69**	**sLPE.19**	Ind.arr.	unknown	Austria	sLPE	1	3	1	0,41	2,9186	1	0,00515	5,67	18,88	1,07	greenish blue	26,41	-6,75	7,11
**AsB.69**	**sLPE.19**	Ind.arr.	unknown	Austria	sLPE	1	3	2	0,4114	2,9438	1	0,00515	5,72	18,88	1,08	greenish blue	26,41	-6,75	7,11
**AsB.70**	**sLPE.20**	Ind.arr.	unknown	Austria	sLPE	2	1	1	0,4321	4,8826	1	0,00511	9,55	19,24	1,84	greenish blue	24,06	-5,27	-10,11
**AsB.70**	**sLPE.20**	Ind.arr.	unknown	Austria	sLPE	2	1	2	0,4383	5,0443	1	0,00511	9,87	19,24	1,90	greenish blue	24,06	-5,27	-10,11
**AsB.70**	**sLPE.20**	Ind.arr.	unknown	Austria	sLPE	2	2	1	0,4402	5,5568	1	0,00536	10,37	19,24	1,99	greenish blue	24,06	-5,27	-10,11
**AsB.70**	**sLPE.20**	Ind.arr.	unknown	Austria	sLPE	2	2	2	0,443	5,6347	1	0,00536	10,51	19,24	2,02	greenish blue	24,06	-5,27	-10,11
**AsB.70**	**sLPE.20**	Ind.arr.	unknown	Austria	sLPE	2	3	1	0,4419	3,4938	1	0,00509	6,86	19,24	1,32	greenish blue	24,06	-5,27	-10,11
**AsB.70**	**sLPE.20**	Ind.arr.	unknown	Austria	sLPE	2	3	2	0,4464	3,575	1	0,00509	7,02	19,24	1,35	greenish blue	24,06	-5,27	-10,11
**AsB.71**	**sLPE.21**	Ind.arr.	unknown	Austria	sLPE	3	1	1	0,4252	4,7027	1	0,00511	9,20	19,72	1,81	greenish blue	24,93	-5,79	-8,61
**AsB.71**	**sLPE.21**	Ind.arr.	unknown	Austria	sLPE	3	1	2	0,4285	4,7887	1	0,00511	9,37	19,72	1,85	greenish blue	24,93	-5,79	-8,61
**AsB.71**	**sLPE.21**	Ind.arr.	unknown	Austria	sLPE	3	2	1	0,4174	4,5374	1	0,00507	8,95	19,72	1,76	greenish blue	24,93	-5,79	-8,61
**AsB.71**	**sLPE.21**	Ind.arr.	unknown	Austria	sLPE	3	2	2	0,4171	4,5297	1	0,00507	8,93	19,72	1,76	greenish blue	24,93	-5,79	-8,61
**AsB.71**	**sLPE.21**	Ind.arr.	unknown	Austria	sLPE	3	3	1	0,4252	3,1927	1	0,00505	6,32	19,72	1,25	greenish blue	24,93	-5,79	-8,61
**AsB.71**	**sLPE.21**	Ind.arr.	unknown	Austria	sLPE	3	3	2	0,4286	3,254	1	0,00505	6,44	19,72	1,27	greenish blue	24,93	-5,79	-8,61
**AsB.75**	**sLPE.28**	Ind.arr.	unknown	Austria	mod.sLPE	1	1	1	0,3681	3,6121	1	0,00528	6,84	21,92	1,50	mid blue	25,8	-3,58	-9,15
**AsB.75**	**sLPE.28**	Ind.arr.	unknown	Austria	mod.sLPE	1	1	2	0,2943	1,5376	1	0,00528	2,91	21,92	0,64	mid blue	25,8	-3,58	-9,15
**AsB.75**	**sLPE.28**	Ind.arr.	unknown	Austria	mod.sLPE	1	2	1	0,3627	3,1316	1	0,00520	6,02	21,92	1,32	mid blue	25,8	-3,58	-9,15
**AsB.75**	**sLPE.28**	Ind.arr.	unknown	Austria	mod.sLPE	1	2	2	0,364	3,165	1	0,00520	6,09	21,92	1,33	mid blue	25,8	-3,58	-9,15
**AsB.75**	**sLPE.28**	Ind.arr.	unknown	Austria	mod.sLPE	1	3	1	0,3829	2,4299	1	0,00525	4,63	21,92	1,01	mid blue	25,8	-3,58	-9,15
**AsB.75**	**sLPE.28**	Ind.arr.	unknown	Austria	mod.sLPE	1	3	2	0,3812	2,3992	1	0,00525	4,57	21,92	1,00	mid blue	25,8	-3,58	-9,15
**AsB.76**	**sLPE.29**	Ind.arr.	unknown	Austria	mod.sLPE	2	1	1	0,364	3,4968	1	0,00500	6,99	22,82	1,60	mid blue	27,5	-3,94	-8,56
**AsB.76**	**sLPE.29**	Ind.arr.	unknown	Austria	mod.sLPE	2	1	2	0,3747	3,7976	1	0,00500	7,60	22,82	1,73	mid blue	27,5	-3,94	-8,56
**AsB.76**	**sLPE.29**	Ind.arr.	unknown	Austria	mod.sLPE	2	2	1	0,3727	3,3886	1	0,00518	6,54	22,82	1,49	mid blue	27,5	-3,94	-8,56
**AsB.76**	**sLPE.29**	Ind.arr.	unknown	Austria	mod.sLPE	2	2	2	0,3779	3,5222	1	0,00518	6,80	22,82	1,55	mid blue	27,5	-3,94	-8,56
**AsB.76**	**sLPE.29**	Ind.arr.	unknown	Austria	mod.sLPE	2	3	1	0,3792	2,3632	1	0,00501	4,72	22,82	1,08	mid blue	27,5	-3,94	-8,56
**AsB.76**	**sLPE.29**	Ind.arr.	unknown	Austria	mod.sLPE	2	3	2	0,378	2,3415	1	0,00501	4,67	22,82	1,07	mid blue	27,5	-3,94	-8,56
**AsB.77**	**sLPE.30**	Ind.arr.	unknown	Austria	mod.sLPE	3	1	1	0,3874	4,1546	1	0,00505	8,23	23,02	1,89	mid blue	25,42	-3,89	-10,55
**AsB.77**	**sLPE.30**	Ind.arr.	unknown	Austria	mod.sLPE	3	1	2	0,4055	4,6634	1	0,00505	9,23	23,02	2,13	mid blue	25,42	-3,89	-10,55
**AsB.77**	**sLPE.30**	Ind.arr.	unknown	Austria	mod.sLPE	3	2	1	0,3965	4,0003	1	0,00526	7,61	23,02	1,75	mid blue	25,42	-3,89	-10,55
**AsB.77**	**sLPE.30**	Ind.arr.	unknown	Austria	mod.sLPE	3	2	2	0,3966	4,0028	1	0,00526	7,61	23,02	1,75	mid blue	25,42	-3,89	-10,55
**AsB.77**	**sLPE.30**	Ind.arr.	unknown	Austria	mod.sLPE	3	3	1	0,3871	3,4273	1	0,00505	6,79	23,02	1,56	mid blue	25,42	-3,89	-10,55
**AsB.77**	**sLPE.30**	Ind.arr.	unknown	Austria	mod.sLPE	3	3	2	0,3873	3,4323	1	0,00505	6,80	23,02	1,56	mid blue	25,42	-3,89	-10,55
**Indigo Standard**																	10,7	5,33	-7,49
