# Supplementary material for: “Purplish Blue” or “Greenish Grey”? Indigo Qualities and Extraction Yields from Six Species
Source: Plants (Basel). 2024 Mar 22;13(7):918. doi: 10.3390/plants13070918 (PMC11013892; doi:10.3390/plants13070918)
Supplement: Supplementary file 1 [file plants-13-00918-s001.zip › Table S2.pdf]

Table S2. Raw data of samples from local practice extractions (LPE, ADD)

AsB-No.	Sample code	Species	Provenance	Country	Extraction	Sample preparation	Duplicates	Raw data (= Absorp-tion)	Single conc. (µg/ml)	Dilution factor	Indigo weight (g)	Indigotin (%)	**Colour classification of ground indigo:** dark blue / violet<br>dark blue / black<br>dark blue<br>mid blue<br>light blue<br>grey blue<br>greenish blue<br>green	L*	a*	b*
**AsB.15**	**LPE.03**	Stro.cus.	Xin Cun	China	with lime	1	1	0,345	2,3395	1	0,00510	4,59	mid blue	28,47	-4,17	-12,57
**AsB.15**	**LPE.03**	Stro.cus.	Xin Cun	China	with lime	1	2	0,3409	2,2398	1	0,00510	4,39	mid blue	28,47	-4,17	-12,57
**AsB.15**	**LPE.03**	Stro.cus.	Xin Cun	China	with lime	2	1	0,3252	2,4252	1	0,00518	4,68	mid blue	28,47	-4,17	-12,57
**AsB.15**	**LPE.03**	Stro.cus.	Xin Cun	China	with lime	2	2	0,326	2,4489	1	0,00518	4,73	mid blue	28,47	-4,17	-12,57
**AsB.15**	**LPE.03**	Stro.cus.	Xin Cun	China	with lime	3	1	0,3281	2,2024	1	0,00516	4,27	mid blue	28,47	-4,17	-12,57
**AsB.15**	**LPE.03**	Stro.cus.	Xin Cun	China	with lime	3	2	0,3289	2,2239	1	0,00516	4,31	mid blue	28,47	-4,17	-12,57
**AsB.16**	**LPE.04**	Wri.laev.	Xin Cun	China	with lime	1	1	0,3004	1,2549	1	0,00526	2,39	light blue	38,01	-6,21	-13,84
**AsB.16**	**LPE.04**	Wri.laev.	Xin Cun	China	with lime	1	2	0,2926	1,0652	1	0,00526	2,03	light blue	38,01	-6,21	-13,84
**AsB.16**	**LPE.04**	Wri.laev.	Xin Cun	China	with lime	2	1	0,2716	0,83704	1	0,00524	1,60	light blue	38,01	-6,21	-13,84
**AsB.16**	**LPE.04**	Wri.laev.	Xin Cun	China	with lime	2	2	0,2718	0,84296	1	0,00524	1,61	light blue	38,01	-6,21	-13,84
**AsB.16**	**LPE.04**	Wri.laev.	Xin Cun	China	with lime	3	1	0,2742	0,7556	1	0,00510	1,48	light blue	38,01	-6,21	-13,84
**AsB.16**	**LPE.04**	Wri.laev.	Xin Cun	China	with lime	3	2	0,2738	0,74487	1	0,00510	1,46	light blue	38,01	-6,21	-13,84
**AsB.98**	**LPE.05**	Ind.tinc.	Yu Dao	China	with lime	1	1	0,302	1,5815	1	0,00506	3,13	mid blue	28,11	-3,47	-5,21
**AsB.98**	**LPE.05**	Ind.tinc.	Yu Dao	China	with lime	1	2	0,2903	1,2955	1	0,00506	2,56	mid blue	28,11	-3,47	-5,21
**AsB.98**	**LPE.05**	Ind.tinc.	Yu Dao	China	with lime	2	1	0,2785	1,0397	1	0,00501	2,08	mid blue	28,11	-3,47	-5,21
**AsB.98**	**LPE.05**	Ind.tinc.	Yu Dao	China	with lime	2	2	0,2763	0,99007	1	0,00501	1,98	mid blue	28,11	-3,47	-5,21
**AsB.98**	**LPE.05**	Ind.tinc.	Yu Dao	China	with lime	3	1	0,2893	1,0644	1	0,00531	2,00	mid blue	28,11	-3,47	-5,21
**AsB.98**	**LPE.05**	Ind.tinc.	Yu Dao	China	with lime	3	2	0,2904	1,0931	1	0,00531	2,06	mid blue	28,11	-3,47	-5,21
**AsB.17**	**ADD.01**	Stro.cus.	Xin Cun	China	with lime	1	1	0,2909	1,3102	1	0,00525	1,90	mid blue	29,32	-3,43	-9,69
**AsB.17**	**ADD.01**	Stro.cus.	Xin Cun	China	with lime	1	2	0,2914	1,3224	1	0,00525	1,90	mid blue	29,32	-3,43	-9,69
**AsB.17**	**ADD.01**	Stro.cus.	Xin Cun	China	with lime	2	1	0,2834	1,1504	1	0,00525	2,19	mid blue	29,32	-3,43	-9,69
**AsB.17**	**ADD.01**	Stro.cus.	Xin Cun	China	with lime	2	2	0,2813	1,103	1	0,00525	2,10	mid blue	29,32	-3,43	-9,69
**AsB.17**	**ADD.01**	Stro.cus.	Xin Cun	China	with lime	3	1	0,3018	1,3909	1	0,00502	2,77	mid blue	29,32	-3,43	-9,69
**AsB.17**	**ADD.01**	Stro.cus.	Xin Cun	China	with lime	3	2	0,3012	1,3752	1	0,00502	2,74	mid blue	29,32	-3,43	-9,69
**AsB.18**	**ADD.02**	Stro.cus.	Xin Cun	China	with lime	1	1	0,3417	2,2592	1	0,00502	4,50	dark blue / black	17,25	-0,59	-2,81
**AsB.18**	**ADD.02**	Stro.cus.	Xin Cun	China	with lime	1	2	0,3444	2,3249	1	0,00502	4,63	dark blue / black	17,25	-0,59	-2,81
**AsB.18**	**ADD.02**	Stro.cus.	Xin Cun	China	with lime	2	1	0,3443	2,9911	1	0,00516	5,80	dark blue / black	17,25	-0,59	-2,81
**AsB.18**	**ADD.02**	Stro.cus.	Xin Cun	China	with lime	2	2	0,3473	3,08	1	0,00516	5,97	dark blue / black	17,25	-0,59	-2,81
**AsB.18**	**ADD.02**	Stro.cus.	Xin Cun	China	with lime	3	1	0,3503	2,7983	1	0,00519	5,39	dark blue / black	17,25	-0,59	-2,81
**AsB.18**	**ADD.02**	Stro.cus.	Xin Cun	China	with lime	3	2	0,3498	2,7849	1	0,00519	5,37	dark blue / black	17,25	-0,59	-2,81
**AsB.19**	**ADD.05**	Ind.tinc.	Yu Long	China	with lime	1	1	0,3538	2,5535	1	0,00512	4,99	dark blue	16,07	-1,13	-7,13
**AsB.19**	**ADD.05**	Ind.tinc.	Yu Long	China	with lime	1	2	0,3547	2,5754	1	0,00512	5,03	dark blue	16,07	-1,13	-7,13
**AsB.19**	**ADD.05**	Ind.tinc.	Yu Long	China	with lime	2	1	0,3542	3,2844	1	0,00511	6,43	dark blue	16,07	-1,13	-7,13
**AsB.19**	**ADD.05**	Ind.tinc.	Yu Long	China	with lime	2	2	0,3589	3,4237	1	0,00511	6,70	dark blue	16,07	-1,13	-7,13
**AsB.19**	**ADD.05**	Ind.tinc.	Yu Long	China	with lime	3	1	0,3671	3,2618	1	0,00517	6,31	dark blue	16,07	-1,13	-7,13
**AsB.19**	**ADD.05**	Ind.tinc.	Yu Long	China	with lime	3	2	0,3628	3,1471	1	0,00517	6,09	dark blue	16,07	-1,13	-7,13
**AsB.20**	**ADD.06**	Ind.tinc.	Yu Long	China	with lime	1	1	0,372	2,9961	1	0,00525	5,71	dark blue	17,72	-1,56	-6,31
**AsB.20**	**ADD.06**	Ind.tinc.	Yu Long	China	with lime	1	2	0,3639	2,7991	1	0,00525	5,33	dark blue	17,72	-1,56	-6,31
**AsB.20**	**ADD.06**	Ind.tinc.	Yu Long	China	with lime	2	1	0,3554	3,32	1	0,00511	6,50	dark blue	17,72	-1,56	-6,31
**AsB.20**	**ADD.06**	Ind.tinc.	Yu Long	China	with lime	2	2	0,3506	3,1778	1	0,00511	6,22	dark blue	17,72	-1,56	-6,31
**AsB.20**	**ADD.06**	Ind.tinc.	Yu Long	China	with lime	3	1	0,3564	2,9764	1	0,00511	5,82	dark blue	17,72	-1,56	-6,31
**AsB.20**	**ADD.06**	Ind.tinc.	Yu Long	China	with lime	3	2	0,3567	2,9844	1	0,00511	5,84	dark blue	17,72	-1,56	-6,31
**AsB.21**	**ADD.07**	Ind.tinc.	Yu Long	China	with lime	1	1	0,3552	2,5875	1	0,00517	5,00	dark blue	17,6	-1,41	-6,14

**AsB.21**	**ADD.07**	Ind.tinc.	Yu Long	China	with lime	1	2	0,35	2,4611	1	0,00517	4,76	dark blue	17,6	-1,41	-6,14
**AsB.21**	**ADD.07**	Ind.tinc.	Yu Long	China	with lime	2	1	0,3271	2,4815	1	0,00502	4,94	dark blue	17,6	-1,41	-6,14
**AsB.21**	**ADD.07**	Ind.tinc.	Yu Long	China	with lime	2	2	0,3268	2,4726	1	0,00502	4,93	dark blue	17,6	-1,41	-6,14
**AsB.21**	**ADD.07**	Ind.tinc.	Yu Long	China	with lime	3	1	0,3109	2,2973	1	0,00512	4,49	dark blue	17,6	-1,41	-6,14
**AsB.21**	**ADD.07**	Ind.tinc.	Yu Long	China	with lime	3	2	0,3162	2,4352	1	0,00512	4,76	dark blue	17,6	-1,41	-6,14
**AsB.22**	**ADD.09**	Ind.tinc.	Yu Long	China	with lime	1	1	0,3433	2,2982	1	0,00517	4,45	dark blue	19,51	-2,41	-7,12
**AsB.22**	**ADD.09**	Ind.tinc.	Yu Long	China	with lime	1	2	0,3485	2,4246	1	0,00517	4,69	dark blue	19,51	-2,41	-7,12
**AsB.22**	**ADD.09**	Ind.tinc.	Yu Long	China	with lime	2	1	0,3151	2,5822	1	0,00518	4,98	dark blue	19,51	-2,41	-7,12
**AsB.22**	**ADD.09**	Ind.tinc.	Yu Long	China	with lime	2	2	0,3085	2,405	1	0,00518	4,64	dark blue	19,51	-2,41	-7,12
**AsB.22**	**ADD.09**	Ind.tinc.	Yu Long	China	with lime	3	1	0,3112	2,3051	1	0,00508	4,54	dark blue	19,51	-2,41	-7,12
**AsB.22**	**ADD.09**	Ind.tinc.	Yu Long	China	with lime	3	2	0,3087	2,2401	1	0,00508	4,41	dark blue	19,51	-2,41	-7,12
**AsB.23**	**ADD.10**	Ind.tinc.	Yu Long	China	with lime	1	1	0,3156	1,4076	1	0,00536	2,63	mid blue	28,43	-3,18	-6,41
**AsB.23**	**ADD.10**	Ind.tinc.	Yu Long	China	with lime	1	2	0,3193	1,5054	1	0,00536	2,81	mid blue	28,43	-3,18	-6,41
**AsB.23**	**ADD.10**	Ind.tinc.	Yu Long	China	with lime	2	1	0,2815	1,6802	1	0,00511	3,29	mid blue	28,43	-3,18	-6,41
**AsB.23**	**ADD.10**	Ind.tinc.	Yu Long	China	with lime	2	2	0,2847	1,7661	1	0,00511	3,46	mid blue	28,43	-3,18	-6,41
**AsB.23**	**ADD.10**	Ind.tinc.	Yu Long	China	with lime	3	1	0,2769	1,4129	1	0,00508	2,78	mid blue	28,43	-3,18	-6,41
**AsB.23**	**ADD.10**	Ind.tinc.	Yu Long	China	with lime	3	2	0,2761	1,3921	1	0,00508	2,74	mid blue	28,43	-3,18	-6,41
**AsB.81**	**ADD.13**	Ind.tinc.	Simalta	India	first without lime	1	1	0,6802	12,143	1	0,00515	23,58	dark blue / violet	13,78	0,63	-5,85
**AsB.81**	**ADD.13**	Ind.tinc.	Simalta	India	first without lime	1	2	0,6702	11,87	1	0,00515	23,05	dark blue / violet	13,78	0,63	-5,85
**AsB.81**	**ADD.13**	Ind.tinc.	Simalta	India	first without lime	2	1	0,7505	13,076	1	0,00529	24,72	dark blue / violet	13,78	0,63	-5,85
**AsB.81**	**ADD.13**	Ind.tinc.	Simalta	India	first without lime	2	2	0,7123	12,102	1	0,00529	22,88	dark blue / violet	13,78	0,63	-5,85
**AsB.81**	**ADD.13**	Ind.tinc.	Simalta	India	first without lime	3	1	0,7121	11,618	1	0,00523	22,21	dark blue / violet	13,78	0,63	-5,85
**AsB.81**	**ADD.13**	Ind.tinc.	Simalta	India	first without lime	3	2	0,7036	11,399	1	0,00523	21,80	dark blue / violet	13,78	0,63	-5,85
**AsB.82**	**ADD.14**	Ind.tinc.	Simalta	India	second with lime	1	1	0,4035	4,9696	1	0,00509	9,76	mid blue	21,09	-2,54	-7,33
**AsB.82**	**ADD.14**	Ind.tinc.	Simalta	India	second with lime	1	2	0,4021	4,9341	1	0,00509	9,69	mid blue	21,09	-2,54	-7,33
**AsB.82**	**ADD.14**	Ind.tinc.	Simalta	India	second with lime	2	1	0,4602	5,6374	1	0,00526	10,72	mid blue	21,09	-2,54	-7,33
**AsB.82**	**ADD.14**	Ind.tinc.	Simalta	India	second with lime	2	2	0,4447	5,239	1	0,00526	9,96	mid blue	21,09	-2,54	-7,33
**AsB.82**	**ADD.14**	Ind.tinc.	Simalta	India	second with lime	3	1	0,4311	4,5301	1	0,00526	8,61	mid blue	21,09	-2,54	-7,33
**AsB.82**	**ADD.14**	Ind.tinc.	Simalta	India	second with lime	3	2	0,4366	4,6679	1	0,00526	8,87	mid blue	21,09	-2,54	-7,33
**AsB.83**	**ADD.15**	Ind.tinc.	Digoli	India	first without lime	1	1	0,6195	10,483	1	0,00515	20,36	dark blue	17,9	-1,9	-5,21
**AsB.83**	**ADD.15**	Ind.tinc.	Digoli	India	first without lime	1	2	0,6177	10,434	1	0,00515	20,26	dark blue	17,9	-1,9	-5,21
**AsB.83**	**ADD.15**	Ind.tinc.	Digoli	India	first without lime	2	1	0,6801	11,28	1	0,00506	22,29	dark blue	17,9	-1,9	-5,21
**AsB.83**	**ADD.15**	Ind.tinc.	Digoli	India	first without lime	2	2	0,6663	10,928	1	0,00506	21,60	dark blue	17,9	-1,9	-5,21
**AsB.83**	**ADD.15**	Ind.tinc.	Digoli	India	first without lime	3	1	0,6672	10,46	1	0,00514	20,35	dark blue	17,9	-1,9	-5,21
**AsB.83**	**ADD.15**	Ind.tinc.	Digoli	India	first without lime	3	2	0,6553	10,153	1	0,00514	19,75	dark blue	17,9	-1,9	-5,21
**AsB.84**	**ADD.16**	Ind.tinc.	Digoli	India	second with lime	1	1	0,4041	4,9848	1	0,00526	9,48	mid blue	17,96	-1,52	-3,86
**AsB.84**	**ADD.16**	Ind.tinc.	Digoli	India	second with lime	1	2	0,4025	4,9442	1	0,00526	9,40	mid blue	17,96	-1,52	-3,86
**AsB.84**	**ADD.16**	Ind.tinc.	Digoli	India	second with lime	2	1	0,4014	4,1136	1	0,00515	7,99	mid blue	17,96	-1,52	-3,86
**AsB.84**	**ADD.16**	Ind.tinc.	Digoli	India	second with lime	2	2	0,3941	3,9326	1	0,00515	7,64	mid blue	17,96	-1,52	-3,86
**AsB.84**	**ADD.16**	Ind.tinc.	Digoli	India	second with lime	3	1	0,4243	4,3596	1	0,00507	8,60	mid blue	17,96	-1,52	-3,86
**AsB.84**	**ADD.16**	Ind.tinc.	Digoli	India	second with lime	3	2	0,4252	4,3822	1	0,00507	8,64	mid blue	17,96	-1,52	-3,86
**AsB.85**	**ADD.17**	Ind.tinc.	Thanga	India	first without lime	1	1	0,351	3,6381	1	0,00520	7,00	grey blue	24,58	-1,93	-0,27
**AsB.85**	**ADD.17**	Ind.tinc.	Thanga	India	first without lime	1	2	0,3561	3,7674	1	0,00520	7,25	grey blue	24,58	-1,93	-0,27
**AsB.85**	**ADD.17**	Ind.tinc.	Thanga	India	first without lime	2	1	0,3563	2,9953	1	0,00532	5,63	grey blue	24,58	-1,93	-0,27
**AsB.85**	**ADD.17**	Ind.tinc.	Thanga	India	first without lime	2	2	0,3709	3,3573	1	0,00532	6,31	grey blue	24,58	-1,93	-0,27
**AsB.85**	**ADD.17**	Ind.tinc.	Thanga	India	first without lime	3	1	0,3857	3,3922	1	0,00515	6,59	grey blue	24,58	-1,93	-0,27
**AsB.85**	**ADD.17**	Ind.tinc.	Thanga	India	first without lime	3	2	0,3827	3,317	1	0,00515	6,44	grey blue	24,58	-1,93	-0,27
**AsB.86**	**ADD.18**	Ind.tinc.	Thanga	India	second with lime	1	1	0,2953	2,2255	1	0,00510	4,36	grey blue	23,46	-1,34	0,69
**AsB.86**	**ADD.18**	Ind.tinc.	Thanga	India	second with lime	1	2	0,3061	2,4994	1	0,00510	4,90	grey blue	23,46	-1,34	0,69
**AsB.86**	**ADD.18**	Ind.tinc.	Thanga	India	second with lime	2	1	0,3127	1,9142	1	0,00502	3,81	grey blue	23,46	-1,34	0,69
**AsB.86**	**ADD.18**	Ind.tinc.	Thanga	India	second with lime	2	2	0,3072	1,7778	1	0,00502	3,54	grey blue	23,46	-1,34	0,69
**AsB.86**	**ADD.18**	Ind.tinc.	Thanga	India	second with lime	3	1	0,3211	1,7732	1	0,00515	3,44	grey blue	23,46	-1,34	0,69
**AsB.86**	**ADD.18**	Ind.tinc.	Thanga	India	second with lime	3	2	0,3244	1,8559	1	0,00515	3,60	grey blue	23,46	-1,34	0,69
**AsB.87**	**ADD.19**	Ind.tinc.	Chankana	India	first without lime	1	1	0,312	2,649	1	0,00524	5,06	mid blue	24,83	-3,43	-8,21

**AsB.87**	**ADD.19**	Ind.tinc.	Chankana	India	first without lime	1	2	0,3111	2,6262	1	0,00524	5,01	mid blue	24,83	-3,43	-8,21
**AsB.87**	**ADD.19**	Ind.tinc.	Chankana	India	first without lime	2	1	0,3115	1,8845	1	0,00526	3,58	mid blue	24,83	-3,43	-8,21
**AsB.87**	**ADD.19**	Ind.tinc.	Chankana	India	first without lime	2	2	0,3134	1,9316	1	0,00526	3,67	mid blue	24,83	-3,43	-8,21
**AsB.87**	**ADD.19**	Ind.tinc.	Chankana	India	first without lime	3	1	0,3488	2,4674	1	0,00525	4,70	mid blue	24,83	-3,43	-8,21
**AsB.87**	**ADD.19**	Ind.tinc.	Chankana	India	first without lime	3	2	0,3486	2,4624	1	0,00525	4,69	mid blue	24,83	-3,43	-8,21
**AsB.88**	**ADD.20**	Ind.tinc.	Chankana	India	second with lime	1	1	0,2958	2,2381	1	0,00527	4,25	mid blue	23,87	-3,33	-5,74
**AsB.88**	**ADD.20**	Ind.tinc.	Chankana	India	second with lime	1	2	0,2971	2,2711	1	0,00527	4,31	mid blue	23,87	-3,33	-5,74
**AsB.88**	**ADD.20**	Ind.tinc.	Chankana	India	second with lime	2	1	0,3096	1,8373	1	0,00520	3,53	mid blue	23,87	-3,33	-5,74
**AsB.88**	**ADD.20**	Ind.tinc.	Chankana	India	second with lime	2	2	0,3117	1,8894	1	0,00520	3,63	mid blue	23,87	-3,33	-5,74
**AsB.88**	**ADD.20**	Ind.tinc.	Chankana	India	second with lime	3	1	0,3293	1,9787	1	0,00519	3,81	mid blue	23,87	-3,33	-5,74
**AsB.88**	**ADD.20**	Ind.tinc.	Chankana	India	second with lime	3	2	0,3294	1,9812	1	0,00519	3,82	mid blue	23,87	-3,33	-5,74
**AsB.89**	**ADD.21**	Stro.cus.	Simalta	India	without lime	1	1	0,5755	9,3317	1	0,00501	18,63	dark blue / black	16,45	-1,21	-3,98
**AsB.89**	**ADD.21**	Stro.cus.	Simalta	India	without lime	1	2	0,5668	9,1111	1	0,00501	18,19	dark blue / black	16,45	-1,21	-3,98
**AsB.89**	**ADD.21**	Stro.cus.	Simalta	India	without lime	2	1	0,5714	8,3288	1	0,00516	16,14	dark blue / black	16,45	-1,21	-3,98
**AsB.89**	**ADD.21**	Stro.cus.	Simalta	India	without lime	2	2	0,5752	8,423	1	0,00516	16,32	dark blue / black	16,45	-1,21	-3,98
**AsB.89**	**ADD.21**	Stro.cus.	Simalta	India	without lime	3	1	0,6193	9,2469	1	0,00515	17,96	dark blue / black	16,45	-1,21	-3,98
**AsB.89**	**ADD.21**	Stro.cus.	Simalta	India	without lime	3	2	0,6283	9,4724	1	0,00515	18,39	dark blue / black	16,45	-1,21	-3,98
**AsB.90**	**ADD.22**	Stro.cus.	Tripuradevi	India	without lime	1	1	0,929	18,948	1	0,00501	37,82	dark blue / violet	12,79	0,38	-9,41
**AsB.90**	**ADD.22**	Stro.cus.	Tripuradevi	India	without lime	1	2	0,9349	19,11	1	0,00501	38,14	dark blue / violet	12,79	0,38	-9,41
**AsB.90**	**ADD.22**	Stro.cus.	Tripuradevi	India	without lime	2	1	0,9197	18,201	1	0,00511	35,62	dark blue / violet	12,79	0,38	-9,41
**AsB.90**	**ADD.22**	Stro.cus.	Tripuradevi	India	without lime	2	2	0,9156	18,082	1	0,00511	35,39	dark blue / violet	12,79	0,38	-9,41
**AsB.90**	**ADD.22**	Stro.cus.	Tripuradevi	India	without lime	3	1	0,9489	17,661	1	0,00507	34,83	dark blue / violet	12,79	0,38	-9,41
**AsB.90**	**ADD.22**	Stro.cus.	Tripuradevi	India	without lime	3	2	0,9251	17,087	1	0,00507	33,70	dark blue / violet	12,79	0,38	-9,41
**AsB.91**	**ADD.23**	Stro.cus.	Dharamghar	India	first without lime	1	1	0,5994	9,6833	1	0,00517	18,73	dark blue / black	13,89	-0,33	-2,71
**AsB.91**	**ADD.23**	Stro.cus.	Dharamghar	India	first without lime	1	2	0,5963	9,6063	1	0,00517	18,58	dark blue / black	13,89	-0,33	-2,71
**AsB.91**	**ADD.23**	Stro.cus.	Dharamghar	India	first without lime	2	1	0,6247	9,6504	1	0,00530	18,21	dark blue / black	13,89	-0,33	-2,71
**AsB.91**	**ADD.23**	Stro.cus.	Dharamghar	India	first without lime	2	2	0,6309	9,8041	1	0,00530	18,50	dark blue / black	13,89	-0,33	-2,71
**AsB.91**	**ADD.23**	Stro.cus.	Dharamghar	India	first without lime	3	1	0,6197	9,6944	1	0,00501	19,35	dark blue / black	13,89	-0,33	-2,71
**AsB.91**	**ADD.23**	Stro.cus.	Dharamghar	India	first without lime	3	2	0,626	9,859	1	0,00501	19,68	dark blue / black	13,89	-0,33	-2,71
**AsB.92**	**ADD.24**	Stro.cus.	Dharamghar	India	second with lime	1	1	0,3982	4,6851	1	0,00520	9,01	dark blue / black	16,35	-0,63	-1,82
**AsB.92**	**ADD.24**	Stro.cus.	Dharamghar	India	second with lime	1	2	0,3939	4,5783	1	0,00520	8,80	dark blue / black	16,35	-0,63	-1,82
**AsB.92**	**ADD.24**	Stro.cus.	Dharamghar	India	second with lime	2	1	0,4127	4,3938	1	0,00527	8,34	dark blue / black	16,35	-0,63	-1,82
**AsB.92**	**ADD.24**	Stro.cus.	Dharamghar	India	second with lime	2	2	0,4237	4,6665	1	0,00527	8,85	dark blue / black	16,35	-0,63	-1,82
**AsB.92**	**ADD.24**	Stro.cus.	Dharamghar	India	second with lime	3	1	0,4304	4,7499	1	0,00511	9,30	dark blue / black	16,35	-0,63	-1,82
**AsB.92**	**ADD.24**	Stro.cus.	Dharamghar	India	second with lime	3	2	0,4371	4,9249	1	0,00511	9,64	dark blue / black	16,35	-0,63	-1,82
**AsB.93**	**ADD.25**	Stro.cus.	Temangung	Indonesia	with lime	1	1	0,3142	2,5984	1	0,00511	5,08	mid blue	28,82	-5,26	-12,64
**AsB.93**	**ADD.25**	Stro.cus.	Temangung	Indonesia	with lime	1	2	0,3219	2,7897	1	0,00511	5,46	mid blue	28,82	-5,26	-12,64
**AsB.93**	**ADD.25**	Stro.cus.	Temangung	Indonesia	with lime	2	1	0,3453	2,7225	1	0,00532	5,12	mid blue	28,82	-5,26	-12,64
**AsB.93**	**ADD.25**	Stro.cus.	Temangung	Indonesia	with lime	2	2	0,3425	2,6531	1	0,00532	4,99	mid blue	28,82	-5,26	-12,64
**AsB.93**	**ADD.25**	Stro.cus.	Temangung	Indonesia	with lime	3	1	0,3533	2,7361	1	0,00523	5,23	mid blue	28,82	-5,26	-12,64
**AsB.93**	**ADD.25**	Stro.cus.	Temangung	Indonesia	with lime	3	2	0,3568	2,8275	1	0,00523	5,41	mid blue	28,82	-5,26	-12,64
**AsB.94**	**ADD.26**	Stro.cus.	Ubud	Indonesia	without lime	1	1	0,9595	18,218	1	0,00505	36,08	dark blue / violet	10,5	2,25	-3,95
**AsB.94**	**ADD.26**	Stro.cus.	Ubud	Indonesia	without lime	1	2	0,9716	18,505	1	0,00505	36,64	dark blue / violet	10,5	2,25	-3,95
**AsB.94**	**ADD.26**	Stro.cus.	Ubud	Indonesia	without lime	2	1	1,0059	18,059	1	0,00534	33,82	dark blue / violet	10,5	2,25	-3,95
**AsB.94**	**ADD.26**	Stro.cus.	Ubud	Indonesia	without lime	2	2	1,006	18,062	1	0,00534	33,82	dark blue / violet	10,5	2,25	-3,95
**AsB.94**	**ADD.26**	Stro.cus.	Ubud	Indonesia	without lime	3	1	1,0116	19,174	1	0,00514	37,30	dark blue / violet	10,5	2,25	-3,95
**AsB.94**	**ADD.26**	Stro.cus.	Ubud	Indonesia	without lime	3	2	1,0186	19,343	1	0,00514	37,63	dark blue / violet	10,5	2,25	-3,95
**AsB.95**	**ADD.27**	Stro.cus.	Ubud	Indonesia	without lime	1	1	0,882	16,385	1	0,00506	32,38	dark blue / violet	12,25	0,14	-7,54
**AsB.95**	**ADD.27**	Stro.cus.	Ubud	Indonesia	without lime	1	2	0,8591	15,843	1	0,00506	31,31	dark blue / violet	12,25	0,14	-7,54
**AsB.95**	**ADD.27**	Stro.cus.	Ubud	Indonesia	without lime	2	1	0,8412	14,044	1	0,00513	27,38	dark blue / violet	12,25	0,14	-7,54
**AsB.95**	**ADD.27**	Stro.cus.	Ubud	Indonesia	without lime	2	2	0,8566	14,419	1	0,00513	28,11	dark blue / violet	12,25	0,14	-7,54
**AsB.95**	**ADD.27**	Stro.cus.	Ubud	Indonesia	without lime	3	1	0,9241	17,062	1	0,00520	32,81	dark blue / violet	12,25	0,14	-7,54
**AsB.95**	**ADD.27**	Stro.cus.	Ubud	Indonesia	without lime	3	2	0,9395	17,434	1	0,00520	33,53	dark blue / violet	12,25	0,14	-7,54
