# Supplementary material for: “Purplish Blue” or “Greenish Grey”? Indigo Qualities and Extraction Yields from Six Species
Source: Plants (Basel). 2024 Mar 22;13(7):918. doi: 10.3390/plants13070918 (PMC11013892; doi:10.3390/plants13070918)
Supplement: Supplementary file 1 [file plants-13-00918-s001.zip › Table S3.pdf]

Table S3. Raw data of samples used for spectrophotometric method validation

AsB-No.	Sample code	Species	Provenance	Cultivation	Type of extraction	Extraction replication	Sample preparation	Duplicates	Raw data (= Absorp-tion)	Single conc. (µg/ml)	Dilution factor	Indigo weight (g)	Indigotin (%)	
**AsB.66**	**sLPE.10**	Pers.tinc.	"Maruba"	Austria	sLPE	1	1	1	0,3246	1,5694	1	0,00505	3,11	intra day
**AsB.66**	**sLPE.10**	Pers.tinc.	"Maruba"	Austria	sLPE	1	1	2	0,3341	1,8152	1	0,00505	3,59	intra day
**AsB.66**	**sLPE.10**	Pers.tinc.	"Maruba"	Austria	sLPE	1	2	1	0,3283	1,6652	1	0,00524	3,18	intra day
**AsB.66**	**sLPE.10**	Pers.tinc.	"Maruba"	Austria	sLPE	1	2	2	0,3295	1,6962	1	0,00524	3,24	intra day
**AsB.66**	**sLPE.10**	Pers.tinc.	"Maruba"	Austria	sLPE	1	3	1	0,3256	1,5953	1	0,00507	3,15	intra day
**AsB.66**	**sLPE.10**	Pers.tinc.	"Maruba"	Austria	sLPE	1	3	2	0,3278	1,6522	1	0,00507	3,26	intra day
**AsB.39**	**MME.51**	Ind.tinc.	Maldives	Austria	MME	1	1	1	0,65	9,9896	1	0,00517	19,32	intra day
**AsB.39**	**MME.51**	Ind.tinc.	Maldives	Austria	MME	1	1	2	0,6503	9,9974	1	0,00517	19,34	intra day
**AsB.39**	**MME.51**	Ind.tinc.	Maldives	Austria	MME	1	2	1	0,6391	9,7076	1	0,00506	19,18	intra day
**AsB.39**	**MME.51**	Ind.tinc.	Maldives	Austria	MME	1	2	2	0,6467	9,9043	1	0,00506	19,57	intra day
**AsB.39**	**MME.51**	Ind.tinc.	Maldives	Austria	MME	1	3	1	0,6728	10,556	1	0,00530	19,92	intra day
**AsB.39**	**MME.51**	Ind.tinc.	Maldives	Austria	MME	1	3	2	0,674	10,586	1	0,00530	19,97	intra day
**AsB.32**	**MME.38**	Pers.tinc.	"Maruba"	Austria	MME	3	1	1	1,2765	25,037	1	0,00505	49,58	intra day
**AsB.32**	**MME.38**	Pers.tinc.	"Maruba"	Austria	MME	3	1	2	1,2899	25,339	1	0,00505	50,18	intra day
**AsB.32**	**MME.38**	Pers.tinc.	"Maruba"	Austria	MME	3	2	1	1,3107	25,807	1	0,00523	49,34	intra day
**AsB.32**	**MME.38**	Pers.tinc.	"Maruba"	Austria	MME	3	2	2	1,3278	26,192	1	0,00523	50,08	intra day
**AsB.32**	**MME.38**	Pers.tinc.	"Maruba"	Austria	MME	3	3	1	1,2881	25,298	1	0,00510	49,60	intra day
**AsB.32**	**MME.38**	Pers.tinc.	"Maruba"	Austria	MME	3	3	2	1,2982	25,526	1	0,00510	50,05	intra day
**AsB.66**	**sLPE.10**	Pers.tinc.	"Maruba"	Austria	sLPE	1	1	1	0,3173	1,8884	1	0,00528	3,58	inter day
**AsB.66**	**sLPE.10**	Pers.tinc.	"Maruba"	Austria	sLPE	1	1	2	0,3154	1,8388	1	0,00528	3,48	inter day
**AsB.66**	**sLPE.10**	Pers.tinc.	"Maruba"	Austria	sLPE	1	2	1	0,3156	2,088	1	0,00505	4,13	inter day
**AsB.66**	**sLPE.10**	Pers.tinc.	"Maruba"	Austria	sLPE	1	2	2	0,3195	2,1965	1	0,00505	4,35	inter day
**AsB.66**	**sLPE.10**	Pers.tinc.	"Maruba"	Austria	sLPE	1	3	1	0,3466	2,6833	1	0,00524	5,12	inter day
**AsB.66**	**sLPE.10**	Pers.tinc.	"Maruba"	Austria	sLPE	1	3	2	0,3455	2,6547	1	0,00524	5,07	inter day
**AsB.39**	**MME.51**	Ind.tinc.	Maldives	Austria	MME	1	1	1	0,6505	10,103	1	0,00506	19,97	inter day
**AsB.39**	**MME.51**	Ind.tinc.	Maldives	Austria	MME	1	1	2	0,6389	9,8167	1	0,00506	19,40	inter day
**AsB.39**	**MME.51**	Ind.tinc.	Maldives	Austria	MME	1	2	1	0,7342	11,974	1	0,00528	22,68	inter day
**AsB.39**	**MME.51**	Ind.tinc.	Maldives	Austria	MME	1	2	2	0,7074	11,056	1	0,00528	20,94	inter day
**AsB.39**	**MME.51**	Ind.tinc.	Maldives	Austria	MME	1	3	1	0,6455	9,7796	1	0,00509	19,21	inter day
**AsB.39**	**MME.51**	Ind.tinc.	Maldives	Austria	MME	1	3	2	0,6534	9,9699	1	0,00509	19,59	inter day
**AsB.32**	**MME.38**	Pers.tinc.	"Maruba"	Austria	MME	3	1	1	1,3382	28,761	1	0,00527	54,57	inter day
**AsB.32**	**MME.38**	Pers.tinc.	"Maruba"	Austria	MME	3	1	2	1,2833	27,669	1	0,00527	52,50	inter day
**AsB.32**	**MME.38**	Pers.tinc.	"Maruba"	Austria	MME	3	2	1	1,2528	27,354	1	0,00525	52,10	inter day
**AsB.32**	**MME.38**	Pers.tinc.	"Maruba"	Austria	MME	3	2	2	1,1632	25,037	1	0,00525	47,69	inter day
**AsB.32**	**MME.38**	Pers.tinc.	"Maruba"	Austria	MME	3	3	1	1,3062	26,922	1	0,00523	51,48	inter day
**AsB.32**	**MME.38**	Pers.tinc.	"Maruba"	Austria	MME	3	3	2	1,3203	27,299	1	0,00523	52,20	inter day
